# Supplementary material for: Thresholds for clinically important deterioration versus improvement in COPD health status: results from a randomised controlled trial in pulmonary rehabilitation and an observational study during routine clinical practice
Source: BMJ Open. 2019 Jun 28;9(6):e025776. doi: 10.1136/bmjopen-2018-025776 (PMC6609082; doi:10.1136/bmjopen-2018-025776)
Supplement: Author's manuscript [file bmjopen-2018-025776.draft_revisions.pdf]

BMJ Open

BMJ Open is committed to open peer review. As part of this commitment we make the peer review history of every article we publish publicly available.

When an article is published we post the peer reviewers' comments and the authors' responses online. We also post the versions of the paper that were used during peer review. These are the versions that the peer review comments apply to.

The versions of the paper that follow are the versions that were submitted during the peer review process. They are not the versions of record or the final published versions. They should not be cited or distributed as the published version of this manuscript.

BMJ Open is an open access journal and the full, final, typeset and author-corrected version of record of the manuscript is available on our site with no access controls, subscription charges or pay-per-view fees (http://bmjopen.bmj.com).

If you have any questions on BMJ Open's open peer review process please email info.bmjopen@bmj.com

# Thresholds for Clinically Important Deterioration versus Improvement in COPD Health Status during Pulmonary Rehabilitation and Routine Clinical Practice: A Retrospective Analysis


| | |
|---|---|
| Journal: | *BMJ Open* |
| Manuscript ID | bmjopen-2018-025776 |
| Article Type: | Research |
| Date Submitted by the Author: | 12-Aug-2018 |



| | |
|---|---|
| Complete List of Authors: | Alma, Harma; University of Groningen, University Medical Center Groningen, Department of General Practice and Elderly Care Medicine; University of Groningen, University Medical Center Groningen, Groningen Research Institute for Asthma and COPD (GRIAC)<br>de Jong, Corina; University of Groningen, University Medical Center Groningen, Department of General Practice and Elderly Care Medicine; University of Groningen, University Medical Center Groningen, Groningen Research Institute for Asthma and COPD (GRIAC)<br>Jelusic, Danijel; Klinik Bad Reichenhall, Center for Rehabilitation, Pulmonology and Orthopedics<br>Wittmann, Michael; Klinik Bad Reichenhall, Center for Rehabilitation, Pulmonology and Orthopedics<br>Schuler, Michael; Julius-Maximilians-Universitat Wurzburg, Medical Psychology and Psychotherapy, Medical Sociology and Rehabilitation Sciences<br>Sanderman, Robbert; University of Groningen, University Medical Center Groningen, Department of Health Psychology; University of Twente, Department of Psychology, Health and Technology<br>Schultz, Konrad; Klinik Bad Reichenhall, Center for Rehabilitation, Pulmonology and Orthopedics<br>Kocks, Janwillem; University of Groningen, University Medical Center Groningen, Department of General Practice and Elderly Care Medicine; University of Groningen, University Medical Center Groningen, Groningen Research Institute for Asthma and COPD (GRIAC)<br>van der Molen, Thys; University of Groningen, University Medical Center Groningen, Department of General Practice and Elderly Care Medicine; University of Groningen, University Medical Center Groningen, Groningen Research Institute for Asthma and COPD (GRIAC) |



| | |
|---|---|
| Keywords: | Chronic Obstructive Pulmonary Disease (COPD), Health-Related Quality of Life (HRQoL), Health Status Responsiveness, Pulmonary Rehabilitation (PR), Routine Clinical Practice (RCP), Minimal Clinically Important Difference (MCID) |

For peer review only

# Thresholds for Clinically Important Deterioration versus Improvement in COPD Health Status during Pulmonary Rehabilitation and Routine Clinical Practice: A Retrospective Analysis

Harma Alma[ab], MD MSc; Corina de Jong, PhD[ab]; Danijel Jelusic[c], MD; Michael Wittmann[c], MD; Michael Schuler[d], PhD; Prof. dr. Robbert Sanderman[ef], PhD; Konrad Schultz, MD[c]; Janwillem Kocks[ab], MD, PhD; Prof. dr. Thys van der Molen[ab,] MD, PhD

[a] University of Groningen, University Medical Center Groningen, Department of General Practice and Elderly Care Medicine, Groningen, The Netherlands

[b] University of Groningen, University Medical Center Groningen, Groningen Research Institute for Asthma and COPD (GRIAC), Groningen, The Netherlands

[c] Klinik Bad Reichenhall, Center for Rehabilitation, Pulmonology and Orthopedics, Bad Reichenhall, Germany

[d] University of Wuerzburg, Department of Medical Psychology and Psychotherapy, Medical Sociology and Rehabilitation Sciences, Wuerzburg, Germany

[e] University of Groningen, University Medical Center Groningen, Department of Health Psychology, Groningen, The Netherlands

[f] University of Twente, Department of Psychology, Health and Technology, Enschede, The Netherlands

| | |
|---|---|
| Harma Alma | h.j.alma@umcg.nl |
| Corina de Jong | c.de.jong02@umcg.nl |
| Danijel Jelusic | djelusic87@gmail.com |
| Michael Wittmann | michael.wittmann@klinik-bad-reichenhall.de |
| Michael Schuler | m.schuler@uni-wuerzburg.de |
| Robbert Sanderman | r.sanderman@umcg.nl |
| Konrad Schultz | konrad.schultz@klinik-bad-reichenhall.de |
| Janwillem Kocks | j.w.h.kocks@umcg.nl |
| Thys van der Molen | t.van.der.molen@umcg.nl |

Corresponding author:

Mrs. H.J. Alma, MD MSc
University of Groningen, University Medical Center Groningen
Department of General Practice and Elderly Care Medicine,
HPC FA21, Postbox 196
NL-9700 AD Groningen
T: +31 50 3616742 or +31 6 25649635 or +31 6 27137024
E: h.j.alma@umcg.nl

Total word count manuscript: 3862 (Abstract 292)



For peer review only

For peer review only - http://bmjopen.bmj.com/site/about/guidelines.xhtml

## Abstract

**Introduction:**

Until now, it is unclear whether (Minimal) Clinically Important Differences ((M)CIDs) are similar for both deterioration and improvement in health status. This retrospective study investigated clinically relevant thresholds for deterioration versus improvement for three widely used health status questionnaires in COPD.

**Methods:**

COPD patients GOLD II-IV aged ≥18 years without respiratory co-morbidities were recruited during a 3-week Pulmonary Rehabilitation (PR) randomized controlled trial in the Klinik Bad Reichenhall in Germany. GOLD I-IV patients aged ≥40 years with similar exclusion criteria were recruited from Dutch primary and secondary Routine Clinical Practice (RCP). The COPD Assessment Test (CAT), Clinical COPD Questionnaire (CCQ) and St. George's Respiratory Questionnaire (SGRQ) were completed at baseline, three, six, and 12 months. A 15-point Global Rating of Change scale (GRC) was added at each follow-up moment. Anchor-based- (GRC) and distribution-based (half Standard Deviation) methods were used to determine clinically relevant thresholds.

**Results:**

In total, 451 patients were included from PR (57.87±6.56 years, 65% male, 50/39/11% GOLD II/III/IV) and 207 patients from RCP (66.69±7.91, 58.5% male and 17/40/30/12% GOLD I/II/III/IV). MCIDs for deterioration ranged 1.30-4.21 (CAT), 0.19-0.66 (CCQ), and 2.75-7.53 (SGRQ). MCIDs for improvement ranged respectively -3.78 to -1.53, -0.50 to -0.19, and -9.20 to -2.76. Weighted thresholds for moderate improvement and deterioration were -4.23 and 3.89-7.06 (CAT), -0.82 and 0.62-1.23 (CCQ), and -16.06 and 7.46-9.30 (SGRQ).

**Conclusions:**

MCID ranges for improvement and deterioration on the CAT, CCQ and SGRQ were somewhat similar. However, estimates for moderate and large change varied and were inconsistent. Thresholds differed between study settings.

**Trial registration number:**

PR patients were recruited from the RIMTCORE trial (#DRKS00004609 and #12107 Ethik-Kommission der Bayerischen Landesärztekammer). Dutch RCP patients were recruited from the MCID study registered at the University Medical Center Groningen (UMCG) Research Register (#201500447).




For peer review only - http://bmjopen.bmj.com/site/about/guidelines.xhtml

## Article Summary

**Strengths:**

- Our study is the first dedicated investigation of (Minimal) Clinically Important Differences ((M)CIDs) for deterioration on COPD health status tools.
- Our study used a combination of anchor- and distribution-based methods to determine clinically relevant thresholds for both deterioration and improvement.
- Our study investigated clinically relevant thresholds in two different study settings - Pulmonary Rehabilitation (PR) and Routine Clinical Practice (RCP) - by using data from various follow-up periods to minimize possible impact of the recall period.

**Limitations:**

- Our study included a rather limited number of patients with deterioration after their PR intervention and during RCP.
- Our study found broad ranges and wide confidence intervals for the (M)CIDs of COPD health status tools, requiring possibly larger sample sizes for more accuracy.

For peer review only

# Declarations

## Funding

The main RIMTCORE trial (#DRKS00004609) was funded by the *Deutsche Rentenversicherung*. The Dutch observational study on COPD health status in routine clinical practice as well as the current combined retrospective analysis of both studies received financial support from the Junior Scientific Masterclass (JSM) as part of the University of Groningen.

## Competing interests

H.J. Alma, C. de Jong, D. Jelusic, M. Wittmann, M. Schuler and R. Sanderman have nothing to disclose. J.W.H. Kocks reports personal fees from Novartis; research grants and personal fees from Boehringer Ingelheim; research grants and personal fees from GSK; research grants from Stichting Zorgdraad; personal fees from IPCRG; personal fees from Springer Media; and travel arrangements from Chiesi BV, GlaxoSmithKline BV, and IPCRG, all outside the submitted work. K. Schultz received lecture fees from Boehringer, AstraZeneca, Berlin Chemie, Novartis, Chiesi, Mundipharma, Takeda, GSK and MSD, all outside the submitted work. T. van der Molen reports personal reimbursements from GSK, TEVA, Astra Zeneca, Boehringer Ingelheim and study grants from Astra Zeneca and GSK. After this study was terminated, he became employee of GSK. None of these stated conflicts of interest are linked to the current manuscript. T. van der Molen developed the CCQ and holds the copyright.

## Authors' contributions

KS, MW, DJ and MS planned the RIMTCORE study design and were responsible for data collection. HA, CdJ, RS and TvdM designed the Dutch observational study on COPD health status in routine clinical practice as well as the current retrospective analysis of both studies. HA and CdJ performed the statistical analysis. HA wrote the first draft, while CdJ, JK, RS and TvdM actively participated in the review process. RS and TvdM supervised and participated in different steps of the study, as well as in writing. All authors participated in and approved of the final version of the manuscript

## Consent of publication

All authors participated in various steps in the study, edited the manuscript and gave their approval for submission.



For peer review only - http://bmjopen.bmj.com/site/about/guidelines.xhtml

## Ethics approval and consent to participate

This retrospective study is a secondary analysis of a subsample from the Routine Inspiratory Muscle Training within COPD Rehabilitation (RIMTCORE) real-life randomized controlled trial (#DRKS00004609) in the Klinik Bad Reichenhall, Center for Rehabilitation, Pulmonology and Orthopaedics in Germany; and a primary analysis of all patients participating in the Dutch observational trial (MCID study) on COPD health status in routine clinical practice (UMCG trial #201500447). All patients in both studies signed informed consent upon participation. The RIMTCORE trial has been approved by the Ethik-Kommission der Bayerischen Landesärztekammer and registered in the German Clinical Trial Register. The MCID study has been registered at the University Medical Center Groningen (UMCG) Research Register and evaluated by its Medical Ethical Committee.

## Data Sharing Statement

The data that support the findings of this study are not publicly available. Participating patients in the RIMTCORE trial have only agreed upon availability of their data to the Klinik Bad Reichenhall, their scientific partners in the data analysis and the Committee of the Bavarian State Chamber of Labor in Munich. Participating patients in the MCID study only agreed upon availability of their data to the University Medical Center Groningen (UMCG) and their scientific partners in the data analysis.

## Acknowledgements

We are grateful to the Junior Scientific Masterclass of the University of Groningen, who financially supported the research position of the first author. We would also like to acknowledge all participating patients in both the RIMTCORE trial and the MCID study.

For peer review only

**Key Words**

Chronic Obstructive Pulmonary Disease (COPD)

Health-Related Quality of Life (HRQoL)

Health Status Responsiveness

Pulmonary Rehabilitation (PR)

Routine Clinical Practice (RCP)

Minimal Clinically Important Difference (MCID)

For peer review only

# Article manuscript

## Introduction

The use of health status questionnaires is recommended by the Global Initiative for Chronic Obstructive Lung Disease (GOLD) for the assessment, evaluation and management of patients with Chronic Obstructive Pulmonary Disease (COPD) [1]. The COPD Assessment Test (CAT) [2], the Clinical COPD Questionnaire (CCQ) [3], and the St. George's Respiratory Questionnaire (SGRQ) [4] are frequently used health status tools important for clinical practice and scientific research [5] as the burden of COPD is high worldwide [6-7].

Various studies have examined clinically relevant thresholds for change on the CAT, CCQ and SGRQ in order to be able to evaluate and interpret treatment effects [8-18]. The Minimal Clinically Important Difference (MCID) is a parameter that quantifies this threshold. It has been defined as *"the smallest difference in score, which patients perceive as beneficial and which would mandate a change in the patient's management"* [19]. Change exceeding the level of the MCID can be considered clinically relevant, thus justifying therapy and help developing guidelines. It is pivotal that clinically relevant thresholds for change on a health status tool are rigorously studied and analysed carefully.

Most clinical studies that examine the MCID of Patient-Reported Outcomes (PROs) are executed in the context of an intervention such as pharmacotherapy or Pulmonary Rehabilitation (PR). This usually results in an improvement in the patients' Health-Related Quality of Life (HRQoL). However, it remains unclear to what extent clinically relevant thresholds for improvement are similar to those for deterioration [20-23]. Determining deterioration in HRQoL is of importance, since one needs to differentiate between real worsening of a patient's status and random variations. Next, the effects of therapy may also halt further deterioration of a progressive disease; so no relevant worsening or a reduction in clinically relevant deterioration over time might also be considered a success of therapy and in clinical trials [24]. Some studies outside the field of COPD have analysed the MCIDs of PROs and found evidence that values for improvement differed from deterioration [25-29]. On the other hand, there is also evidence that thresholds might be similar [30].

In COPD health status, the estimated MCID is for the CAT 2.00-3.00 [11-15]; for the CCQ 0.40-0.50 [8-13]; and for the SGRQ 4.00-8.00 [12, 16-18]. This is valid for improvement only, as there were too few patients with deterioration to investigate. There are no studies that specifically investigate clinically relevant thresholds for deterioration on these PROs. This study therefore aimed to determine clinically relevant thresholds for



For peer review only - http://bmjopen.bmj.com/site/about/guidelines.xhtml

improvement and deterioration on the COPD health status questionnaires CAT, CCQ and SGRQ in a PR and Routine Clinical Practice (RCP) setting.

For peer review only



For peer review only - http://bmjopen.bmj.com/site/about/guidelines.xhtml

## Patients and methods

### Study subjects

This study was a retrospective analysis of data obtained from two clinical trials. Study one was a secondary analysis of a subsample from the Routine Inspiratory Muscle Training within COPD Rehabilitation (RIMTCORE) real-life randomized controlled trial in the Klinik Bad Reichenhall, Center for Rehabilitation, Pulmonology and Orthopedics in Germany [31]. Patients were included between February 2013 and July 2014. A sample was selected of COPD participants GOLD II-IV aged ≥18 years, who gave informed consent, without other respiratory co-morbidities (e.g. bronchiectasis, asthma, history of bronchial carcinoma, sarcoidosis, tuberculosis); or alpha-1-antitrypsin deficiency [12]. Study two (MCID study) was an observational trial of COPD patients GOLD I-IV aged ≥40 years without respiratory co-morbidities or alpha-1-antitrypsin deficiency in Dutch primary and secondary routine clinical practice. Patients provided written informed consent. Patients were recruited between September 2015 and September 2016 from various general practices, hospitals and the Dutch patient lung federation. The study was evaluated by the Medical Ethical Committee of the University Medical Center Groningen (UMCG), the Netherlands.

### Patient and public involvement

In both studies, patients and the public have not actively been involved during the design of the study nor the assessment of the burden of intervention. Summary results are disseminated to participating patients after study completion.

### Study design and data collection

Patients in study one participated in an intensive 3-week full-day inpatient PR program tailored to the patient's individual needs. Details have been presented prior [12, 31]. Patient descriptives and post-bronchodilator spirometry were collected at baseline and discharge. Patients in study two received routine care from their physician according to national treatment guidelines. Evaluation of health status over a 12-months period was the primary measurement outcome. Patient descriptives and spirometry data were obtained at baseline.

Primary outcomes selected from both studies for this retrospective analysis were the CAT (no recall period), CCQ (weekly version) and SGRQ (monthly version). In study one, these questionnaires were collected at baseline, at PR discharge and during follow-up at three, six, nine and 12 months. Baseline and discharge measurements were taken in the clinic, where patients were blinded to their baseline scores. Follow-up questionnaires were send by mail. In study two, questionnaires were send by mail and scored at home at



For peer review only - http://bmjopen.bmj.com/site/about/guidelines.xhtml

baseline, three, six and 12 months. For this retrospective analysis baseline scores, and follow-up measurements at three, six and 12 months were included, to allow for sufficient time for deterioration in HRQoL, to include various time periods of measurement, and to allow for comparison between both studies.

The CAT is an eight-item one-dimensional scale with item scores ranging 0-5 (0: no impairment, 5: maximum impairment) and a total score summing up to a maximum of 40 [2]. The CCQ consists of ten items scoring 0-6 (0: no impairment, 6: maximum impairment) [3]. The items cover the domains symptoms (four items), functional status (four items) and mental status (two items). Total and domain scores on the CCQ derive from adding up relevant item scores and dividing this by the number of items. The SGRQ has 50 items classified into the domains symptoms (eight items), activities (16 items) and impact (26 items) [4]. Domain and total SGRQ scores can range from 0-100 (0: no impairment, 100: maximum impairment). A 15-point Likert scale anchor question (Global Rating of Change GRC) was scored by the patient at each follow-up measurement in both datasets. The GRC required patients to assess their COPD health state compared to baseline. The answers were marked on a scale from -7 to +7, ranging from *very much worse* to *very much better* and zero equalling *no change* [32-33].

**Study methods**

All change scores for the total scores of the CAT, CCQ and SGRQ were calculated as the difference between baseline and the respective follow-up moment (three, six and 12 months). Negative change on all questionnaires represented improvement, positive change deterioration. First, in the anchor-based approach, changes on the health status instruments were classified using the corresponding score on GRC question. Scores of 0 and ±1 on the GRC indicated *no change*; scores of ±2 and ±3 represented a *minimal improvement/deterioration*; scores of ±4 and ±5 were summarized as a *moderate improvement/deterioration*; and scores of ±6 and ±7 indicated a *large improvement/deterioration* [32-33]. MCID estimates for both improvement and deterioration on the CAT, CCQ and SGRQ were calculated as the mean change scores including 95% Confidence Interval (95%CI) of those patients indicating a minimal improvement/deterioration (±2 and ±3) on the GRC for each follow-up moment, verifying normality of distribution. Mean estimates including 95%CI were determined in a similar way for patients indicating no change (GRC 0 and ±1), moderate change (GRC ±4 and ±5) and large change (GRC ±6 and ±7) [32-33]. Second, the distribution-based method half Standard Deviation (0.5 SD) of the change score was calculated for improved and deteriorating health status patients at respective follow-up periods [34].

For peer review only



For peer review only - http://bmjopen.bmj.com/site/about/guidelines.xhtml

**Data analysis**

Data analysis was performed using SPSS 24.0 (IBM, Chicago, USA). Descriptives were evaluated at baseline for either frequencies with percentages (%), mean with Standard Deviation (SD) or median with range. This was depending on the variable characteristics and/or normality of distribution. Health status data on the CCQ, CAT and SGRQ were evaluated at baseline (T0), three months (T2), six months (T3) and after 12 months (T5). Normality of distribution was verified using skewness and kurtosis. Values between -1 and +1 were considered indicative for normality. Data were checked for floor- and ceiling effects defined as over 15% of patients scoring in the lowest and highest 10% of the maximum scale range [35]. Mean and standard deviations (or median and range) were calculated at each measurement moment for all patients, as well as specifically for patients with improved and deteriorated health status change scores. Baseline scores were compared between improving and deteriorating patients, and tested using independent t-tests after verifying normality of distribution. Baseline scores were compared between both datasets using independent t-tests, Man-Whitney U tests or Chi-Square tests depending on the variable characteristic and/or normality of distribution. Health status change scores were all calculated in comparison to baseline. Follow-up scores were compared with baseline to test for significance of change using paired t-tests verifying normality of distribution.

In order to determine the clinically relevant thresholds for change, first correlations between the GRC and the CCQ, CAT and SGRQ were assessed using Pearson or Spearman correlation coefficients depending on normality of distribution. Correlations needed to be $\geq$0.30 (preferably $\geq$0.50) to be eligible as anchor [21]. Correlations were not only assessed between GRC and questionnaire change scores, but also between GRC, baseline and follow-up questionnaire score to assess for a possible *response shift*. Next, participants were categorized according to their GRC score at each follow-up moment. Mean changes (95%CI) for each respective category were determined to define thresholds for clinically relevant change. Significance of change for each GRC class at the respective follow-up moment was compared to baseline and assessed with paired t-tests verifying normality of the data. Last, the 0.5SD of the change score was determined for patients with improved and deteriorating health status change scores separately at each follow-up moment.

An absolute overall weighted mean MCID estimate for both improvement and deterioration was calculated at the end by multiplying the number of observations (n) at each follow-up moment times the MCID estimate for that period. The sum was divided by the total number of observations. Anchor-based and distribution-based approaches had similar weights. Estimates for improvement and deterioration were compared visually in a plot.



For peer review only - http://bmjopen.bmj.com/site/about/guidelines.xhtml

## Results

### Patient characteristics

Study one included 451 patients with completed baseline data [12, 31]. During follow-up 355 patients (78.7%) had completed data at T2; 319 patients (70.7%) at T3; and 309 patients (68.5%) at T5. During the 12-months follow-up eight patients passed away according to the administrative records, 41 dropped out at own request and a varying number of non-response was present. Mean age was 57.87±6.56, 65% was male and 50/39/11% GOLD II/III/IV (Table 1).

Study two included 207 patients with full baseline data, of whom 201 (97.1%) completed the three-months follow-up, 186 (89.9%) six-months follow-up and 177 (85.6%) 12-months follow-up. Four patients died according to the administrative records knowledge, 12 patients discontinued at own request and a various number of non-response was present. Mean age was 66.69±7.91, 58.5% male and 17/40/30/12% GOLD I/II/III/IV (Table 1).

There were no significant baseline differences between completers and non-completers of the 12-months follow-up in both studies, except that significantly more females (28.4%) compared with men (10.0%) did not complete the follow-up during RCP. Significant differences in age, Forced Expiratory Volume in one second percentage predicted (FEV1%pred) and health status were observed between both studies (Table 1).

*Table 1: Baseline patient characteristics*

| | **Study 1: PR** | **Study 2: RCP** | **Significance testing** |
|---|---|---|---|
| N (number of patients) | 451 | 207 | - |
| Age (years)[a] | 57.87 ± 6.56 | 66.69 ± 7.91 | *P < 0.001** |
| Gender (male)[b] | 293 (65.0) | 121 (58.5) | *P = 0.507* |
| FEV1%pred[a] | 50.40 ± 15.11 | 57.06 ± 21.96 | *P = 0.001** |
| GOLD I[b]<br>GOLD II<br>GOLD III<br>GOLD IV | -<br>227 (50.3)<br>176 (39.0)<br>48 (10.6) | 35 (17.4)<br>80 (39.8)<br>61 (30.3)<br>25 (12.4) | *P = 0.199* |
| Smoking pack years[a] | 40 (30-50) | 37.5 (22.50-51.25) | *P = 0.081* |
| CAT Total[a] | 20.23 ± 7.33 | 18.32 ± 7.22 | *P = 0.002** |
| CCQ Total[a] | 2.86 ± 1.17 | 2.12 ± 1.02 | *P < 0.001** |
| CCQ Symptoms[a] | 2.87 ± 1.24 | 2.48 ± 1.03 | *P < 0.001** |
| CCQ Functional Status[a] | 2.86 ± 1.34 | 2.28 ± 1.40 | *P < 0.001** |
| CCQ Mental Status[a] | 2.86 ± 1.74 | 1 (0-1.50) | *P < 0.001** |
| SGRQ Total[a] | 50.69 ± 17.33 | 42.88 ± 19.16 | *P < 0.001** |
| SGRQ Symptoms[a] | 63.66 ± 21.77 | 48.04 ± 24.16 | *P < 0.001** |
| SGRQ Activities[a] | 63.58 ± 19.82 | 61.48 ± 21.10 | *P = 0.259* |
| SGRQ Impact[a] | 39.21 ± 18.81 | 30.52 ± 19.73 | *P < 0.001** |
| mMRC[a] | 2 (2-4) | 1 (1-2) | *P < 0.001** |

[I] Data were expressed as mean ± standard deviation or median (IQR).
[II] Data were expressed as frequencies (% of total).

* Significance testing at level $p < 0.05$ using unpaired T-tests, Man Whitney-U tests or Chi Square tests.

*Abbreviations:* CAT, COPD Assessment Test; CCQ, Clinical COPD Questionnaire; FEV1%pred, Forced Expiratory Volume in one

For peer review only

Second % predicted; GOLD, Global Initiative for Chronic Obstructive Lung Disease; mMRC, modified Medical Research Council Dyspnea Scale; N, Number of Patients; PR, Pulmonary Rehabilitation; RCP, Routine Clinical Practice; SGRQ, St. George's Respiratory Questionnaire

***Health status scores for improvement and deterioration***

In study one and two, CAT, CCQ and SGRQ total were normally distributed at baseline and follow-up. Completed pairs of change scores (follow-up vs. baseline) were included (pair-wise deletion). Floor- and ceiling effects were negligible. Mean health status baseline scores were significantly different for PR and RCP, respectively 20.23±7.33 vs. 18.32 ± 7.22 (CAT), 2.86±1.17 vs. 2.12 ± 1.02 (CCQ), and 50.69±17.33 vs. 42.88 ± 19.16 (SGRQ) (Table 1). In general, 58-59% of patients had improved health status scores (negative change) at 12 months follow-up after PR; compared with 44-46% during RCP (Table 2).

Mean changes 12 months after PR were -5.45±4.66 for improvers and 5.47±4.22 for patients with deteriorating health status on the CAT; -0.87±0.72 for improvement and 0.83±0.62 for deterioration on the CCQ; and -13.83±10.43 for improvers and 10.19±8.94 for deterioration on the SGRQ (Table 2). These estimates were in RCP -4.53±3.15 for improvement and 3.88±2.59 for deterioration on the CAT; -0.54±0.54 for improvement and 0.51±0.39 for deterioration on the CCQ; and -7.74±9.51 for improvement on the SGRQ and 8.46±7.06 for deterioration (Table 2). Mean change at 12 months follow-up after PR were significant with -0.89 (CAT), -0.16 (CCQ) and -3.94 (SGRQ). Health status changes during one year routine clinical practice were not significant.

There were no baseline differences in terms of age, gender and GOLD classification between improved health status patients and those who deteriorated at 12 months in both studies. Patients with a worse CAT, CCQ or SGRQ baseline score prior to PR had significantly more improved health status after one year. Patients, who improved during RCP, had a significantly higher baseline FEV1%pred.

For peer review only



For peer review only - http://bmjopen.bmj.com/site/about/guidelines.xhtml

*Table 2: Health status baseline and change scores for all, improved and deteriorated patients during PR and Routine Clinical Practice (RCP)*

| | Change after 3 months (T2) | N | Change after 6 months (T3) | N | Change after 12 months (T5) | N |
|---|---|---|---|---|---|---|
| **CAT** | | | | | | |
| *All patients PR* | -1.44* (-2.16 to -0.71) | 354 | -0.91* (-1.66 to -0.16) | 319 | -0.89* (-1.68 to -0.11) | 309 |
| *Improvement PR* | -5.45±4.57 | 227 (64.1) | -5.49±4.33 | 184 (57.7) | -5.45±4.66 | 180 (58.3) |
| *Deterioration PR* | 5.75±4.20 | 127 (35.9) | 5.33±4.10 | 135 (42.3) | 5.47±4.22 | 129 (41.7) |
| *All patients RCP* | 0.30 (-0.42 to +1.02) | 201 | 0.18 (-0.53 to +0.90) | 186 | 0.14 (-0.59 to +0.87) | 177 |
| *Improvement RCP* | -4.04±3.33 | 102 (50.7) | -4.64±3.05 | 81 (43.5) | -4.53±3.15 | 79 (44.6) |
| *Deterioration RCP* | 4.23±3.66 | 83 (41.3) | 3.76±2.88 | 91 (48.9) | 3.88±2.59 | 86 (48.6) |
| *No change RCP* | - | 16 (8.0) | - | 14 (7.5) | - | 12 (6.8) |
| **CCQ Total** | | | | | | |
| *All patients PR* | -0.26* (-0.37 to -0.15) | 355 | -0.11 (-0.23 to +0.01) | 319 | -0.16* (-0.28 to -0.04) | 309 |
| *Improvement PR* | -0.88±0.71 | 225 (63.4) | -0.84±0.68 | 181 (56.7) | -0.87±0.72 | 180 (58.3) |
| *Deterioration PR* | 0.82±0.68 | 130 (36.6) | 0.84±0.67 | 138 (43.3) | 0.83±0.62 | 129 (41.7) |
| *All patients RCP* | 0.00 (-0.09 to +0.08) | 200 | 0.00 (-0.10 to +0.10) | 185 | -0.02 (-0.12 to +0.09) | 174 |
| *Improvement RCP* | -0.45±0.37 | 96 (48.0) | -0.52±0.51 | 87 (47.0) | -0.54±0.54 | 77 (44.3) |
| *Deterioration RCP* | 0.50±0.38 | 89 (44.5) | 0.56±0.46 | 80 (43.2) | 0.51±0.39 | 88 (50.6) |
| *No change RCP* | - | 15 (7.5) | - | 18 (9.7) | - | 9 (5.2) |
| **SGRQ Total** | | | | | | |
| *All patients PR* | -5.35* (-6.92 to -3.78) | 350 | -4.85* (-6.47 to -3.23) | 312 | -3.94* (-5.67 to -2.21) | 306 |
| *Improvement PR* | -13.11±9.65 | 237 (67.7) | -13.51±9.88 | 193 (61.9) | -13.83±10.43 | 180 (58.8) |
| *Deterioration PR* | 10.93±10.18 | 113 (32.3) | 8.19±8.92 | 119 (38.1) | 10.19±8.94 | 126 (41.2) |
| *All patients RCP* | -0.52 (-1.77 to +0.73) | 198 | -1.34 (-2.76 to +0.07) | 184 | -0.87 (-2.60 to +0.86) | 174 |
| *Improvement RCP* | -6.61±5.58 | 97 (49.0) | -7.91±5.52 | 75 (40.8) | -7.74±9.51 | 81 (46.6) |
| *Deterioration RCP* | 7.36±5.49 | 101 (51.0) | 7.78±6.18 | 108 (58.7) | 8.46±7.06 | 92 (52.9) |
| *No change RCP* | - | 0 | - | 1 (0.5) | - | 1 (0.6) |

Change was calculated compared with baseline. Negative change represents improvement for CAT, CCQ and SGRQ. Change scores for all patients reported as mean (95%CI). Change scores for improvement and deterioration are presented as mean ± SD.

*Paired t-tests were significant at level $p<0.05$ testing follow-up versus baseline measurements.

*Abbreviations:* 95%CI, 95% Confidence Interval; CAT, COPD Assessment Test; CCQ, Clinical COPD Questionnaire; N, Number of patients; PR, Pulmonary Rehabilitation; RCP, Routine Clinical Practice; SD, Standard Deviation; SGRQ, St. George's Respiratory Questionnaire; T2, Three months follow-up; T3, Six months follow-up; T5, 12 months follow-up.

For peer review only



For peer review only - http://bmjopen.bmj.com/site/about/guidelines.xhtml

### *Clinically Important Improvement versus Deterioration*

Significant correlations between the health status change scores and the GRC ranged respectively for study one -0.33 to -0.41 (CAT), -0.42 to -0.47 (CCQ), and -0.48 to -0.54 (SGRQ). These ranges were for study two respectively -0.29 to -0.37, -0.38 to -0.48, and -0.35 to -0.44. GRC scores had stronger correlations with the respective follow-up health status score in comparison to the baseline score and the computed change scores for both studies.

*Table 3: Correlations between health status (change) scores and the GRC*

| | **GRC T2-T0** | | **GRC T3-T0** | | **GRC T5-T0** | |
|---|---|---|---|---|---|---|
| | *PR (N=355)* | *RCP (N=201)* | *PR (N=319)* | *RCP (N=186)* | *PR (N=309)* | *RCP (N=177)* |
| CAT Change Score | -0.33* | -0.29* | -0.40* | -0.30* | -0.41* | -0.37* |
| CAT T0 | -0.31* | -0.11 | -0.25* | -0.22* | -0.34* | -0.22* |
| CAT T2 | **-0.56*** | -0.31* | **-0.50*** | -0.31* | **-0.50*** | -0.33* |
| CAT T3 | - | - | **-0.55*** | -0.40* | **-0.59*** | -0.34* |
| CAT T5 | - | - | - | - | **-0.64*** | -0.48* |
| | | | | | | |
| CCQ Change Score | -0.42* | -0.38* | -0.44* | -0.40* | -0.47* | -0.48* |
| CCQ T0 | -0.26* | -0.14* | -0.19* | -0.22* | -0.29* | -0.23* |
| CCQ T2 | **-0.61*** | -0.35* | **-0.52*** | -0.26* | **-0.54*** | -0.33* |
| CCQ T3 | - | - | **-0.56*** | -0.43* | **-0.59*** | -0.39* |
| CCQ T5 | - | - | - | - | **-0.66*** | **-0.51*** |
| | | | | | | |
| SGRQ Change Score | -0.48* | -0.35* | **-0.51*** | -0.33* | **-0.54*** | -0.44* |
| SGRQ T0 | -0.28* | -0.13 | -0.24* | -0.20* | -0.32* | -0.22* |
| SGRQ T2 | **-0.62*** | -0.29* | **-0.56*** | -0.25* | **-0.58*** | -0.28* |
| SGRQ T3 | - | - | **-0.61*** | -0.35* | **-0.62*** | -0.35* |
| SGRQ T5 | - | - | - | - | **-0.69*** | **-0.51*** |
| | | | | | | |

Data reported as Pearson or Spearman correlation coefficients between the health status (change) scores and the GRC anchor question. Correlations ≥0.50 are highlighted bold.

* Correlations are significant at level $p < 0.05$.

*Abbreviations:* CAT, COPD Assessment Test; CCQ, Clinical COPD Questionnaire; GRC, Global Rating of Change; N, Number of Patients; PR, Pulmonary Rehabilitation; RCP, Routine Clinical Practice; SGRQ, St. George's Respiratory Questionnaire; T0, Baseline measurement; T2, Three months follow-up; T3, Six months follow-up; T5, 12 months follow-up.

For peer review only

Tables 4-6 and figures 1-3 present the clinically relevant thresholds for minimal, moderate and large changes on the CAT, CCQ and SGRQ during PR and Routine Clinical Practice (RCP). On the CAT anchor- and distribution-based estimates ranged -2.80 to -2.17 (weighted mean -2.51) for minimal improvement and 2.05 to 4.21 for minimal deterioration (weighted mean 2.76) during PR (Table 4, Figure 1). These ranges were respectively -3.78 to -1.53 (weighted mean -2.49) and 1.30 to 1.97 (weighted mean 1.65) during RCP. Weighted thresholds for moderate change were -4.23 for improvement and 7.06 for deterioration during PR. The estimate for moderate deterioration during RCP was 3.89. Clinically relevant large changes are expected at -5.62 for improvement during PR or -4.77 during RCP; and 5.75 for deterioration during RCP.

On the CCQ minimal clinically important improvements were determined at -0.50 to -0.34 (weighted mean -0.40) for PR and -0.44 to -0.19 (weighted mean -0.33) for RCP (Table 5, Figure 2). These thresholds for deterioration were 0.31 to 0.66 (weighted mean 0.43) during PR and 0.19 to 0.46 (weighted mean 0.30) during RCP. Thresholds were -0.82 and -1.05 for respectively moderate and large improvement during PR; 1.23 for moderate deterioration during PR; -1.12 for large improvement during RCP; 0.62 and 0.98 for moderate and large deterioration in RCP.

On the SGRQ estimates ranged -9.20 to -4.83 (weighted mean -6.74) for minimal improvement and 4.46 to 7.52 for minimal deterioration (weighted mean 5.31) during PR (Table 6, Figure 3). These ranges were respectively -4.76 to -2.76 (weighted mean -4.06) and 2.75 to 7.53 (weighted mean 4.78) during RCP. Thresholds were -16.06 and -20.13 for respectively moderate and large improvement during PR; -18.70 for large improvement during RCP; 9.30 for moderate deterioration during PR; and 7.46 for moderate deterioration during RCP.



For peer review only - http://bmjopen.bmj.com/site/about/guidelines.xhtml

*Table 4: Estimates for clinically relevant thresholds for improvement and deterioration on the CAT*

| **CAT** | **T2-T0** | | **T3-T0** | | **T5-T0** | | **Weighted threshold** | |
|---|---|---|---|---|---|---|---|---|
| *Change* | *Improvement* | *Deterioration* | *Improvement* | *Deterioration* | *Improvement* | *Deterioration* | *Improvement* | *Deterioration* |
| | | | | | | | | |
| **Minimal change** | | | | | | | | |
| N Anchor-based PR | 107 | 36 | 96 | 42 | 88 | 43 | 291 | 121 |
| Anchor-based PR | -2.74 | 2.71 | -2.73 | 3.21 | -2.80 | 4.21 | -2.75 | 3.42 |
| N Anchor-based RCP | 12 | 27 | 14 | 36 | 18 | 46 | 32 | 82 |
| Anchor-based RCP | - | - | -2.86 | 1.97 | -3.78 | 1.63 | -3.38 | 1.78 |
| | | | | | | | | |
| N distribution-based PR | 227 | 127 | 184 | 135 | 180 | 129 | 591 | 391 |
| Distribution-based PR | -2.29 | 2.10 | -2.17 | 2.05 | -2.33 | 2.11 | -2.26 | 2.09 |
| N distribution-based RCP | 102 | 83 | 81 | 91 | 79 | 86 | 262 | 260 |
| Distribution-based RCP | -1.67 | 1.83 | -1.53 | 1.44 | -1.58 | 1.30 | -1.60 | 1.52 |
| | | | | | | | | |
| **Moderate change** | | | | | | | | |
| N Anchor-based PR | 51 | 9 | 45 | 7 | 37 | 10 | 133 | 17 |
| Anchor-based PR | -5.02 | - | -3.29 | 8.14 | -4.27 | 6.30 | -4.23 | 7.06 |
| N Anchor-based RCP | 5 | 8 | 12 | 9 | 5 | 9 | - | 9 |
| Anchor-based RCP | - | - | - | - | - | 3.89 | - | 3.89 |
| | | | | | | | | |
| **Large change** | | | | | | | | |
| N Anchor-based PR | 16 | 3 | 12 | 2 | 14 | 3 | 42 | - |
| Anchor-based PR | -4.19 | - | -7.00 | - | -6.07 | - | -5.62 | - |
| N Anchor-based RCP | 4 | 3 | 0 | 2 | 9 | 4 | 13 | 4 |
| Anchor-based RCP | -6.00 | - | - | - | -4.22 | 5.75 | -4.77 | 5.75 |
| | | | | | | | | |
| **No change** | | | | | | | | |
| N Anchor-based PR | 133 | | 115 | | 114 | | 362 | |
| Anchor-based PR | 0.03 | | -0.01 | | -0.33 | | -0.10 | |
| N Anchor-based RCP | 141 | | 113 | | 83 | | 337 | |
| Anchor-based RCP | -0.16 | | -0.54 | | -0.47 | | -0.36 | |

Data reported as clinically relevant threshold or N. Negative change represents improvement for all health status instruments. Paired t-tests were applied with significance level at p <0.05. Non- significant results were excluded, except for the "*No change*" group.

*Abbreviations:* CAT, COPD Assessment Test; GRC, Global Rating of Change; N, Number of Patients; PR, Pulmonary Rehabilitation; RCP, Routine Clinical Practice; T0, Baseline measurement; T2, Three months follow-up; T3, Six months follow-up; T5, 12 months follow-up.

For peer review only



For peer review only - http://bmjopen.bmj.com/site/about/guidelines.xhtml

| **CCQ** | **T2-T0** | | **T3-T0** | | **T5-T0** | | **Weighted threshold** | |
|---|---|---|---|---|---|---|---|---|
| *Change* | *Improvement* | *Deterioration* | *Improvement* | *Deterioration* | *Improvement* | *Deterioration* | *Improvement* | *Deterioration* |
| | | | | | | | | |
| **Minimal change** | | | | | | | | |
| N Anchor-based PR | 107 | 36 | 96 | 42 | 88 | 43 | 291 | 121 |
| Anchor-based PR | -0.44 | 0.42 | -0.42 | 0.48 | -0.50 | 0.66 | -0.45 | 0.53 |
| N Anchor-based RCP | 12 | 27 | 14 | 36 | 18 | 46 | 32 | 82 |
| Anchor-based RCP | - | - | -0.44 | 0.46 | -0.38 | 0.33 | -0.41 | 0.39 |
| | | | | | | | | |
| N distribution-based PR | 225 | 130 | 181 | 138 | 180 | 129 | 586 | 397 |
| Distribution-based PR | -0.36 | 0.34 | -0.34 | 0.34 | -0.36 | 0.31 | -0.35 | 0.33 |
| N distribution-based RCP | 96 | 89 | 87 | 80 | 77 | 88 | 260 | 257 |
| Distribution-based RCP | -0.19 | 0.19 | -0.26 | 0.23 | -0.27 | 0.20 | -0.24 | 0.21 |
| | | | | | | | | |
| **Moderate change** | | | | | | | | |
| N Anchor-based PR | 51 | 9 | 45 | 7 | 37 | 10 | 133 | 7 |
| Anchor-based PR | -0.86 | - | -0.72 | 1.23 | -0.90 | - | -0.82 | 1.23 |
| N Anchor-based RCP | 5 | 8 | 12 | 9 | 5 | 9 | - | 17 |
| Anchor-based RCP | - | 0.85 | - | - | - | 0.42 | - | 0.62 |
| | | | | | | | | |
| **Large change** | | | | | | | | |
| N Anchor-based PR | 16 | 3 | 12 | 2 | 14 | 3 | 42 | - |
| Anchor-based PR | -0.96 | - | -1.03 | - | -1.18 | - | -1.05 | - |
| N Anchor-based RCP | 4 | 3 | 0 | 2 | 9 | 4 | 9 | 4 |
| Anchor-based RCP | - | - | - | - | -1.12 | 0.98 | -1.12 | 0.98 |
| | | | | | | | | |
| **No change** | | | | | | | | |
| N Anchor-based PR | 133 | | 115 | | 114 | | 362 | |
| Anchor-based PR | -0.07 | | 0.17 | | 0.10 | | 0.06 | |
| N Anchor-based RCP | 141 | | 113 | | 83 | | 337 | |
| Anchor-based RCP | -0.03 | | -0.10 | | -0.04 | | -0.06 | |

Data reported as clinically relevant threshold or N. Negative change represents improvement for all health status instruments. Paired t-tests were applied with significance level at p <0.05. Non- significant results were excluded, except for the "*No change*" group.

*Abbreviations:* CCQ, Clinical COPD Questionnaire; GRC, Global Rating of Change; N, Number of Patients; PR, Pulmonary Rehabilitation; RCP, Routine Clinical Practice; T0, Baseline measurement; T2, Three months follow-up; T3, Six months follow-up; T5, 12 months follow-up.

*Table 5: Estimates for clinically relevant thresholds for improvement and deterioration on the CCQ*

For peer review only



For peer review only - http://bmjopen.bmj.com/site/about/guidelines.xhtml

*Table 6: Estimates for clinically relevant thresholds for improvement and deterioration on the SGRQ*

| **SGRQ** | **T2-T0** | | **T3-T0** | | **T5-T0** | | **Weighted threshold** | |
|---|---|---|---|---|---|---|---|---|
| *Change* | *Improvement* | *Deterioration* | *Improvement* | *Deterioration* | *Improvement* | *Deterioration* | *Improvement* | *Deterioration* |
| | | | | | | | | |
| **Minimal change** | | | | | | | | |
| N Anchor-based PR | 107 | 36 | 96 | 42 | 88 | 43 | 291 | 121 |
| Anchor-based PR | -7.58 | 5.01 | -9.20 | 5.14 | -8.82 | 7.52 | -8.49 | 5.95 |
| N Anchor-based RCP | 12 | 27 | 14 | 36 | 18 | 46 | 14 | 82 |
| Anchor-based RCP | - | - | -4.70 | 7.53 | - | 5.60 | -4.70 | 6.45 |
| | | | | | | | | |
| N distribution-based PR | 237 | 113 | 193 | 119 | 180 | 126 | 610 | 358 |
| Distribution-based PR | -4.83 | 5.09 | -4.94 | 4.46 | -5.22 | 4.47 | -4.98 | 4.66 |
| N distribution-based RCP | 97 | 101 | 75 | 108 | 81 | 92 | 253 | 301 |
| Distribution-based RCP | -2.79 | 2.75 | -2.76 | 3.09 | -4.76 | 3.53 | -3.41 | 3.11 |
| | | | | | | | | |
| **Moderate change** | | | | | | | | |
| N Anchor-based PR | 51 | 9 | 45 | 7 | 37 | 10 | 124 | 10 |
| Anchor-based PR | -15.85 | - | -13.63 | - | -15.40 | 9.30 | -16.06 | 9.30 |
| N Anchor-based RCP | 5 | 8 | 12 | 9 | 5 | 9 | - | 9 |
| Anchor-based RCP | - | - | - | - | - | 7.46 | - | 7.46 |
| | | | | | | | | |
| **Large change** | | | | | | | | |
| N Anchor-based PR | 16 | 3 | 12 | 2 | 14 | 3 | 42 | - |
| Anchor-based PR | -18.33 | - | -21.99 | | -20.58 | - | -20.13 | - |
| N Anchor-based RCP | 4 | 3 | 0 | 2 | 9 | 4 | 9 | - |
| Anchor-based RCP | - | - | - | - | -18.70 | - | -18.70 | - |
| | | | | | | | | |
| **No change** | | | | | | | | |
| N Anchor-based PR | 133 | | 115 | | 114 | | 362 | |
| Anchor-based PR | -1.50 | | -0.99 | | -0.06 | | -0.88 | |
| N Anchor-based RCP | 141 | | 113 | | 83 | | 337 | |
| Anchor-based RCP | 0.51 | | 0.19 | | 0.10 | | 0.30 | |

Data reported as clinically relevant threshold or N. Negative change represents improvement for all health status instruments. Paired t-tests were applied with significance level at $p < 0.05$. Non- significant results were excluded, except for the "*No change*" group.

*Abbreviations:* GRC, Global Rating of Change; N, Number of Patients; PR, Pulmonary Rehabilitation; RCP, Routine Clinical Practice; SGRQ, St. George's Respiratory Questionnaire; T0, Baseline measurement; T2, Three months follow-up; T3, Six months follow-up; T5, 12 months follow-up.

*Figure 1: Forrest plot of clinically relevant thresholds for improvement and deterioration on the CAT.*

*Figure 2: Forrest plot of clinically relevant thresholds for improvement and deterioration on the CCQ.*

*Figure 3: Forrest plot of clinically relevant thresholds for improvement and deterioration on the SGRQ.*

*Legend Figures 1-3: Data are presented as mean estimates (squares) including 95% confidence interval (horizontal lines). Estimates from the half standard deviation analysis are represented as single squares. Weighted mean estimates are presented as larger diamonds. Data are separated as minor, moderate and large improvement thresholds (left half), versus minor and moderate deterioration thresholds (right half).*

For peer review only



For peer review only - http://bmjopen.bmj.com/site/about/guidelines.xhtml

## Discussion

*Summary of main findings*

Using both anchor- and distribution-based methods, the *weighted MCIDs* for improvement and deterioration on the CAT were respectively -2.51 vs. 2.76 during PR; and -2.49 vs. 1.65 during Routine Clinical Practice (RCP). These thresholds for improvement and deterioration on the CCQ were respectively -0.40 vs. 0.43 during PR; and -0.33 vs. 0.30 during RCP. MCIDs were respectively -6.74 vs. 5.31 during PR; and -4.06 vs. 4.78 during RCP for improvement and deterioration on the SGRQ. Estimates for minimal clinically important improvement and deterioration were overall somewhat similar, however absolute MCIDs differed between PR and RCP. Thresholds for *moderate* and *large* improvement and deterioration differed from each other, as well as between study settings.

*Interpretation of findings*

Little evidence exists whether MCIDs for improvement are similar for deterioration [20, 22, 36]. Jaeschke et al. were the first to determine the MCID of a health status tool using a 15-point GRC combining both improved and deteriorated COPD patients into one group of minimally changed participants [19]. Juniper et al. elaborated on this by separating minimally improved patients from deterioration in asthma, but only a limited number of patients indicated deterioration and no conclusions upon the MCID of deterioration were drawn [33]. Outside the field of COPD, Crosby et al. and de Vet et al. stated that some studies demonstrated that a smaller MCID for improvement was required compared with deterioration [20, 36]. The current study does not confirm this; although MCIDs seemed smaller for RCP patients compared with PR. In general, the absolute values for the MCIDs for improvement and deterioration did not seem to differ much here, with the exception of the SGRQ during PR.

The ranges found in this study for the MCID of the CAT *(improvement -3.78 to -1.53; deterioration 1.30 to 4.21)* matched with estimates found in other studies [11-15]. Two studies used a patient-assessed GRC to estimate the MCID of the CAT [14-15]. However, no results were reported for worsened patients or the numbers of patients were too few. Other anchor-based methods suggested that a change of one point on the CAT might represent the MCID for deterioration [14]. The thresholds for minimal clinically relevant improvement *(-2.51 in PR and -2.49 in RCP)* seemed somewhat comparable with the ones for deterioration *(2.76 in PR and 1.65 in RCP)* in the current study, except for deterioration during routine clinical practice. As CAT allows only integer scores [2], a change of three points seems a valid threshold for improvement and deterioration, although the



For peer review only - http://bmjopen.bmj.com/site/about/guidelines.xhtml

MCID for deterioration in RCP should be closer to two points. Thresholds for moderate improvement *(-4.23 in PR)* and deterioration *(7.06 in PR and 3.89 in RCP)* turned out less similar. The number of patients moderately deteriorating was low and differences were observed between both study settings. Moderate change might be experienced with a change on the CAT of 4-7 points. Two previous studies suggested that a cut-off point of four points was identified for acute HRQoL deterioration in clinical practice [37-38]. This would match our estimates for moderate change. The number of patients with a large change was too low leading to wide confidence intervals for valid conclusions.

Regarding the CCQ, the MCID ranges found for both improvement *(-0.50 to -0.19)* and deterioration *(0.19 to 0.66)* overlapped each other in absolute sense, indicating that estimates for improvement and deterioration may be similar. However, differences were noted between PR *(±0.40)* and RCP *(±0.30)* for both minimal improvement and deterioration. These estimates for the MCID matched with earlier evidence [8-13]. One other study used a GRC to determine the MCID of the CCQ [8]. Unfortunately, no data were available on worsening patients. Thresholds for moderate change on the CCQ were broad (±0.62 to ±1.23). Few patients experienced large changes, but estimates for both types of MCID from both study settings were approximately one point.

Minimal thresholds for improvement *(-9.20 to -2.76)* and deterioration *(2.75 to 7.53)* on the SGRQ overlapped each other, although more variation was present here. A change of approximately four to seven points for both improvement and deterioration seemed to be the minimal clinically important threshold in the current study. The MCID for improvement during PR *(-6.74)* was larger than for deterioration *(5.31)*; however, confidence intervals for deterioration were wide. Estimates for the thresholds during RCP (four to five points) were smaller compared with PR (five to seven points). Moreover, the distribution-based estimates turned out smaller than the anchor-based estimates, lowering the absolute MCIDs. Thresholds for moderate improvement and deterioration in the current study were not very similar ranging absolutely from 7.46 to 16.06 points. Estimates for clinically relevant large HRQoL improvement on the SGRQ ranged -20 to -18 points for PR and RC, but too few patients were included to draw valid conclusions.

The SGRQ MCID matched to some extent with previous results [12, 16-18]. Jones et al. published a threshold of four points, which is generally accepted and applied in clinical practice [16, 18]. Interestingly, most results in our current study suggest a larger MCID, although estimates from RCP included this four point's estimate. The estimate by Jones et al. was based upon a study using patient preference-based techniques in COPD by applying a five-point patients' judgement of treatment efficacy (Salmeterol). This MCID of four points was valid for the



For peer review only - http://bmjopen.bmj.com/site/about/guidelines.xhtml

group of patients that experienced effective treatment. In addition, a clinicians' five-point GRC was scored, resulting in a MCID of four points. Clinicians' and patients' ratings are however not necessarily similar [39].

*Strengths and limitations of current study*

This study was the first to investigate clinically relevant thresholds for minimal, moderate and large changes in COPD health status comparing both improvement and deterioration using a triangulation of both anchor- and distribution-based methods. There were sufficient correlations between the GRC and respective health status questionnaires as required [21]; although they were still only weak to moderate. It should be noted that correlations were stronger with the follow-up score compared with the baseline and/or change score, possibly due to a response shift. Another strength is that multiple follow-up measurement periods were included to limit possible influence of the period of measurements on the MCID [20, 23]. Moreover, this study investigated clinically relevant thresholds for both PR and a routine clinical practice, improving its clinical application and external validity. Although this is the first study to investigate thresholds for clinically relevant deterioration, still a limited number of patients indicated deterioration in HRQoL after PR and during routine clinical practice. A second limitation is that the found thresholds demonstrate wide confidence intervals, limiting its accuracy and requiring an even larger sample size than our current studies.

*Implications for future research and clinical practice*

COPD patients tend to have worsening HRQoL over time; hence MCIDs for deterioration have an important implication for clinical practice [40-41]. Clinicians and researchers should be able to judge whether patients were really worsening over time or that change observed was random fluctuation. Preventing clinically relevant deterioration in HRQoL by means of therapy is thus an important goal for the physician too. Ideally, more research is needed to validate our thresholds for clinically relevant deterioration on the CAT, CCQ and SGRQ. One cannot directly transform the thresholds for improvement into those for deterioration, as it remains unclear whether they are similar. Evidence outside the field of COPD has found differences. However, in the current study, the estimates turned out rather similar with differing MCIDs between studies. Setting could thus potentially impact the MCID.

*Conclusions*

Determining deterioration in HRQoL is of importance, since one needs to differentiate between real worsening of a patient's status and random variations. In this study, estimates for clinically relevant thresholds for



For peer review only - http://bmjopen.bmj.com/site/about/guidelines.xhtml

improvement and deterioration were somewhat similar, but differed between settings. We would recommend using cut-points of CAT≥3 (intervention), CAT≥2 (RCP), CCQ ≥0.40 (intervention), CCQ≥0.30 (RCP), SGRQ≥6 (intervention) and SGRQ ≥5 (RCP) for both *minimal* improvement and deterioration. Thresholds for respectively *moderate* and *large* changes should be explored, but could approximately be in the range of 4-5 and 5-6 for CAT; 0.80 and 1.00 for CCQ; 10-15 points and 15-20 points for SGRQ.

For peer review only



For peer review only - http://bmjopen.bmj.com/site/about/guidelines.xhtml

## List of Abbreviations

| | |
|---|---|
| 0.5SD | Half Standard Deviation |
| 95%CI | 95% Confidence Interval |
| CAT | COPD Assessment Test |
| CCQ | Clinical COPD Questionnaire |
| COPD | Chronic Obstructive Pulmonary Disease |
| FEV1%Pred | Forced Expiratory Volume in one second % predicted |
| GOLD | Global initiative for Obstructive Lung Diseases |
| GRC | Global Rating of Change scale |
| HRQoL | Health-Related Quality of Life |
| MCID | Minimal Clinically Important Difference |
| N | Number of Patients |
| PR | Pulmonary Rehabilitation |
| PROs | Patient-Reported Outcomes |
| RCP | Routine Clinical Practice |
| RIMTCORE | Routine Inspiratory Muscle Training within COPD Rehabilitation |
| SD | Standard Deviation |
| SGRQ | St. George Respiratory Questionnaire |
| T0 | Baseline PR measurement |
| T1 | Time point 1: 3-weeks PR discharge |
| T2 | Time point 2: 3 months follow-up |
| T3 | Time point 3: 6 months follow-up |
| T4 | Time point 4: 9 months follow-up |
| T5 | Time point 5: 12 months follow-up |
| UMCG | University Medical Center Groningen |

## Appendices

Figures 1-3

For peer review only

## References


[1] Global Initiative for Chronic Obstructive Lung Disease (GOLD). Global Strategy for the Diagnosis, Management and Prevention of COPD (2017 Report). 2017; Available at http://www.goldcopd.org/gold-2017-global-strategy-diagnosis-management-prevention-copd/. Accessed 17th of July 2017.

[2] Jones, PW et al. Development and first validation of the COPD Assessment Test. Eur. Respir. J. 2009; 34: 648-654.

[3] Van der Molen, T et al. Development, validity and responsiveness of the Clinical COPD Questionnaire. Health Qual. Life Outcomes 2003; 1: 13.

[4] Jones, PW, Quirk, FH and Baveystock, CM. The St George's Respiratory Questionnaire. Respir. Med. 1991; 85: 25-31.

[5] Cave, AJ et al. Assessment of COPD wellness tools for use in primary care: an IPCRG initiative. Int. J. of COPD 2012; 7: 447-456.

[6] Lozano, R et al. Global and regional mortality from 235 causes of death for 20 age groups in 1990 and 2010: a systematic analysis for the Global Burden of Disease Study 2010. Lancet 2012; 380: 2095-2128.

[7] Adelove, D et al. Global and regional estimates of COPD prevalence: Systematic review and meta-analysis. J. Glob. Health 2015; 5(2): 020415.

[8] Kocks, JW et al. Health status measurement in COPD: the minimal clinically important difference of the clinical COPD questionnaire. Respir. Res. 2006; 7: 62.

[9] Kon, SS et al. The Clinical COPD Questionnaire: response to pulmonary rehabilitation and minimal clinically important difference. Thorax 2013; 69: 793-798.

[10] Canavan, JL et al. Clinical COPD Questionnaire in patients with chronic respiratory disease. Respirology 2014; 19: 1006-1012.

[11] Tsiligianni, IG et al. Assessing health status in COPD. A head-to-head comparison between the COPD assessment test (CAT) and the clinical COPD questionnaire (CCQ). BMC Pulm. Med. 2012; 12: 20.

[12] Alma, HJ et al. Health status instruments for patients with COPD in pulmonary rehabilitation: defining a minimal clinically important difference. Npj PCRM 2016; 26: 16041.

[13] Smid, DE et al. Responsiveness and MCID Estimates for CAT, CCQ and HADS in Patients With COPD Undergoing Pulmonary Rehabilitation: A Prospective Analysis. JAMADA 2017; 18(1): 53-58.

[14] Kon, SS et al. Minimum clinically important difference for the COPD Assessment Test: a prospective analysis. Lancet Respir. Med. 2014; 2: 195-203.

[15] Dodd, JW et al. The COPD assessment test (CAT): response to pulmonary rehabilitation. A multicentre, prospective study. Thorax 2011; 66: 425-429.

[16] Jones, PW. St. George's Respiratory Questionnaire: MCID. COPD 2005; 2: 75-79.

[17] Welling, JB et al. The minimal important difference for the St George's Respiratory Questionnaire in patients with severe COPD. Eur. Respir. J. 2015; 46: 1598.

[18] Jones, PW and Bosh, TK. Quality of life changes in COPD patients treated with salmeterol. Am. J. Respir. Crit. Care Med. 1997; 155: 1283-1289.




For peer review only - http://bmjopen.bmj.com/site/about/guidelines.xhtml

[19] Jaeschke, R, Singer, J and Guyatt, GH. Measurement of health status. Ascertaining the minimal clinically important difference. Control Clin. Trials 1989, 10: 407-415.

[20] Crosby, RD, Kolotkin, RL and Williams, GR. Defining clinically meaningful change in health-related quality of life. Journal of Clinical Epidemiology 2003; 56: 395-400.

[21] Revicki, D et al. Recommended methods for determining responsiveness and minimally important differences for patient-reported outcomes. J. Clin. Epidemiol. 2008; 61: 102-109.

[22] Beaton, DE, Boers, M and Wells, GA. Many faces of the minimal clinically important difference (MCID): a literature review and directions for future research. Curr. Opin. Rheumatol. 2002; 14: 109-114.

[23] Hays, RD and Woolley, M. The Concept of Clinically Meaningful Difference in Health-Related Quality of-Life Research How Meaningful is it? Pharmacoeconomics 2000; 18(5): 419-423.

[24] Singh, D et al. Reduction in clinically important deterioration in chronic obstructive pulmonary disease with aclidinium/formoterol. Respiratory Research 2017; 18:106.

[25] Raman, S et al. Minimal clinically important differences in the EORTC QLQ-BM22 and EORTC QLQ-C15-PAL modules in patients with bone metastases undergoing palliative radiotherapy. Qual Life Res. 2016; 25(10): 2535-2541.

[26] Maringwa, J et al. Minimal clinically meaningful differences for the EORTC QLQ-C30 and EORTC QLQ-BN20 scales in brain cancer patients. Ann Oncol. 2011; 22(9): 2107-2112.

[27] Hui, D et al. Minimal Clinically Important Difference in the Physical, Emotional, and Total Symptom Distress Scores of the Edmonton Symptom Assessment System. J Pain Symptom Manage 2016; 51(2): 262-269.

[28] Tuomi, L et al. Interpretation of the Swedish Self Evaluation of Communication Experiences after Laryngeal cancer: Cutoff levels and minimum clinically important differences. Head Neck 2016; 38(5): 689-695.

[29] Rouquette, A et al. The minimal clinically important difference determined using item response theory models: an attempt to solve the issue of the association with baseline score. J Clin Epidemiol 2014; 67(4): 433-440.

[30] Hui, D et al. Minimal clinically important differences in the Edmonton Symptom Assessment Scale in cancer patients: A prospective, multicentre study. Cancer 2015; 121(17): 3027-3035.

[31] Schultz, K et al. Inspiratory Muscle Training does not improve clinical outcomes in 3-week COPD rehabilitation: Results from a Randomised Controlled Trial. European Respiratory Journal 2018; 51: 1702000.

[32] Kamper, SJ, Maher, CG and Mackay, G. Global rating of change scales: a review of strengths and weaknesses and considerations for design. J. Man. Manip. Ther. 2009; 17: 163-170.

[33] Juniper, EF et al. Determining a minimal important change in a disease-specific quality of life questionnaire. J. Clin Epidemiol 1994; 47(1): 81-87.

[34] Norman, GR, Sloan, JA and Wyrwich, KW. Interpretation of changes in health-related quality of life: the remarkable universality of half a standard deviation. Med. Care 2003; 41: 582-592.

[35] Terwee, CB et al. Quality criteria were proposed for measurement properties of health status questionnaires. J Clin Epidemiol 2007; 60: 34-42.

[36] de Vet, HC et al. Minimal changes in health status questionnaires: distinction between minimally detectable change and minimally important change. Health and Quality of Life Outcome 2006; 4:54.

[37] Pothirat, C et al. Detection of acute deterioration in health status visit among COPD patients by monitoring COPD assessment test score. International Journal of COPD 2015; 10: 277-282.



For peer review only - http://bmjopen.bmj.com/site/about/guidelines.xhtml

[38] García-Sidro, P et al. The CAT (COPD Assessment Test) questionnaire as a predictor of the evolution of severe COPD exacerbations. Respir Med. 2015; 109(12): 1546-1552.

[39] Janse, AJ et al. Quality of life: patients and doctors don't always agree: a meta-analysis. J Clin Epidemiol. 2004; 57(7): 653-661.

[40] Yoo, JY et al. Factors affecting the trajectory of health-related quality of life in COPD patients. Int J Tuberc Lung Dis 2016; 20(6): 738-746.

[41] Sundh, J et al. Change in health status in COPD: a seven-year follow-up cohort study. npj Primary Care Respiratory Medicine 2016; 26: 16073.

For peer review only



For peer review only - http://bmjopen.bmj.com/site/about/guidelines.xhtml

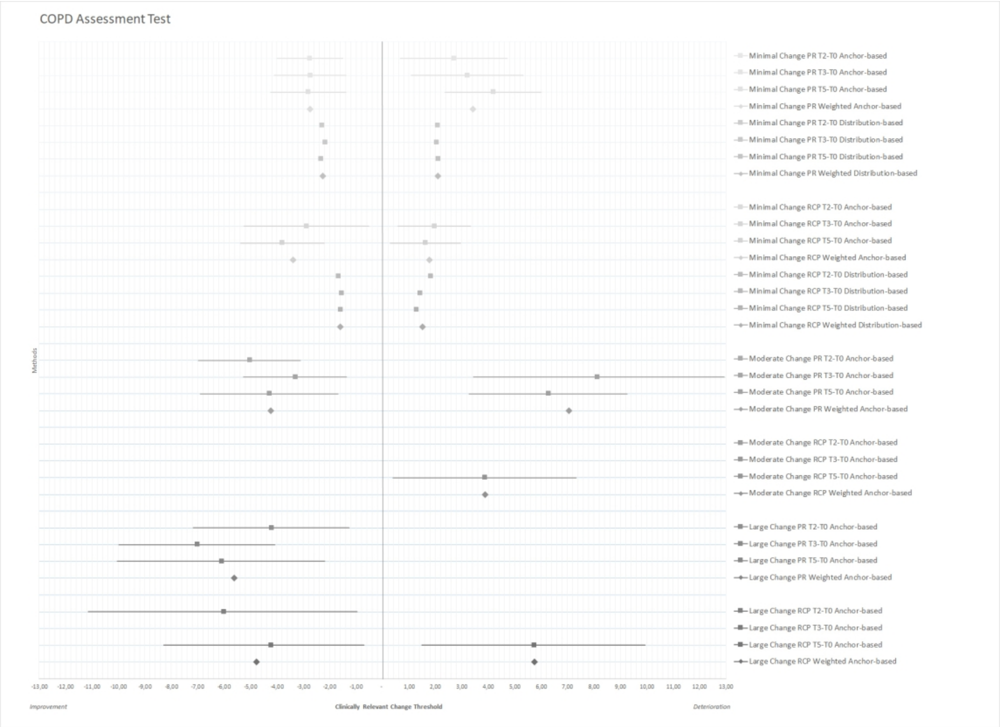


Caption: Figure 1: Forrest plot of clinically relevant thresholds for improvement and deterioration on the CAT.

Legend: Data are presented as mean estimates (squares) including 95% confidence interval (horizontal lines). Estimates from the half standard deviation analysis are represented as single squares. Weighted mean estimates are presented as larger diamonds. Data are separated as minor, moderate and large improvement thresholds (left half), versus minor and moderate deterioration thresholds (right half).

159x115mm (300 x 300 DPI)

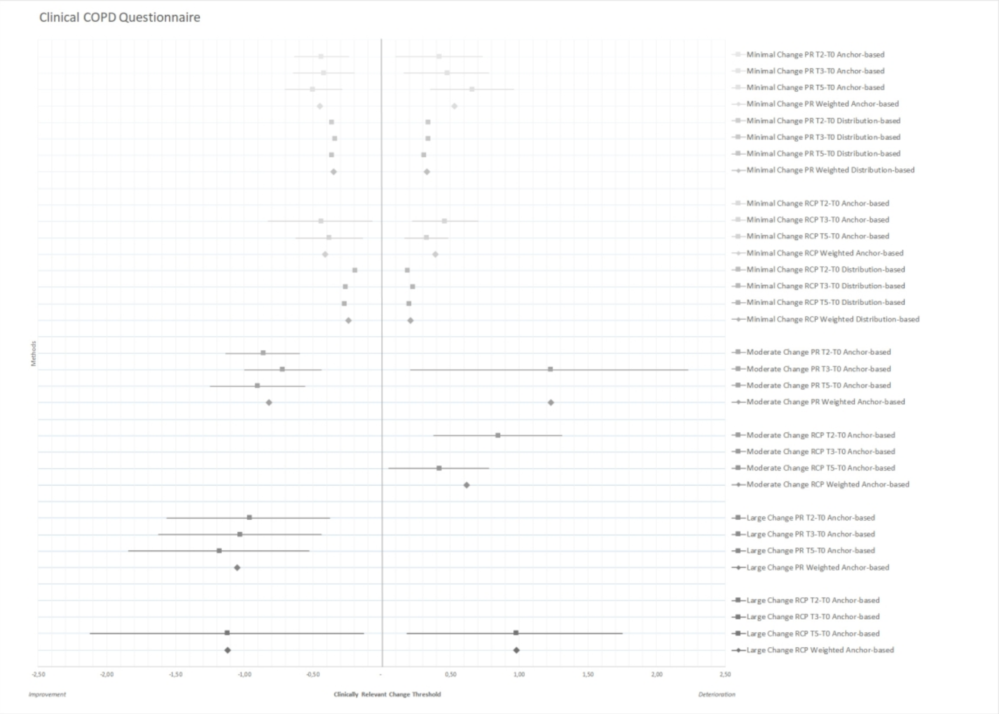


Caption: Figure 2: Forrest plot of clinically relevant thresholds for improvement and deterioration on the CCQ.

Legend: Data are presented as mean estimates (squares) including 95% confidence interval (horizontal lines). Estimates from the half standard deviation analysis are represented as single squares. Weighted mean estimates are presented as larger diamonds. Data are separated as minor, moderate and large improvement thresholds (left half), versus minor and moderate deterioration thresholds (right half).

159x114mm (300 x 300 DPI)

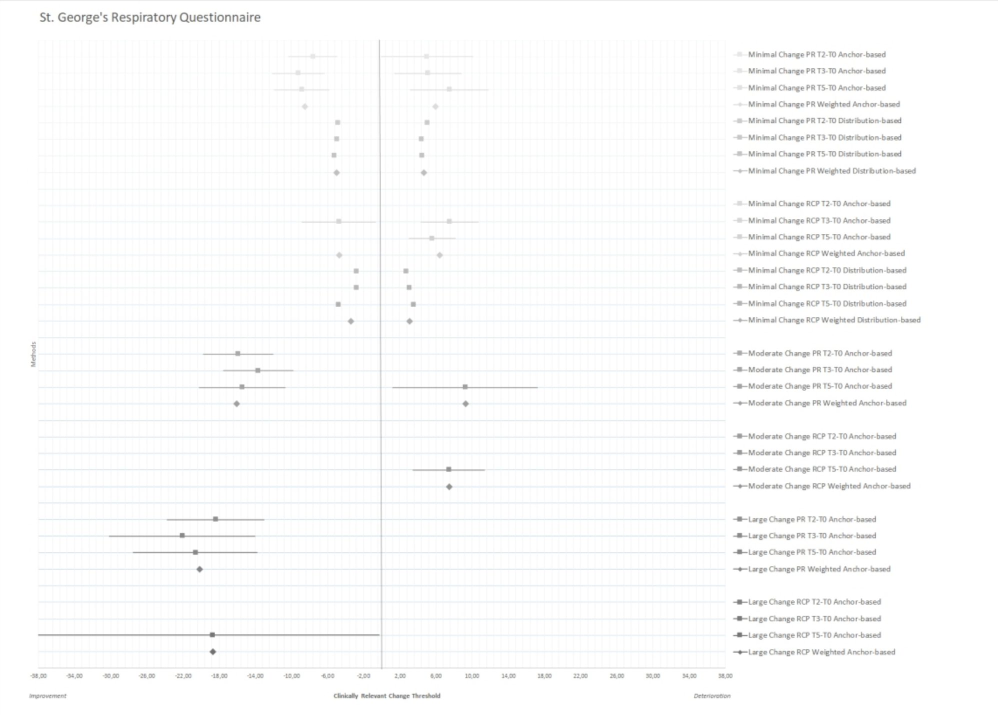


Caption: Figure 3: Forrest plot of clinically relevant thresholds for improvement and deterioration on the SGRQ.

Legend: Data are presented as mean estimates (squares) including 95% confidence interval (horizontal lines). Estimates from the half standard deviation analysis are represented as single squares. Weighted mean estimates are presented as larger diamonds. Data are separated as minor, moderate and large improvement thresholds (left half), versus minor and moderate deterioration thresholds (right half).

160x114mm (300 x 300 DPI)

BMJ Open

# Thresholds for Clinically Important Deterioration versus Improvement in COPD Health Status during Pulmonary Rehabilitation and Routine Clinical Practice: Results from Prospective Research

| | |
|---|---|
| Journal: | *BMJ Open* |
| Manuscript ID | bmjopen-2018-025776.R1 |
| Article Type: | Research |
| Date Submitted by the Author: | 22-Feb-2019 |
| Complete List of Authors: | Alma, Harma; University of Groningen, University Medical Center Groningen, Department of General Practice and Elderly Care Medicine; University of Groningen, University Medical Center Groningen, Groningen Research Institute for Asthma and COPD (GRIAC)<br>de Jong, Corina; University of Groningen, University Medical Center Groningen, Department of General Practice and Elderly Care Medicine; University of Groningen, University Medical Center Groningen, Groningen Research Institute for Asthma and COPD (GRIAC)<br>Jelusic, Danijel; Klinik Bad Reichenhall, Center for Rehabilitation, Pulmonology and Orthopedics<br>Wittmann, Michael; Klinik Bad Reichenhall, Center for Rehabilitation, Pulmonology and Orthopedics<br>Schuler, Michael; Julius-Maximilians-Universitat Wurzburg, Medical Psychology and Psychotherapy, Medical Sociology and Rehabilitation Sciences<br>Sanderman, Robbert; University of Groningen, University Medical Center Groningen, Department of Health Psychology; University of Twente, Department of Psychology, Health and Technology<br>Schultz, Konrad; Klinik Bad Reichenhall, Center for Rehabilitation, Pulmonology and Orthopedics<br>Kocks, Janwillem; University of Groningen, University Medical Center Groningen, Department of General Practice and Elderly Care Medicine; University of Groningen, University Medical Center Groningen, Groningen Research Institute for Asthma and COPD (GRIAC)<br>van der Molen, Thys; University of Groningen, University Medical Center Groningen, Department of General Practice and Elderly Care Medicine; University of Groningen, University Medical Center Groningen, Groningen Research Institute for Asthma and COPD (GRIAC) |
| <b>Primary Subject Heading</b>: | Respiratory medicine |
| Secondary Subject Heading: | Epidemiology, Research methods, Respiratory medicine |
| Keywords: | Chronic Obstructive Pulmonary Disease (COPD), Health-Related Quality of Life (HRQoL), Health Status Responsiveness, Pulmonary Rehabilitation (PR), Routine Clinical Practice (RCP), Minimal Clinically Important Difference (MCID) |

# Thresholds for Clinically Important Deterioration versus Improvement in COPD Health Status during Pulmonary Rehabilitation and Routine Clinical Practice: Results from Prospective Research

Harma Alma[ab], MD MSc; Corina de Jong, PhD[ab]; Danijel Jelusic[c], MD; Michael Wittmann[c], MD; Michael Schuler[d], PhD; Prof. dr. Robbert Sanderman[ef], PhD; Konrad Schultz, MD[c]; Janwillem Kocks[ab], MD, PhD; Prof. dr. Thys van der Molen[ab], MD, PhD

[a] University of Groningen, University Medical Center Groningen, Department of General Practice and Elderly Care Medicine, Groningen, The Netherlands

[b] University of Groningen, University Medical Center Groningen, Groningen Research Institute for Asthma and COPD (GRIAC), Groningen, The Netherlands

[c] Klinik Bad Reichenhall, Center for Rehabilitation, Pulmonology and Orthopedics, Bad Reichenhall, Germany

[d] University of Wuerzburg, Department of Medical Psychology and Psychotherapy, Medical Sociology and Rehabilitation Sciences, Wuerzburg, Germany

[e] University of Groningen, University Medical Center Groningen, Department of Health Psychology, Groningen, The Netherlands

[f] University of Twente, Department of Psychology, Health and Technology, Enschede, The Netherlands

| | |
|---|---|
| Harma Alma | h.j.alma@umcg.nl |
| Corina de Jong | c.de.jong02@umcg.nl |
| Danijel Jelusic | djelusic87@gmail.com |
| Michael Wittmann | michael.wittmann@klinik-bad-reichenhall.de |
| Michael Schuler | m.schuler@uni-wuerzburg.de |
| Robbert Sanderman | r.sanderman@umcg.nl |
| Konrad Schultz | konrad.schultz@klinik-bad-reichenhall.de |
| Janwillem Kocks | j.w.h.kocks@umcg.nl |
| Thys van der Molen | t.van.der.molen@umcg.nl |

Corresponding author:

Mrs. H.J. Alma, MD MSc
University of Groningen, University Medical Center Groningen
Department of General Practice and Elderly Care Medicine
HPC FA21, Postbox 196
NL-9700 AD Groningen
T: +31 6 27137024
E: h.j.alma@umcg.nl

Total word count manuscript: 3945 words (Abstract 367 words)



For peer review only - http://bmjopen.bmj.com/site/about/guidelines.xhtml

## Abstract

**Introduction:**

COPD is a progressive chronic disease, implying that preventing deterioration of health status is an important therapy goal. (Minimal) Clinically Important Differences ((M)CIDs) are currently used to interpret changes observed. Until now, it remains unclear whether (M)CIDs are similar for both deterioration and improvement in health status. This study investigated clinically relevant thresholds for deterioration versus improvement for three widely used health status questionnaires in COPD in two settings.

**Methods:**

Data were retrospectively analysed from two prospective studies. In study one, COPD patients GOLD II-IV aged ≥18 years without respiratory co-morbidities were recruited during an in-house 3-week Pulmonary Rehabilitation (PR) randomized controlled trial in the Klinik Bad Reichenhall in Germany. In study two, GOLD I-IV patients aged ≥40 years without respiratory co-morbidities were recruited from Dutch primary and secondary Routine Clinical Practice (RCP) via general practitioners, pulmonary physicians and the patient lung federation. The COPD Assessment Test (CAT), Clinical COPD Questionnaire (CCQ) and St. George's Respiratory Questionnaire (SGRQ) were completed at baseline, three, six, and 12 months. A 15-point Global Rating of Change scale (GRC) was added at each follow-up to retrospectively assess change in health status. Anchor-based- (GRC) and distribution-based (half Standard Deviation) methods were used to determine clinically relevant thresholds.

**Results:**

In total, 451 patients were included from PR (57.87±6.56 years, 65% male, 50/39/11% GOLD II/III/IV) and 207 patients from RCP (66.69±7.91, 58.5% male and 17/40/30/12% GOLD I/II/III/IV). MCIDs for deterioration ranged 1.30 to 4.21 (CAT), 0.19 to 0.66 (CCQ), and 2.75 to 7.53 (SGRQ). MCIDs for improvement ranged -3.78 to -1.53 (CAT), -0.50 to -0.19 (CCQ), and -9.20 to -2.76 (SGRQ). Thresholds for moderate improvement versus deterioration ranged -5.02 to -3.29 vs. 3.89 to 8.14 (CAT), -0.90 to -0.72 vs. 0.42 to 1.23 (CCQ), and -15.85 to -13.63 vs. 7.46 to 9.30 (SGRQ).

**Conclusions:**

MCID ranges for improvement and deterioration on the CAT, CCQ and SGRQ were somewhat similar. However, estimates for moderate and large change varied and were inconsistent. Thresholds differed between study settings.




For peer review only - http://bmjopen.bmj.com/site/about/guidelines.xhtml

**Trial registration number:**

PR patients were recruited from the RIMTCORE trial (#DRKS00004609 and #12107 Ethik-Kommission der Bayerischen Landesärztekammer). Dutch RCP patients were recruited from the MCID study registered at the University Medical Center Groningen (UMCG) Research Register (#201500447).

For peer review only



For peer review only - http://bmjopen.bmj.com/site/about/guidelines.xhtml

## Article Summary

**Strengths:**

- Our study is the first dedicated investigation of (Minimal) Clinically Important Differences ((M)CIDs) for deterioration on COPD health status tools in comparison to those for improvement.
- Our study used a combination of anchor- and distribution-based methods to determine clinically relevant thresholds for both deterioration and improvement.
- Our study investigated clinically relevant thresholds in two different study settings - Pulmonary Rehabilitation (PR) and Routine Clinical Practice (RCP) - by using data from various follow-up periods to minimize the possible impact of the recall period.

**Limitations:**

- Our study included a limited number of patients with deterioration after PR intervention and during RCP and a limited number of patients indicating moderate and large changes in health status.
- Our study resulted in broad ranges and wide confidence intervals for the (M)CIDs of COPD health status tools, requiring possibly larger sample sizes for more accuracy.

For peer review only

## Declarations

## Funding

The main RIMTCORE trial (#DRKS00004609), including patients in Pulmonary Rehabilitation (PR), was funded by the *Deutsche Rentenversicherung*. The Dutch observational study on COPD health status in Routine Clinical Practice (RCP) as well as the current combined retrospective analysis of both prospective studies received financial support from the Junior Scientific Masterclass (JSM) as part of the University of Groningen.

## Competing interests

H.J. Alma, C. de Jong, D. Jelusic, M. Wittmann, M. Schuler and R. Sanderman have nothing to disclose. J.W.H. Kocks reports personal fees from Novartis; research grants and personal fees from Boehringer Ingelheim; research grants and personal fees from GSK; research grants from Stichting Zorgdraad; personal fees from IPCRG; personal fees from Springer Media; and travel arrangements from Chiesi BV, GlaxoSmithKline BV, and IPCRG, all outside the submitted work. K. Schultz received lecture fees from Boehringer, AstraZeneca, Berlin Chemie, Novartis, Chiesi, Mundipharma, Takeda, GSK and MSD, all outside the submitted work. T. van der Molen reports personal reimbursements from GSK, TEVA, Astra Zeneca, Boehringer Ingelheim and study grants from Astra Zeneca and GSK. After this study was terminated, he became employee of GSK. None of these stated conflicts of interest are linked to the current manuscript. T. van der Molen developed the CCQ and holds the copyright.

## Authors' contributions

KS, MW, DJ and MS planned the RIMTCORE study design and were responsible for data collection. HA, CdJ, RS and TvdM designed the Dutch observational study on COPD health status in Routine Clinical Practice as well as the current retrospective analysis of both prospective studies. HA and CdJ performed the statistical analysis. HA wrote the first draft, while CdJ, JK, RS and TvdM actively participated in the review process. RS and TvdM supervised and participated in different steps of the study, as well as in writing. All authors participated in and approved of the final version of the manuscript

## Consent of publication

All authors participated in various steps in the study, edited the manuscript and gave their approval for submission.



For peer review only - http://bmjopen.bmj.com/site/about/guidelines.xhtml

## Ethics approval and consent to participate

This study is a secondary retrospective analysis of a subsample from the Routine Inspiratory Muscle Training within COPD Rehabilitation (RIMTCORE) real-life randomized controlled trial (#DRKS00004609) in the Klinik Bad Reichenhall, Center for Rehabilitation, Pulmonology and Orthopaedics in Germany; and a primary analysis of all patients participating in the Dutch observational trial (MCID study) on COPD health status in routine clinical practice (UMCG trial #201500447). All patients in both studies signed informed consent upon participation. The RIMTCORE trial has been approved by the Ethik-Kommission der Bayerischen Landesärztekammer and registered in the German Clinical Trial Register. The MCID study has been registered at the University Medical Center Groningen (UMCG) Research Register and evaluated by its Medical Ethical Committee.

## Data Sharing Statement

The data that support the findings of this study are not publicly available. Participating patients in the RIMTCORE trial have only agreed upon availability of their data to the Klinik Bad Reichenhall, their scientific partners in the data analysis and the Committee of the Bavarian State Chamber of Labor in Munich. Participating patients in the MCID study only agreed upon availability of their data to the University Medical Center Groningen (UMCG) and their scientific partners in the data analysis.

## Acknowledgements

We are grateful to the Junior Scientific Masterclass of the University of Groningen, who financially supported the research position of the first author. We would also like to acknowledge all participating patients in both the RIMTCORE trial and the MCID study.

For peer review only



For peer review only - http://bmjopen.bmj.com/site/about/guidelines.xhtml

**Key Words**

Chronic Obstructive Pulmonary Disease (COPD)

Health-Related Quality of Life (HRQoL)

Health Status Responsiveness

Pulmonary Rehabilitation (PR)

Routine Clinical Practice (RCP)

Minimal Clinically Important Difference (MCID)

For peer review only



For peer review only - http://bmjopen.bmj.com/site/about/guidelines.xhtml

# Article manuscript

## Introduction

The use of health status questionnaires is recommended by the Global Initiative for Chronic Obstructive Lung Disease (GOLD) for the assessment, evaluation and management of patients with Chronic Obstructive Pulmonary Disease (COPD) [1]. The COPD Assessment Test (CAT) [2], the Clinical COPD Questionnaire (CCQ) [3], and the St. George's Respiratory Questionnaire (SGRQ) [4] are frequently used patient-reported health status tools important for clinical practice and scientific research [5], especially since the burden of COPD is high worldwide [6-7].

Various studies have examined clinically relevant thresholds for change on the CAT, CCQ and SGRQ in order to be able to evaluate and interpret treatment effects [8-18]. The Minimal Clinically Important Difference (MCID) is a parameter that quantifies this threshold. It has been defined as *"the smallest difference in score, which patients perceive as beneficial and which would mandate a change in the patient's management"* [19]. MCIDs are particularly interesting for health status questionnaires, where a change in its score is not intuitively meaningful. Change exceeding the level of the MCID can be considered clinically relevant, thus justifying therapy and help developing guidelines. It is pivotal that clinically relevant thresholds for change on a health status tool are rigorously studied and analysed carefully.

Most clinical studies that determine the MCID of Patient-Reported Outcomes (PROs) are executed in the context of an intervention such as pharmacotherapy or Pulmonary Rehabilitation (PR). This usually results in an improvement in the patients' Health-Related Quality of Life (HRQoL). MCIDs for improvement have thus been investigated upon; however there is a lack of evidence for the MCIDs for deterioration [20]. It remains unclear and debated upon to what extent clinically relevant thresholds for improvement should be similar to those for deterioration [21-24]. Certain studies outside the field of COPD have analysed the MCIDs of PROs and found evidence that values for improvement differed from deterioration [25-29]. On the other hand, there is also evidence that thresholds might be similar [30]. Interpreting worsening of HRQoL is of major importance, since one needs to differentiate between real worsening of patients' status and random variations. Furthermore, the effects of therapy may also halt further deterioration especially for a progressive chronic disease like COPD. So no relevant worsening or a reduction in clinically relevant deterioration over time might also be considered a success of therapy and in clinical trials [31].

For peer review only



For peer review only - http://bmjopen.bmj.com/site/about/guidelines.xhtml

In COPD health status, the estimated MCID for the CAT score is 2.00 to 3.00 units [11-15, 20]; for the CCQ score 0.40 to 0.50 units [8-13, 20]; and for the SGRQ score 4.00 to 8.00 units [12, 16-18, 20]. This is valid for improvement only, as there were too few patients with deterioration to investigate. There are currently no studies that specifically investigate clinically relevant thresholds for deterioration on these PROs. It is however worrying that up to date, multiple studies include the MCIDs of these COPD health status instruments for improvement to interpret deterioration in clinical trials [32-34] This study therefore aimed to determine and compare clinically relevant thresholds for deterioration and improvement on the COPD health status questionnaires CAT, CCQ and SGRQ in both a PR and Routine Clinical Practice (RCP) setting.

For peer review only



For peer review only - http://bmjopen.bmj.com/site/about/guidelines.xhtml

## Patients and methods

### Study subjects

This study was a retrospective analysis of data obtained from two prospective clinical trials. Study one was a secondary analysis of a subsample from the Routine Inspiratory Muscle Training within COPD Rehabilitation (RIMTCORE) real-life randomized controlled trial in the Klinik Bad Reichenhall, Center for Rehabilitation, Pulmonology and Orthopedics in Germany [12, 35]. Patients were recruited upon arrival in the clinic between February 2013 and July 2014. Participants were included if they had COPD category GOLD II-IV, were aged ≥18 years and gave informed consent [12, 35]. Exclusion criteria were the presence of other respiratory co-morbidities (e.g. bronchiectasis, asthma, history of bronchial carcinoma, sarcoidosis, tuberculosis); or alpha-1-antitrypsin deficiency.

Study two (MCID study) was an observational trial of COPD patients GOLD I-IV aged ≥40 years without other respiratory co-morbidities or alpha-1-antitrypsin deficiency. Patients were recruited from Dutch primary and secondary Routine Clinical Practice (RCP) between September 2015 and September 2016. Patients were approached via multiple general practices, hospitals and the Dutch patient lung federation. The study was evaluated by the Medical Ethical Committee of the University Medical Center Groningen (UMCG), the Netherlands. All patients provided written informed consent.

### Patient and public involvement

In both studies, patients and the public have not actively been involved during the design of the study nor the assessment of the burden. Summary results are disseminated to participating patients after completion.

### Study design and data collection

Patients in study one participated in an intensive 3-week full-day inpatient PR program tailored to the patient's individual needs. Details have been presented previously [12, 35]. Patient descriptives and post-bronchodilator spirometry were collected at baseline and discharge in the clinic. Patients in study two received routine care from their physician according to national treatment guidelines. Evaluation of health status over a 12-months period was the primary measurement outcome. Patient descriptives and spirometry data were obtained at baseline. Spirometry results were obtained via the including physician after approval of the participant.

Primary outcomes selected from both prospective studies for this retrospective analysis were the CAT (no recall period), CCQ (weekly version) and SGRQ (monthly version). In study one, these questionnaires were collected at



For peer review only - http://bmjopen.bmj.com/site/about/guidelines.xhtml

baseline, at PR discharge and during follow-up at three, six, nine and 12 months. Baseline and discharge measurements were taken in the clinic, where patients were blinded to their baseline scores. Follow-up questionnaires were sent by mail. In study two, all questionnaires were sent by mail and scored at home at baseline, three, six and 12 months. For this retrospective analysis baseline and follow-up scores at three, six and 12 months were included, to allow for sufficient time for deterioration in HRQoL, to include various time periods of measurement, and to allow for comparison between both study settings.

The CAT is an eight-item one-dimensional scale with item scores ranging 0-5 (0: no impairment, 5: maximum impairment) and a total score summing up to a maximum of 40 [2]. The CCQ consists of ten items scoring 0-6 (0: no impairment, 6: maximum impairment) [3]. The items cover the domains symptoms (four items), functional status (four items) and mental status (two items). Total and domain scores on the CCQ derive from adding up relevant item scores and dividing this by the number of items. The SGRQ has 50 items classified into the domains symptoms (eight items), activities (16 items) and impact (26 items) [4]. Domain and total SGRQ scores can range from 0-100 (0: no impairment, 100: maximum impairment). A 15-point Likert scale anchor question (Global Rating of Change GRC) was scored retrospectively by the patient at each follow-up visit in both datasets. The GRC required patients to assess their COPD health status compared to baseline. The answers were marked on a scale from -7 to +7, ranging from *very much worse* to *very much better* and zero equalling *no change* [36-37].

**Study methods**

All change scores for the total scores of the CAT, CCQ and SGRQ were calculated as the difference between baseline and the respective follow-up visit (three, six and 12 months). Negative change on all questionnaires represented improvement, positive change deterioration. First, in the anchor-based approach, changes on the health status instruments were classified using the corresponding score on the GRC. Scores of 0 and ±1 on the GRC indicated *no change*; scores of ±2 and ±3 represented a *minimal improvement/deterioration*; scores of ±4 and ±5 were summarized as a *moderate improvement/deterioration*; and scores of ±6 and ±7 indicated a *large improvement/deterioration* [36-37]. MCID estimates for both improvement and deterioration on the CAT, CCQ and SGRQ were calculated as the mean change scores including 95% Confidence Interval (95%CI) of those patients indicating a minimal improvement/deterioration (±2 and ±3) on the GRC for each follow-up visit, verifying normality of distribution. Mean estimates including 95%CI were determined in a similar way for patients indicating no change (GRC 0 and ±1), moderate change (GRC ±4 and ±5) and large change (GRC ±6 and ±7). Second, the distribution-based method half Standard Deviation (0.5 SD) of the change score was calculated for improved and deteriorating health status patients at respective follow-up visits [38].



For peer review only - http://bmjopen.bmj.com/site/about/guidelines.xhtml

**Data analysis**

Data analysis was performed using SPSS 24.0 (IBM, Chicago, USA). Descriptives were evaluated at baseline for either frequencies with percentages (%), mean with Standard Deviation (SD) or median with range. This was depending on the variable characteristics and/or normality of distribution. Health status data on the CCQ, CAT and SGRQ were evaluated at baseline (T0), three months (T3), six months (T6) and after 12 months (T12). Normality of distribution was verified using skewness and kurtosis. Values between -1 and +1 were considered indicative for normality. Data were checked for floor- and ceiling effects defined as over 15% of patients scoring in the lowest and highest 10% of the maximum scale range [39]. Mean and standard deviations (or median and range) were calculated at each measurement moment for all patients, as well as specifically for patients with improved and deteriorated health status change scores. Baseline scores were compared between improving and deteriorating patients, and tested using independent t-tests after verifying normality of distribution. Baseline scores were compared between both datasets (PR vs. RCP) using independent t-tests, Man-Whitney U tests or Chi-Square tests depending on the variable characteristic and/or normality of distribution. Health status change scores were all calculated in comparison to baseline. Follow-up scores were compared with baseline to test for significance of change using paired t-tests verifying normality of distribution.

In order to determine the clinically relevant thresholds for change, first correlations between the GRC and the CCQ, CAT and SGRQ were assessed using Pearson or Spearman correlation coefficients depending on normality of distribution. Correlations needed to be ≥0.30 (preferably ≥0.50) to be eligible as anchor [22]. Correlations were not only assessed between GRC and questionnaire change scores, but also between GRC, baseline and follow-up questionnaire score to assess for a possible *response shift*. Next, participants were categorized according to their GRC score at each follow-up. Mean changes (95%CI) for each respective category were determined to define thresholds for clinically relevant change. Significance of change for each GRC class at the respective follow-up visit was compared to baseline and assessed with paired t-tests verifying normality of the data. Last, the 0.5SD of the change score was determined for patients with improved and deteriorating health status change scores separately at each follow-up. Thresholds were compared between both study settings (PR vs. RCP).

An absolute overall weighted mean MCID estimate for both improvement and deterioration was calculated at the end by multiplying the number of observations (n) at each follow-up visit times the MCID estimate for that period. The sum was divided by the total number of observations. Anchor-based and distribution-based approaches had similar weights. Estimates for improvement and deterioration were compared visually in a plot.



For peer review only - http://bmjopen.bmj.com/site/about/guidelines.xhtml

## Results

### Patient characteristics

Study one included 451 patients with completed baseline data (Table 1) [12, 35]. During follow-up 355 patients (78.7%) had completed data at T3; 319 patients (70.7%) at T6; and 309 patients (68.5%) at T12. During the 12-months follow-up eight patients passed away; 41 dropped out at own request; and a varying number of non-response was present. Study two included 207 patients with full baseline data (Table 1), of whom 201 (97.1%) completed T3, 186 (89.9%) T6 and 177 (85.6%) T12. Four patients died; 12 patients discontinued at own request; and a various number of non-response was present.

There were no significant baseline differences between completers and non-completers of the 12-months follow-up in both studies, except that significantly more females (28.4%) compared with men (10.0%) did not complete the follow-up during RCP. Significant differences in age, Forced Expiratory Volume in one second percentage predicted (FEV1%pred) and health status were observed between both studies (Table 1).

*Table 1: Baseline patient characteristics*

| | Study 1: PR | Study 2: RCP | Significance testing |
|---|---|---|---|
| N (number of patients) | 451 | 207 | - |
| Age (years)[a] | 57.87 ± 6.56 | 66.69 ± 7.91 | *P < 0.001** |
| Gender (male)[b] | 293 (65.0) | 121 (58.5) | *P = 0.507* |
| FEV1%pred[a] | 50.40 ± 15.11 | 57.06 ± 21.96 | *P = 0.001** |
| GOLD I[b] | - | 35 (17.4) | *P = 0.199* |
| GOLD II | 227 (50.3) | 80 (39.8) | |
| GOLD III | 176 (39.0) | 61 (30.3) | |
| GOLD IV | 48 (10.6) | 25 (12.4) | |
| Smoking pack years[a] | 40 (30-50) | 37.5 (22.50-51.25) | *P = 0.081* |
| CAT Total[a] | 20.23 ± 7.33 | 18.32 ± 7.22 | *P = 0.002** |
| CCQ Total[a] | 2.86 ± 1.17 | 2.12 ± 1.02 | *P < 0.001** |
| CCQ Symptoms[a] | 2.87 ± 1.24 | 2.48 ± 1.03 | *P < 0.001** |
| CCQ Functional Status[a] | 2.86 ± 1.34 | 2.28 ± 1.40 | *P < 0.001** |
| CCQ Mental Status[a] | 2.86 ± 1.74 | 1 (0-1.50) | *P < 0.001** |
| SGRQ Total[a] | 50.69 ± 17.33 | 42.88 ± 19.16 | *P < 0.001** |
| SGRQ Symptoms[a] | 63.66 ± 21.77 | 48.04 ± 24.16 | *P < 0.001** |
| SGRQ Activities[a] | 63.58 ± 19.82 | 61.48 ± 21.10 | *P = 0.259* |
| SGRQ Impact[a] | 39.21 ± 18.81 | 30.52 ± 19.73 | *P < 0.001** |
| mMRC[a] | 2 (2-4) | 1 (1-2) | *P < 0.001** |

[a] Data were expressed as mean ± standard deviation or median (IQR).
[b] Data were expressed as frequencies (% of total).

* Significance testing at level $p < 0.05$ using unpaired T-tests, Man Whitney-U tests or Chi Square tests.

*Abbreviations:* CAT, COPD Assessment Test; CCQ, Clinical COPD Questionnaire; FEV1%pred, Forced Expiratory Volume in one Second % predicted; GOLD, Global Initiative for Chronic Obstructive Lung Disease; mMRC, modified Medical Research Council Dyspnea Scale; N, Number of Patients; PR, Pulmonary Rehabilitation; RCP, Routine Clinical Practice; SGRQ, St. George's Respiratory Questionnaire

For peer review only



For peer review only - http://bmjopen.bmj.com/site/about/guidelines.xhtml

139 ***Health status scores for improvement and deterioration***

140 In study one and two, CAT, CCQ and SGRQ total were normally distributed at baseline and follow-up. Completed
141 pairs of change scores (follow-up vs. baseline) were included (pair-wise deletion). Floor- and ceiling effects were
142 negligible. Mean health status baseline scores were significantly different for PR and RCP (Table 1). Overall, 58-
143 59% of patients had *improved* health status scores (negative change) at T12 after PR; compared with 44-46%
144 during RCP (Table 2). After PR mean changes observed on the CAT questionnaire at T12 were -5.45±4.66 for
145 improvers and 5.47±4.22 for patients who deteriorated; on the CCQ questionnaire -0.87±0.72 for improvement
146 and 0.83±0.62 for deterioration; and on the SGRQ questionnaire -13.83±10.43 for improvers and 10.19±8.94 for
147 (Table 2). These estimates were in RCP for the CAT -4.53±3.15 for improvement and 3.88±2.59 for deterioration;
148 for the CCQ -0.54±0.54 for improvement and 0.51±0.39 for deterioration; and for the SGRQ -7.74±9.51 for
149 improvement on and 8.46±7.06 for deterioration (Table 2).

150 There were no baseline differences in terms of age, gender and GOLD classification between improved health
151 status patients and those who deteriorated at T12 in both studies. Patients with a worse (read higher) CAT, CCQ
152 or SGRQ baseline score prior to PR had significantly more improved health status after one year. Patients, who
153 improved during RCP, had a significantly higher baseline FEV1%pred.

154

For peer review only



For peer review only - http://bmjopen.bmj.com/site/about/guidelines.xhtml

*Table 2: Health status baseline and change scores for all, improved and deteriorated patients during PR and Routine Clinical Practice (RCP)*

| | Change after 3 months (T3) | N | Change after 6 months (T6) | N | Change after 12 months (T12) | N |
|---|---|---|---|---|---|---|
| **CAT** | | | | | | |
| *All patients PR* | -1.44* (-2.16 to -0.71) | 354 | -0.91* (-1.66 to -0.16) | 319 | -0.89* (-1.68 to -0.11) | 309 |
| *Improvement PR* | -5.45±4.57 | 227 (64.1) | -5.49±4.33 | 184 (57.7) | -5.45±4.66 | 180 (58.3) |
| *Deterioration PR* | 5.75±4.20 | 127 (35.9) | 5.33±4.10 | 135 (42.3) | 5.47±4.22 | 129 (41.7) |
| *All patients RCP* | 0.30 (-0.42 to +1.02) | 201 | 0.18 (-0.53 to +0.90) | 186 | 0.14 (-0.59 to +0.87) | 177 |
| *Improvement RCP* | -4.04±3.33 | 102 (50.7) | -4.64±3.05 | 81 (43.5) | -4.53±3.15 | 79 (44.6) |
| *Deterioration RCP* | 4.23±3.66 | 83 (41.3) | 3.76±2.88 | 91 (48.9) | 3.88±2.59 | 86 (48.6) |
| *No change RCP* | - | 16 (8.0) | - | 14 (7.5) | - | 12 (6.8) |
| **CCQ Total** | | | | | | |
| *All patients PR* | -0.26* (-0.37 to -0.15) | 355 | -0.11 (-0.23 to +0.01) | 319 | -0.16* (-0.28 to -0.04) | 309 |
| *Improvement PR* | -0.88±0.71 | 225 (63.4) | -0.84±0.68 | 181 (56.7) | -0.87±0.72 | 180 (58.3) |
| *Deterioration PR* | 0.82±0.68 | 130 (36.6) | 0.84±0.67 | 138 (43.3) | 0.83±0.62 | 129 (41.7) |
| *All patients RCP* | 0.00 (-0.09 to +0.08) | 200 | 0.00 (-0.10 to +0.10) | 185 | -0.02 (-0.12 to +0.09) | 174 |
| *Improvement RCP* | -0.45±0.37 | 96 (48.0) | -0.52±0.51 | 87 (47.0) | -0.54±0.54 | 77 (44.3) |
| *Deterioration RCP* | 0.50±0.38 | 89 (44.5) | 0.56±0.46 | 80 (43.2) | 0.51±0.39 | 88 (50.6) |
| *No change RCP* | - | 15 (7.5) | - | 18 (9.7) | - | 9 (5.2) |
| **SGRQ Total** | | | | | | |
| *All patients PR* | -5.35* (-6.92 to -3.78) | 350 | -4.85* (-6.47 to -3.23) | 312 | -3.94* (-5.67 to -2.21) | 306 |
| *Improvement PR* | -13.11±9.65 | 237 (67.7) | -13.51±9.88 | 193 (61.9) | -13.83±10.43 | 180 (58.8) |
| *Deterioration PR* | 10.93±10.18 | 113 (32.3) | 8.19±8.92 | 119 (38.1) | 10.19±8.94 | 126 (41.2) |
| *All patients RCP* | -0.52 (-1.77 to +0.73) | 198 | -1.34 (-2.76 to +0.07) | 184 | -0.87 (-2.60 to +0.86) | 174 |
| *Improvement RCP* | -6.61±5.58 | 97 (49.0) | -7.91±5.52 | 75 (40.8) | -7.74±9.51 | 81 (46.6) |
| *Deterioration RCP* | 7.36±5.49 | 101 (51.0) | 7.78±6.18 | 108 (58.7) | 8.46±7.06 | 92 (52.9) |
| *No change RCP* | - | 0 | - | 1 (0.5) | - | 1 (0.6) |

Change was calculated compared with baseline. Negative change represents improvement for CAT, CCQ and SGRQ. Change scores for all patients reported as mean (95%CI). Change scores for improvement and deterioration are presented as mean ± SD.

*Paired t-tests were significant at level $p<0.05$ testing follow-up versus baseline measurements.

*Abbreviations:* 95%CI, 95% Confidence Interval; CAT, COPD Assessment Test; CCQ, Clinical COPD Questionnaire; N, Number of patients; PR, Pulmonary Rehabilitation; RCP, Routine Clinical Practice; SD, Standard Deviation; SGRQ, St. George's Respiratory Questionnaire; T3, Three months follow-up; T6, Six months follow-up; T12, 12 months follow-up.

For peer review only



For peer review only - http://bmjopen.bmj.com/site/about/guidelines.xhtml

***Clinically Important Improvement versus Deterioration***

Significant correlations between the health status change scores and the GRC ranged respectively for study one -0.33 to -0.41 (CAT), -0.42 to -0.47 (CCQ), and -0.48 to -0.54 (SGRQ) (Table 3). These ranges were for study two respectively -0.29 to -0.37, -0.38 to -0.48, and -0.35 to -0.44. GRC scores had stronger correlations with the respective follow-up health status score compared with baseline and change scores for both studies.

*Table 3: Correlations between health status (change) scores and the Global Rating of Change (GRC)*

| | **GRC T3-T0** | | **GRC T6-T0** | | **GRC T12-T0** | |
|---|---|---|---|---|---|---|
| | *PR (N=355)* | *RCP (N=201)* | *PR (N=319)* | *RCP (N=186)* | *PR (N=309)* | *RCP (N=177)* |
| CAT Change Score | -0.33* | -0.29* | -0.40* | -0.30* | -0.41* | -0.37* |
| CAT T0 | -0.31* | -0.11 | -0.25* | -0.22* | -0.34* | -0.22* |
| CAT T3 | **-0.56*** | -0.31* | **-0.50*** | -0.31* | **-0.50*** | -0.33* |
| CAT T6 | - | - | **-0.55*** | -0.40* | **-0.59*** | -0.34* |
| CAT T12 | - | - | - | - | **-0.64*** | -0.48* |
| | | | | | | |
| CCQ Change Score | -0.42* | -0.38* | -0.44* | -0.40* | -0.47* | -0.48* |
| CCQ T0 | -0.26* | -0.14* | -0.19* | -0.22* | -0.29* | -0.23* |
| CCQ T3 | **-0.61*** | -0.35* | **-0.52*** | -0.26* | **-0.54*** | -0.33* |
| CCQ T6 | - | - | **-0.56*** | -0.43* | **-0.59*** | -0.39* |
| CCQ T12 | - | - | - | - | **-0.66*** | **-0.51*** |
| | | | | | | |
| SGRQ Change Score | -0.48* | -0.35* | **-0.51*** | -0.33* | **-0.54*** | -0.44* |
| SGRQ T0 | -0.28* | -0.13 | -0.24* | -0.20* | -0.32* | -0.22* |
| SGRQ T3 | **-0.62*** | -0.29* | **-0.56*** | -0.25* | **-0.58*** | -0.28* |
| SGRQ T6 | - | - | **-0.61*** | -0.35* | **-0.62*** | -0.35* |
| SGRQ T12 | - | - | - | - | **-0.69*** | **-0.51*** |
| | | | | | | |

Data reported as Pearson or Spearman correlation coefficients between the health status (change) scores and the GRC anchor question. Correlations $\geq$0.50 are highlighted bold.

* Correlations are significant at level $p < 0.05$.

*Abbreviations:* CAT, COPD Assessment Test; CCQ, Clinical COPD Questionnaire; GRC, Global Rating of Change; N, Number of Patients; PR, Pulmonary Rehabilitation; RCP, Routine Clinical Practice; SGRQ, St. George's Respiratory Questionnaire; T0, Baseline measurement; T3, Three months follow-up; T6, Six months follow-up; T12, 12 months follow-up.

Tables 4-6 and figures 1-3 present the clinically relevant thresholds for minimal, moderate and large changes on the CAT, CCQ and SGRQ during PR and Routine Clinical Practice (RCP). On the CAT anchor- and distribution-based estimates ranged -2.80 to -2.17 (weighted mean -2.51) for minimal improvement and 2.05 to 4.21 for minimal deterioration (weighted mean 2.76) during PR (Table 4, Figure 1). These ranges were respectively -3.78 to -1.53 (weighted mean -2.49) and 1.30 to 1.97 (weighted mean 1.65) during RCP. On the CCQ minimal clinically important improvements were determined at -0.50 to -0.34 (weighted mean -0.40) for PR and -0.44 to -0.19 (weighted mean -0.33) for RCP (Table 5, Figure 2). These thresholds for deterioration were 0.31 to 0.66 (weighted mean 0.43) during PR and 0.19 to 0.46 (weighted mean 0.30) during RCP. On the SGRQ estimates ranged -9.20 to -4.83 (weighted mean -6.74) for minimal improvement and 4.46 to 7.52 for minimal deterioration (weighted

For peer review only



For peer review only - http://bmjopen.bmj.com/site/about/guidelines.xhtml

mean 5.31) during PR (Table 6, Figure 3). These ranges were respectively -4.76 to -2.76 (weighted mean -4.06) and 2.75 to 7.53 (weighted mean 4.78) during RCP.

*Table 4: Estimates for clinically relevant thresholds for improvement and deterioration on the CAT*

| **CAT** | **T3-T0** | | **T6-T0** | | **T12-T0** | | **Weighted threshold** | |
|---|---|---|---|---|---|---|---|---|
| *Change* | *Improvement* | *Deterioration* | *Improvement* | *Deterioration* | *Improvement* | *Deterioration* | *Improvement* | *Deterioration* |
| | | | | | | | | |
| **Minimal change** | | | | | | | | |
| N Anchor-based PR | 107 | 36 | 96 | 42 | 88 | 43 | 291 | 121 |
| Anchor-based PR | -2.74 | 2.71 | -2.73 | 3.21 | -2.80 | 4.21 | -2.75 | 3.42 |
| N Anchor-based RCP | 12 | 27 | 14 | 36 | 18 | 46 | 32 | 82 |
| Anchor-based RCP | - | - | -2.86 | 1.97 | -3.78 | 1.63 | -3.38 | 1.78 |
| | | | | | | | | |
| N distribution-based PR | 227 | 127 | 184 | 135 | 180 | 129 | 591 | 391 |
| Distribution-based PR | -2.29 | 2.10 | -2.17 | 2.05 | -2.33 | 2.11 | -2.26 | 2.09 |
| N distribution-based RCP | 102 | 83 | 81 | 91 | 79 | 86 | 262 | 260 |
| Distribution-based RCP | -1.67 | 1.83 | -1.53 | 1.44 | -1.58 | 1.30 | -1.60 | 1.52 |
| | | | | | | | | |
| **Moderate change** | | | | | | | | |
| N Anchor-based PR | 51 | 9 | 45 | 7 | 37 | 10 | 133 | 17 |
| Anchor-based PR | -5.02 | - | -3.29 | 8.14 | -4.27 | 6.30 | -4.23 | 7.06 |
| N Anchor-based RCP | 5 | 8 | 12 | 9 | 5 | 9 | - | 9 |
| Anchor-based RCP | - | - | - | - | - | 3.89 | - | 3.89 |
| | | | | | | | | |
| **Large change** | | | | | | | | |
| N Anchor-based PR | 16 | 3 | 12 | 2 | 14 | 3 | 42 | - |
| Anchor-based PR | -4.19 | - | -7.00 | - | -6.07 | - | -5.62 | - |
| N Anchor-based RCP | 4 | 3 | 0 | 2 | 9 | 4 | 13 | 4 |
| Anchor-based RCP | -6.00 | - | - | - | -4.22 | 5.75 | -4.77 | 5.75 |
| | | | | | | | | |
| **No change** | | | | | | | | |
| N Anchor-based PR | 133 | | 115 | | 114 | | 362 | |
| Anchor-based PR | 0.03 | | -0.01 | | -0.33 | | -0.10 | |
| N Anchor-based RCP | 141 | | 113 | | 83 | | 337 | |
| Anchor-based RCP | -0.16 | | -0.54 | | -0.47 | | -0.36 | |

Data reported as clinically relevant threshold or N. Negative change represents improvement for all health status instruments. Paired t-tests were applied with significance level at p <0.05. Non- significant results were excluded, except for the "*No change*" group.

*Abbreviations:* CAT, COPD Assessment Test; GRC, Global Rating of Change; N, Number of Patients; PR, Pulmonary Rehabilitation; RCP, Routine Clinical Practice; T0, Baseline measurement; T3, Three months follow-up; T6, Six months follow-up; T12, 12 months follow-up.

For peer review only



For peer review only - http://bmjopen.bmj.com/site/about/guidelines.xhtml

*Table 5: Estimates for clinically relevant thresholds for improvement and deterioration on the CCQ*

| **CCQ** | **T3-T0** | | **T6-T0** | | **T12-T0** | | **Weighted threshold** | |
|---|---|---|---|---|---|---|---|---|
| *Change* | *Improvement* | *Deterioration* | *Improvement* | *Deterioration* | *Improvement* | *Deterioration* | *Improvement* | *Deterioration* |
| | | | | | | | | |
| **Minimal change** | | | | | | | | |
| N Anchor-based PR | 107 | 36 | 96 | 42 | 88 | 43 | 291 | 121 |
| Anchor-based PR | -0.44 | 0.42 | -0.42 | 0.48 | -0.50 | 0.66 | -0.45 | 0.53 |
| N Anchor-based RCP | 12 | 27 | 14 | 36 | 18 | 46 | 32 | 82 |
| Anchor-based RCP | - | - | -0.44 | 0.46 | -0.38 | 0.33 | -0.41 | 0.39 |
| | | | | | | | | |
| N distribution-based PR | 225 | 130 | 181 | 138 | 180 | 129 | 586 | 397 |
| Distribution-based PR | -0.36 | 0.34 | -0.34 | 0.34 | -0.36 | 0.31 | -0.35 | 0.33 |
| N distribution-based RCP | 96 | 89 | 87 | 80 | 77 | 88 | 260 | 257 |
| Distribution-based RCP | -0.19 | 0.19 | -0.26 | 0.23 | -0.27 | 0.20 | -0.24 | 0.21 |
| | | | | | | | | |
| **Moderate change** | | | | | | | | |
| N Anchor-based PR | 51 | 9 | 45 | 7 | 37 | 10 | 133 | 7 |
| Anchor-based PR | -0.86 | - | -0.72 | 1.23 | -0.90 | - | -0.82 | 1.23 |
| N Anchor-based RCP | 5 | 8 | 12 | 9 | 5 | 9 | - | 17 |
| Anchor-based RCP | - | 0.85 | - | - | - | 0.42 | - | 0.62 |
| | | | | | | | | |
| **Large change** | | | | | | | | |
| N Anchor-based PR | 16 | 3 | 12 | 2 | 14 | 3 | 42 | - |
| Anchor-based PR | -0.96 | - | -1.03 | - | -1.18 | - | -1.05 | - |
| N Anchor-based RCP | 4 | 3 | 0 | 2 | 9 | 4 | 9 | 4 |
| Anchor-based RCP | - | - | - | - | -1.12 | 0.98 | -1.12 | 0.98 |
| | | | | | | | | |
| **No change** | | | | | | | | |
| N Anchor-based PR | 133 | | 115 | | 114 | | 362 | |
| Anchor-based PR | -0.07 | | 0.17 | | 0.10 | | 0.06 | |
| N Anchor-based RCP | 141 | | 113 | | 83 | | 337 | |
| Anchor-based RCP | -0.03 | | -0.10 | | -0.04 | | -0.06 | |

Data reported as clinically relevant threshold or N. Negative change represents improvement for all health status instruments. Paired t-tests were applied with significance level at $p < 0.05$. Non- significant results were excluded, except for the "*No change*" group.

*Abbreviations:* CCQ, Clinical COPD Questionnaire; GRC, Global Rating of Change; N, Number of Patients; PR, Pulmonary Rehabilitation; RCP, Routine Clinical Practice; T0, Baseline measurement; T3, Three months follow-up; T6, Six months follow-up; T12, 12 months follow-up.

For peer review only

*Table 6: Estimates for clinically relevant thresholds for improvement and deterioration on the SGRQ*

| **SGRQ** | **T3-T0** | | **T6-T0** | | **T12-T0** | | **Weighted threshold** | |
|---|---|---|---|---|---|---|---|---|
| *Change* | *Improvement* | *Deterioration* | *Improvement* | *Deterioration* | *Improvement* | *Deterioration* | *Improvement* | *Deterioration* |
| | | | | | | | | |
| **Minimal change** | | | | | | | | |
| N Anchor-based PR | 107 | 36 | 96 | 42 | 88 | 43 | 291 | 121 |
| Anchor-based PR | -7.58 | 5.01 | -9.20 | 5.14 | -8.82 | 7.52 | -8.49 | 5.95 |
| N Anchor-based RCP | 12 | 27 | 14 | 36 | 18 | 46 | 14 | 82 |
| Anchor-based RCP | - | - | -4.70 | 7.53 | - | 5.60 | -4.70 | 6.45 |
| | | | | | | | | |
| N distribution-based PR | 237 | 113 | 193 | 119 | 180 | 126 | 610 | 358 |
| Distribution-based PR | -4.83 | 5.09 | -4.94 | 4.46 | -5.22 | 4.47 | -4.98 | 4.66 |
| N distribution-based RCP | 97 | 101 | 75 | 108 | 81 | 92 | 253 | 301 |
| Distribution-based RCP | -2.79 | 2.75 | -2.76 | 3.09 | -4.76 | 3.53 | -3.41 | 3.11 |
| | | | | | | | | |
| **Moderate change** | | | | | | | | |
| N Anchor-based PR | 51 | 9 | 45 | 7 | 37 | 10 | 124 | 10 |
| Anchor-based PR | -15.85 | - | -13.63 | - | -15.40 | 9.30 | -16.06 | 9.30 |
| N Anchor-based RCP | 5 | 8 | 12 | 9 | 5 | 9 | - | 9 |
| Anchor-based RCP | - | - | - | - | - | 7.46 | - | 7.46 |
| | | | | | | | | |
| **Large change** | | | | | | | | |
| N Anchor-based PR | 16 | 3 | 12 | 2 | 14 | 3 | 42 | - |
| Anchor-based PR | -18.33 | - | -21.99 | | -20.58 | - | -20.13 | - |
| N Anchor-based RCP | 4 | 3 | 0 | 2 | 9 | 4 | 9 | - |
| Anchor-based RCP | - | - | - | - | -18.70 | - | -18.70 | - |
| | | | | | | | | |
| **No change** | | | | | | | | |
| N Anchor-based PR | 133 | | 115 | | 114 | | 362 | |
| Anchor-based PR | -1.50 | | -0.99 | | -0.06 | | -0.88 | |
| N Anchor-based RCP | 141 | | 113 | | 83 | | 337 | |
| Anchor-based RCP | 0.51 | | 0.19 | | 0.10 | | 0.30 | |

Data reported as clinically relevant threshold or N. Negative change represents improvement for all health status instruments. Paired t-tests were applied with significance level at $p < 0.05$. Non- significant results were excluded, except for the "*No change*" group.

*Abbreviations:* GRC, Global Rating of Change; N, Number of Patients; PR, Pulmonary Rehabilitation; RCP, Routine Clinical Practice; SGRQ, St. George's Respiratory Questionnaire; T0, Baseline measurement; T3, Three months follow-up; T6, Six months follow-up; T12, 12 months follow-up.

*Figure 1: Forrest plot of clinically relevant thresholds for improvement and deterioration on the CAT.*

*Figure 2: Forrest plot of clinically relevant thresholds for improvement and deterioration on the CCQ.*

*Figure 3: Forrest plot of clinically relevant thresholds for improvement and deterioration on the SGRQ.*

*Legend Figures 1-3: Data are presented as mean estimates (squares) including 95% confidence interval (horizontal lines). Estimates from the half standard deviation analysis are represented as single squares. Weighted mean estimates are presented as larger diamonds. Data are separated as minor, moderate and large improvement thresholds (left half), versus minor and moderate deterioration thresholds (right half).*

For peer review only

## Discussion

*Summary of main findings*

Using both anchor- and distribution-based methods, the *weighted MCIDs* for improvement and deterioration on the CAT were respectively -2.51 vs. 2.76 during PR; and -2.49 vs. 1.65 during Routine Clinical Practice (RCP). These thresholds for improvement and deterioration on the CCQ were respectively -0.40 vs. 0.43 during PR; and -0.33 vs. 0.30 during RCP. MCIDs for the SGRQ were respectively -6.74 vs. 5.31 during PR; and -4.06 vs. 4.78 during RCP for improvement and deterioration. Estimates for minimal clinically important improvement and deterioration were overall somewhat similar, however absolute MCIDs differed between PR and RCP. Thresholds for *moderate* and *large* improvement and deterioration differed from each other, as well as between study settings.

*Interpretation of findings*

Little evidence exists whether MCIDs for improvement are similar for deterioration [21, 23, 40]. Jaeschke et al. were the first to determine the MCID of a health status tool using a 15-point GRC combining both improved and deteriorated COPD patients into one group of minimally changed participants [19]. Juniper et al. elaborated on this by separating minimally improved patients from deterioration in asthma, but only a limited number of patients indicated deterioration and no conclusions upon the MCID of deterioration were drawn [37]. Outside the field of COPD, Crosby et al. and de Vet et al. stated that some studies demonstrated that a smaller MCID for improvement was required compared with deterioration [21, 40]. The current study does not confirm this; although MCIDs seemed smaller for RCP patients compared with PR. Patients experienced more change (hence larger absolute MCIDs) during intervention, possibly as a result of treatment. In RCP, smaller changes may be noted and regarded as relevant for the patient. Overall, the absolute values for the MCIDs for improvement and deterioration did not seem to differ much here, with the exception of the SGRQ during PR.

The ranges found in this study for the MCID of the CAT *(improvement -3.78 to -1.53; deterioration 1.30 to 4.21)* matched with estimates found in other studies [11-15, 20]. Two studies used a patient-assessed GRC to estimate the MCID of the CAT [14-15]. However, no results were reported for worsened patients or the numbers of patients were too few. Other anchor-based methods suggested that a change of one point on the CAT might represent the MCID for deterioration [14]. The weighted thresholds for minimal clinically relevant improvement *(-2.51 in PR and -2.49 in RCP)* seemed somewhat comparable with the ones for deterioration *(2.76 in PR and 1.65 in RCP)* in the current study, except for deterioration during routine clinical practice. As CAT allows only integer scores [2], a change of three points seems a valid threshold for improvement and deterioration, although the MCID for



For peer review only - http://bmjopen.bmj.com/site/about/guidelines.xhtml

deterioration in RCP could be closer to two points. Thresholds for moderate improvement *(-4.23 in PR)* and deterioration *(7.06 in PR and 3.89 in RCP)* turned out less similar. The number of patients moderately deteriorating was low and differences were observed between both study settings. Moderate change might be experienced with a change on the CAT score of 4-7 points. Two previous studies suggested that a cut-off point of four points was identified for acute HRQoL deterioration in clinical practice [41-42]. This would match our estimates for moderate change. The number of patients with a large change was too low with wide confidence intervals to enable valid conclusions.

Regarding the CCQ, the MCID ranges found for both improvement *(-0.50 to -0.19)* and deterioration *(0.19 to 0.66)* overlapped each other in absolute sense, indicating that estimates for improvement and deterioration may be similar. However, differences were noted between PR *(±0.40)* and RCP *(±0.30)* for both minimal improvement and deterioration. These estimates for the MCID matched with earlier evidence [8-13]. One other study used a GRC to determine the MCID of the CCQ [8]. Unfortunately, no data were available on worsening patients. Thresholds for moderate change on the CCQ were broad (±0.62 to ±1.23). Few patients experienced large changes, but estimates for both types of MCID from both study settings were approximately one point.

Minimal thresholds for improvement *(-9.20 to -2.76)* and deterioration *(2.75 to 7.53)* on the SGRQ overlapped each other, although more variation was present here. A change of approximately four to seven points for both improvement and deterioration seemed to be the minimal clinically important threshold in the current study. The MCID for improvement during PR *(-6.74)* was larger than for deterioration *(5.31)*; however, confidence intervals for deterioration were wide. Estimates for the thresholds during RCP (four to five points) were smaller compared with PR (five to seven points). Moreover, the distribution-based estimates turned out smaller than the anchor-based estimates, lowering the absolute weighted MCIDs. Thresholds for moderate improvement and deterioration in the current study were not very similar ranging absolutely from 7.46 to 16.06 points. Estimates for clinically relevant large HRQoL improvement on the SGRQ ranged -20 to -18 points for PR and RC, but too few patients were included to draw valid conclusions.

The SGRQ MCID matched to some extent with previous results [12, 16-18, 20]. Jones et al. published a threshold of four points, which is generally accepted and applied in clinical practice [16, 18]. Interestingly, most results in our current study suggest a larger MCID, although estimates from RCP included this four point's estimate. The estimate by Jones et al. was based upon a study using patient preference-based techniques in COPD by applying a five-point patients' judgement of treatment efficacy (Salmeterol). This MCID of four points was valid for the

For peer review only



For peer review only - http://bmjopen.bmj.com/site/about/guidelines.xhtml

group of patients that experienced effective treatment. In addition, a clinicians' five-point GRC was scored, resulting in a MCID of four points. Clinicians' and patients' ratings are however not necessarily similar [43].

*Strengths and limitations of current study*

This retrospective analysis of two prospective studies was the first to investigate clinically relevant thresholds for minimal, moderate and large changes in COPD health status comparing both improvement and deterioration using a triangulation of both anchor- and distribution-based methods. There were sufficient correlations between the GRC and respective health status questionnaires as required [22]; although they were still only weak to moderate. It should be noted that correlations were stronger with the follow-up score compared with the baseline and/or change score, possibly due to a response shift. Another strength is that multiple follow-up visits were included to limit possible influence of the period of measurements on the MCID and recall bias [21, 24]. Moreover, this study investigated clinically relevant thresholds for both PR and a routine clinical practice, improving its clinical application and external validity.

Although this is the first study to investigate thresholds for clinically relevant deterioration, still a limited number of patients indicated deterioration in HRQoL after PR and during routine clinical practice. This is a major limitation lowering the statistical power of the analysis, especially since sample size calculations were not based upon the separate GRC categories. A second limitation is that the found thresholds demonstrate broad ranges with wide confidence intervals, limiting its accuracy and requiring a larger sample size than our current studies have. Third, it should be taken into account that anchor- and distribution-based approaches each have their own relevance, either based upon clinical retrospective assessments or statistical parameters. It is recommended to combine both methods in measuring an instrument's MCID [22], however estimates are somewhat different between these methods.

*Implications for future research and clinical practice*

COPD patients tend to have worsening HRQoL over time; hence MCIDs for deterioration have an important implication for clinical practice [44-45]. Clinicians and researchers should be able to judge whether groups of patients were really worsening over time or that change observed was subject to random fluctuation. Preventing clinically relevant deterioration in HRQoL by means of therapy is thus an important goal too. Ideally, more research is needed to validate our thresholds for clinically relevant deterioration on the CAT, CCQ and SGRQ for instance in studies other kinds of interventions than PR. One cannot directly transform the thresholds for



For peer review only - http://bmjopen.bmj.com/site/about/guidelines.xhtml

improvement into those for deterioration. Evidence outside the field of COPD has found differences. However, in the current study, the estimates turned out rather similar with differing MCIDs between studies. Setting could thus potentially impact the MCID, implying that the results in the current study not necessarily need to be valid in other settings too.

*Conclusions*

Determining deterioration in HRQoL is of importance, since one needs to differentiate between real worsening of patients' status and random variations. In this study, estimates for clinically relevant thresholds for improvement and deterioration were somewhat similar, but differed between Pulmonary Rehabilitation and Routine Clinical Practice (RCP). We would recommend using cut-points of CAT≥3 (intervention), CAT≥2 (RCP), CCQ ≥0.40 (intervention), CCQ≥0.30 (RCP), SGRQ≥6 (intervention) and SGRQ ≥5 (RCP) for both *minimal* improvement and deterioration. Thresholds for respectively *moderate* and *large* changes should be further explored, but could approximately be in the range of respectively 4-5 and 5-6 for CAT; 0.80 and 1.00 for CCQ; 10-15 points and 15-20 points for SGRQ.

For peer review only



For peer review only - http://bmjopen.bmj.com/site/about/guidelines.xhtml

## List of Abbreviations

| | |
|---|---|
| 0.5SD | Half Standard Deviation |
| 95%CI | 95% Confidence Interval |
| CAT | COPD Assessment Test |
| CCQ | Clinical COPD Questionnaire |
| COPD | Chronic Obstructive Pulmonary Disease |
| FEV1%Pred | Forced Expiratory Volume in one second % predicted |
| GOLD | Global initiative for Obstructive Lung Diseases |
| GRC | Global Rating of Change scale |
| HRQoL | Health-Related Quality of Life |
| MCID | Minimal Clinically Important Difference |
| N | Number of Patients |
| PR | Pulmonary Rehabilitation |
| PROs | Patient-Reported Outcomes |
| RCP | Routine Clinical Practice |
| RIMTCORE | Routine Inspiratory Muscle Training within COPD Rehabilitation |
| SD | Standard Deviation |
| SGRQ | St. George Respiratory Questionnaire |
| T0 | Baseline measurement |
| T3 | Time point 3 months follow-up |
| T6 | Time point 6 months follow-up |
| T12 | Time point 12 months follow-up |
| UMCG | University Medical Center Groningen |

## Appendices

Figures 1-3



For peer review only - http://bmjopen.bmj.com/site/about/guidelines.xhtml

## References

[1] Global Initiative for Chronic Obstructive Lung Disease (GOLD). Global Strategy for the Diagnosis, Management and Prevention of COPD (2017 Report). 2017; Available at http://www.goldcopd.org/gold-2017-global-strategy-diagnosis-management-prevention-copd/. Accessed 17th of July 2017.

[2] Jones, PW, Harding, G, Berry, P et al. Development and first validation of the COPD Assessment Test. Eur Respir J 2009; 34: 648-654.

[3] Van der Molen, T, Willemse, BWM, Schokker, S et al. Development, validity and responsiveness of the Clinical COPD Questionnaire. Health and Quality of Life Outcomes 2003, 1: 13.

[4] Jones, PW, Quirck, FH and Baveystock CM. The St George's Respiratory Questionnaire. Respiratory Medicine 1991; 85 (Supplement B): 25-31.

[5] Cave, J, Atkinson, L, Tsiligianni, IG et al. Assessment of COPD wellness tools for use in primary care: an IPCRG initiative. Int J Chron Obstruct Pulmon Dis. 2012; 7: 447-456.

[6] Lozano, R, Naghavi, M, Foreman, K et al. Global and regional mortality from 235 causes of death for 20 age groups in 1990 and 2010: a systematic analysis for the Global Burden of Disease Study 2010. Lancet 2012; 380: 2095-128.

[7] Adeloye, D, Chua, S, Lee, C et al. Global and regional estimates of COPD prevalence: Systematic review and meta-analysis. J Glob Health 2015; 5(2): 020415.

[8] Kocks, JW, Tuinenga, MG, Uil, SM et al. Health status measurement in COPD: the minimal clinically important difference of the clinical COPD questionnaire. Respir Res. 2006; 7: 62.

[9] Kon, SS, Dilaver, D, Mittal, M et al. The Clinical COPD Questionnaire: response to pulmonary rehabilitation and minimal clinically important difference. Thorax. 2014; 69(9): 793-798.

[10] Canavan, JL, Dilaver, D, Clark, AL et al. Clinical COPD Questionnaire in patients with chronic respiratory disease. Respirology 2014; 19(7): 1006-1012.

[11] Tsiligianni, IG, van der Molen, T, Moraitaki, D et al. Assessing health status in COPD. A head-to-head comparison between the COPD assessment test (CAT) and the clinical COPD questionnaire (CCQ). BMC Pulmonary Medicine 2012, 12: 20.



For peer review only - http://bmjopen.bmj.com/site/about/guidelines.xhtml

[12] Alma, H, de Jong, C, Jelusic, D et al. Health status instruments for patients with COPD in pulmonary rehabilitation: defining a minimal clinically important difference. Npj PCRM 2016; 26: 16041.

[13] Smid, DE, Franssen, FM, Houben-Wilke, S et al. Responsiveness and MCID Estimates for CAT, CCQ and HADS in Patients With COPD Undergoing Pulmonary Rehabilitation: A Prospective Analysis. J Am Med Dir Assoc. 2017; 18(1): 53-58.

[14] Kon, SS, Canavan, JL, Nolan, CM et al. Minimum clinically important difference for the COPD Assessment Test: a prospective analysis. Lancet Respir. Med. 2014; 2: 195-203.

[15] Dodd, JW, Hogg, L, Nolan, J et al. The COPD assessment test (CAT): response to pulmonary rehabilitation. A multicentre, prospective study. Thorax 2011; 66: 425-429.

[16] Jones, PW. St. George's Respiratory Questionnaire: MCID. COPD 2005; 2: 75-79.

[17] Welling, JB, Hartman, JE, Ten Hacken, NH et al. The minimal important difference for the St George's Respiratory Questionnaire in patients with severe COPD. Eur. Respir. J. 2016; 46: 1598.

[18] Jones, PW and Bosh, TK. Quality of life changes in COPD patients treated with salmeterol. Am. J. Respir. Crit. Care Med. 1997; 155: 1283-1289.

[19] Jaeschke, R, Singer, J and Guyatt, GH. Measurement of health status. Ascertaining the minimal clinically important difference. Control Clin. Trials 1989; 10: 407-415.

[20] Alma, H, de Jong, C, Tsiligianni, I et al. Clinically relevant differences in COPD health status: systematic review and triangulation. Eur Respir J. 2018; 6: 52(3).

[21] Crosby, RD, Kolotkin, RL and Williams, GR. Defining clinically meaningful change in health-related quality of life. Journal of Clinical Epidemiology 2003; 56: 395-400.

[22] Revicki, D, Hays, RD, Cella, D and Sloan, J. Recommended methods for determining responsiveness and minimally important differences for patient-reported outcomes. J. Clin. Epidemiol. 2008; 61: 102-109.

[23] Beaton, DE, Boers, M and Wells, GA. Many faces of the minimal clinically important difference (MCID): a literature review and directions for future research. Curr. Opin. Rheumatol. 2002; 14: 109-114.

For peer review only



For peer review only - http://bmjopen.bmj.com/site/about/guidelines.xhtml

[24] Hays, RD and Woolley, M. The Concept of Clinically Meaningful Difference in Health-Related Quality of-Life Research How Meaningful is it? Pharmacoeconomics 2000; 18(5): 419-423.

[25] Raman, S, Ding, K, Chow, E et al. Minimal clinically important differences in the EORTC QLQ-BM22 and EORTC QLQ-C15-PAL modules in patients with bone metastases undergoing palliative radiotherapy. Qual Life Res. 2016; 25(10): 2535-2541.

[26] Maringwa, J, Quinten, C, King, M et al. Minimal clinically meaningful differences for the EORTC QLQ-C30 and EORTC QLQ-BN20 scales in brain cancer patients. Ann Oncol. 2011; 22(9): 2107-2112.

[27] Hui, D, Shamieh, O, Paiva, CE et al. Minimal Clinically Important Difference in the Physical, Emotional, and Total Symptom Distress Scores of the Edmonton Symptom Assessment System. J Pain Symptom Manage 2016; 51(2): 262-269.

[28] Tuomi, L, Johansson, M, Andréll, P and Finizia, C. Interpretation of the Swedish Self Evaluation of Communication Experiences after Laryngeal cancer: Cutoff levels and minimum clinically important differences. Head Neck 2016; 38(5): 689-695.

[29] Rouquette, A, Blanchin, M, Sébille, V et al. The minimal clinically important difference determined using item response theory models: an attempt to solve the issue of the association with baseline score. J Clin Epidemiol 2014; 67(4): 433-440.

[30] Hui, D, Shamieh, O, Paiva, CE et al. Minimal clinically important differences in the Edmonton Symptom Assessment Scale in cancer patients: A prospective, multicentre study. Cancer 2015; 121(17): 3027-3035.

[31] Singh, D, D'Urzo, AD, Chuecos, F et al. Reduction in clinically important deterioration in chronic obstructive pulmonary disease with aclidinium/formoterol. Respiratory Research 2017; 18:106.

[32] Stockley, RA, Edgar, RG, Starkey, S and Turner AM. Health status decline in α-1 antitrypsin deficiency: a feasible outcome for disease modifying therapies? Respir Res. 2018; 19(1): 137.

[33] Anzueto, AR, Kostikas, K, Mezzi, K et al. Indacaterol/glycopyrronium versus salmeterol/fluticasone in the prevention of clinically important deterioration in COPD: results from the FLAME study. Respir Res. 2018; 19(1): 121.

[34] Naya, IP, Tombs, L, Muellerova, H et al. Long-term outcomes following first short-term clinically important deterioration in COPD. Respir Res. 2018; 19(1): 222.



For peer review only - http://bmjopen.bmj.com/site/about/guidelines.xhtml

For peer review only

[35] Schultz, K, Jelusic, D, Wittmann, M et al. Inspiratory Muscle Training does not improve clinical outcomes in 3-week COPD rehabilitation: Results from a Randomised Controlled Trial. Eur Respir J. 2018; 51(1): 1702000.

[36] Kamper, SJ, Maher, CG and Mackay, G. Global rating of change scales: a review of strengths and weaknesses and considerations for design. J. Man. Manip. Ther. 2009; 17: 163-170.

[37] Juniper, EF, Guyatt, GH, Willan, A et al. Determining a Minimal Important Change in a Disease-Specific Quality of Life Questionnaire. J Clin Epidemiol 1994; 47 (I): 81-87.

[38] Norman, GR, Sloan, JA and Wyrwich, KW. Interpretation of changes in health-related quality of life: the remarkable universality of half a standard deviation. Med. Care 2003; 41: 582-592.

[39] Terwee, CB, Bot SD, de Boer, MR et al. Quality criteria were proposed for measurement properties of health status questionnaires. J Clin Epidemiol 2007; 60: 34-42.

[40] de Vet, HC, Terwee, CB, Ostelo, RW et al. Minimal changes in health status questionnaires: distinction between minimally detectable change and minimally important change. Health and Quality of Life Outcome 2006; 4:54.

[41] Pothirat, C, Chaiwong, W, Limsukon, A et al. Detection of acute deterioration in health status visit among COPD patients by monitoring COPD assessment test score. International Journal of COPD 2015; 10: 277-282.

[42] García-Sidro, P, Naval, E, Martinez Rivera, C et al. The CAT (COPD Assessment Test) questionnaire as a predictor of the evolution of severe COPD exacerbations. Respir Med. 2015; 109(12): 1546-1552.

[43] Janse, AJ, Gemke, RJ, Uiterwaal, CS et al. Quality of life: patients and doctors don't always agree: a meta-analysis. J Clin Epidemiol. 2004; 57(7): 653-661.

[44] Yoo, JY, Kim, YS, Kim, SS et al. Factors affecting the trajectory of health-related quality of life in COPD patients. Int J Tuberc Lung Dis 2016; 20(6): 738-746.

[45] Sundh, J, Montgomery, S, Hasselgren, M et al. Change in health status in COPD: a seven-year follow-up cohort study. npj Primary Care Respiratory Medicine 2016; 26: 16073.

For peer review only



For peer review only - http://bmjopen.bmj.com/site/about/guidelines.xhtml

For peer review only
For peer review only - http://bmjopen.bmj.com/site/about/guidelines.xhtml

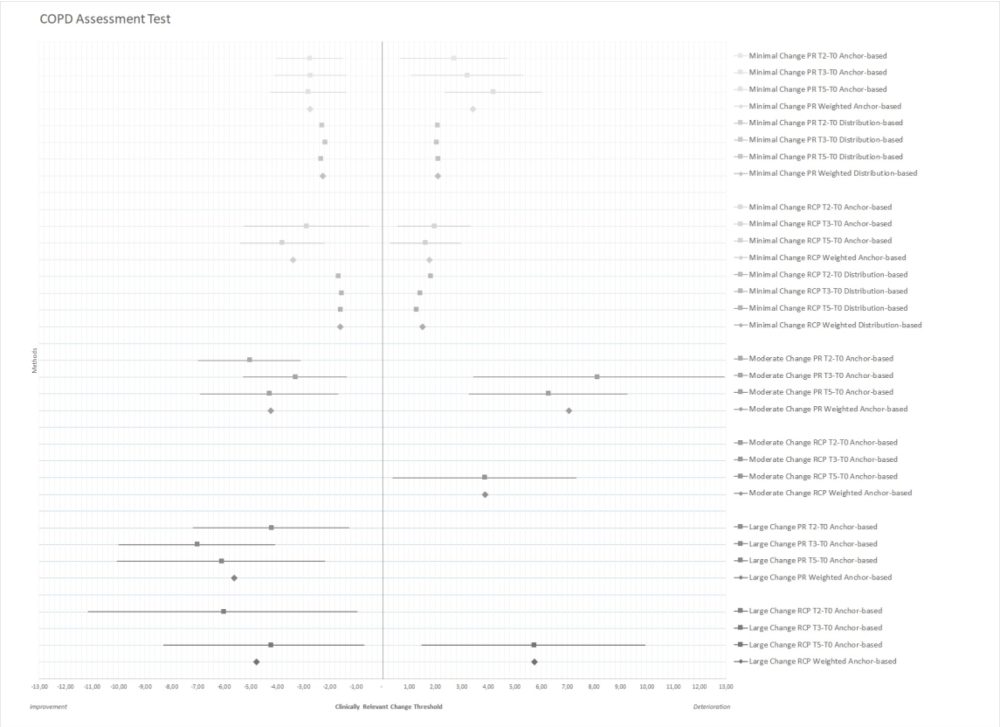


Caption: Figure 1: Forrest plot of clinically relevant thresholds for improvement and deterioration on the CAT.

Legend: Data are presented as mean estimates (squares) including 95% confidence interval (horizontal lines). Estimates from the half standard deviation analysis are represented as single squares. Weighted mean estimates are presented as larger diamonds. Data are separated as minor, moderate and large improvement thresholds (left half), versus minor and moderate deterioration thresholds (right half).

159x115mm (300 x 300 DPI)

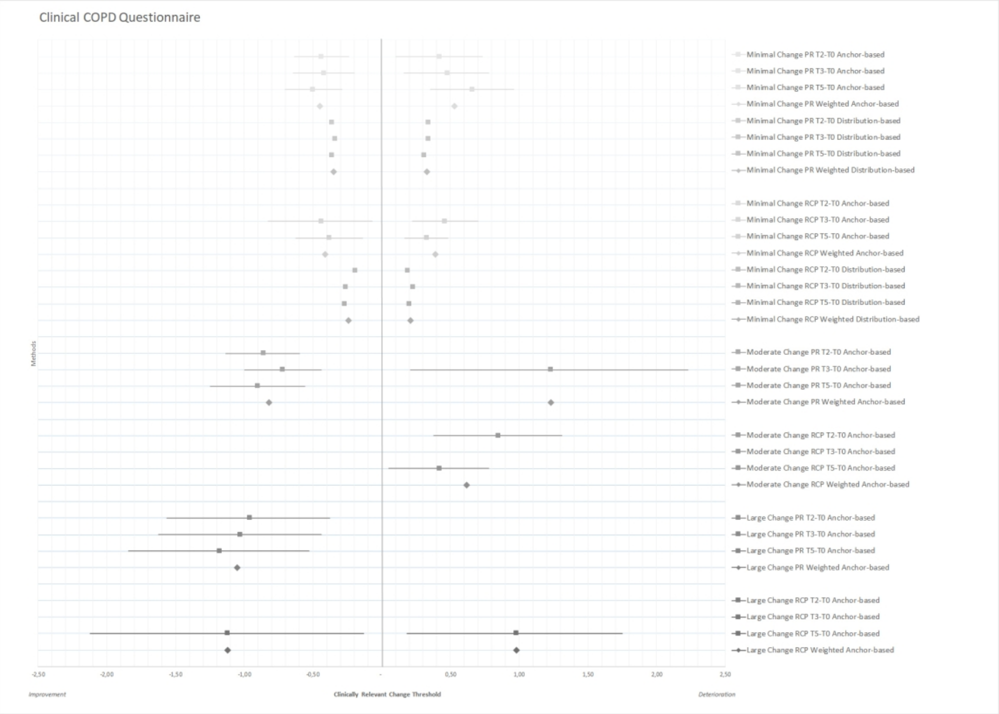


Caption: Figure 2: Forrest plot of clinically relevant thresholds for improvement and deterioration on the CCQ.

Legend: Data are presented as mean estimates (squares) including 95% confidence interval (horizontal lines). Estimates from the half standard deviation analysis are represented as single squares. Weighted mean estimates are presented as larger diamonds. Data are separated as minor, moderate and large improvement thresholds (left half), versus minor and moderate deterioration thresholds (right half).

159x114mm (300 x 300 DPI)

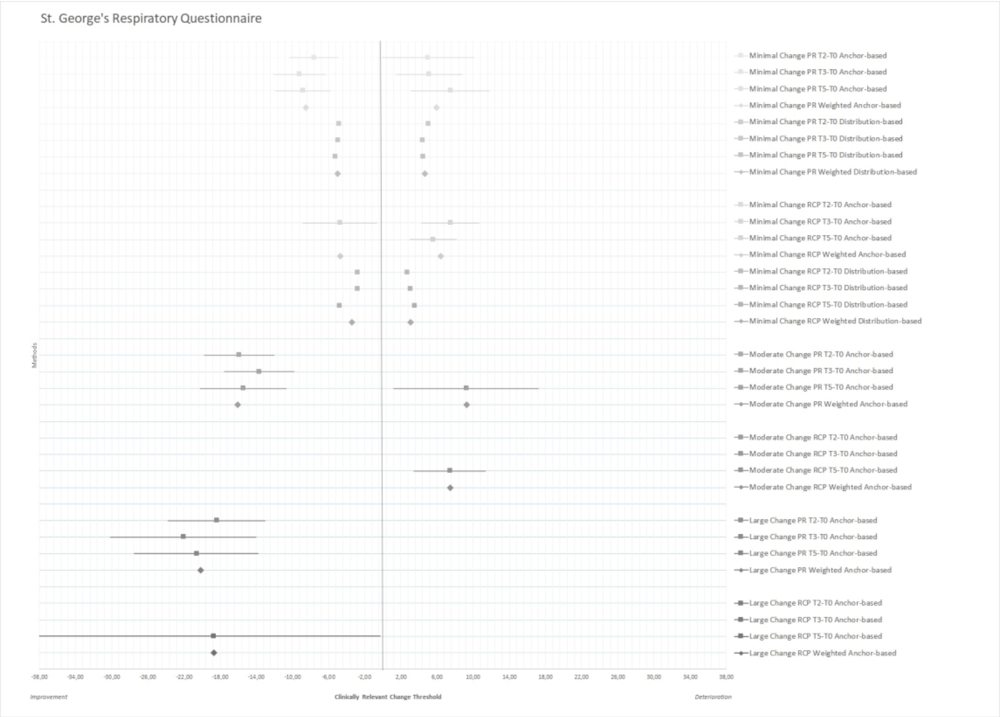


Caption: Figure 3: Forrest plot of clinically relevant thresholds for improvement and deterioration on the SGRQ.

Legend: Data are presented as mean estimates (squares) including 95% confidence interval (horizontal lines). Estimates from the half standard deviation analysis are represented as single squares. Weighted mean estimates are presented as larger diamonds. Data are separated as minor, moderate and large improvement thresholds (left half), versus minor and moderate deterioration thresholds (right half).

160x114mm (300 x 300 DPI)

STROBE Statement—checklist of items that should be included in reports of observational studies

| | Item No. | Recommendation | Page No. | Relevant text from manuscript |
|---|---|---|---|---|
| **Title and abstract** | 1 | (*a*) Indicate the study's design with a commonly used term in the title or the abstract | 0 | Title page |
| | | (*b*) Provide in the abstract an informative and balanced summary of what was done and what was found | 1 | Abstract page |
| **Introduction** | | | | |
| Background/rationale | 2 | Explain the scientific background and rationale for the investigation being reported | 7-8 | Introduction pages lines 1-36 |
| Objectives | 3 | State specific objectives, including any prespecified hypotheses | 8 | Introduction pages lines 29-36 |
| **Methods** | | | | |
| Study design | 4 | Present key elements of study design early in the paper | 9 | Methods lines 39-52 |
| Setting | 5 | Describe the setting, locations, and relevant dates, including periods of recruitment, exposure, follow-up, and data collection | 9 | Methods lines 39-52 |
| Participants | 6 | (*a*) *Cohort study*—Give the eligibility criteria, and the sources and methods of selection of participants. Describe methods of follow-up<br>*Case-control study*—Give the eligibility criteria, and the sources and methods of case ascertainment and control selection. Give the rationale for the choice of cases and controls<br>*Cross-sectional study*—Give the eligibility criteria, and the sources and methods of selection of participants | 9 | Methods lines 39-52 |
| | | (*b*) *Cohort study*—For matched studies, give matching criteria and number of exposed and unexposed<br>*Case-control study*—For matched studies, give matching criteria and the number of controls per case | N/A | N/A |
| Variables | 7 | Clearly define all outcomes, exposures, predictors, potential confounders, and effect modifiers. Give diagnostic criteria, if applicable | 9-10 | Methods lines 56-80 |
| Data sources/ measurement | 8* | For each variable of interest, give sources of data and details of methods of assessment (measurement). Describe comparability of assessment methods if there is more than one group | 9-10 | Methods lines 56-80 |
| Bias | 9 | Describe any efforts to address potential sources of bias | 4 | Text on conflict of interest |
| Study size | 10 | Explain how the study size was arrived at | 1 | Sample size calculations are presented in the original study |



For peer review only - http://bmjopen.bmj.com/site/about/guidelines.xhtml

| | | | | |
|---|---|---|---|---|
| | | | | protocols (see trial reference numbers in the abstract) |
| Quantitative variables | 11 | Explain how quantitative variables were handled in the analyses. If applicable, describe which groupings were chosen and why | 10 | Methods lines 81-94 |
| Statistical methods | 12 | (*a*) Describe all statistical methods, including those used to control for confounding | 11 | Methods lines 95-123 |
| | | (*b*) Describe any methods used to examine subgroups and interactions | 11 | Methods lines 95-123 |
| | | (*c*) Explain how missing data were addressed | 13 | Results lines 140-141 |
| | | (*d*) *Cohort study*—If applicable, explain how loss to follow-up was addressed<br>*Case-control study*—If applicable, explain how matching of cases and controls was addressed<br>*Cross-sectional study*—If applicable, describe analytical methods taking account of sampling strategy | N/A | N/A |
| | | (*e*) Describe any sensitivity analyses | 11 | Methods lines 95-123 |
| **Results** | | | | |
| Participants | 13* | (a) Report numbers of individuals at each stage of study—eg numbers potentially eligible, examined for eligibility, confirmed eligible, included in the study, completing follow-up, and analysed | 12 | Results lines 126-131 |
| | | (b) Give reasons for non-participation at each stage | 12 | Results lines 126-131 |
| | | (c) Consider use of a flow diagram | NA | N/A |
| Descriptive data | 14* | (a) Give characteristics of study participants (eg demographic, clinical, social) and information on exposures and potential confounders | 12 | Results Table 1 and lines 132-135 |
| | | (b) Indicate number of participants with missing data for each variable of interest | - | In the results section the number of participants is noted under N. This indirectly gives notice of the missing data when deducted from the number of patients at each follow-up visit. |
| | | (c) *Cohort study*—Summarise follow-up time (eg, average and total amount) | 13-14 | Results lines 139-153 and table 2 |
| Outcome data | 15* | *Cohort study*—Report numbers of outcome events or summary measures over time | 13-14 | Results lines 139-153 and table 2 |
| | | *Case-control study*—Report numbers in each exposure category, or summary measures of exposure | 13-14 | Results lines 139-153 and table 2 |
| | | *Cross-sectional study*—Report numbers of outcome events or summary measures | 13-14 | Results lines 139-153 and table 2 |



For peer review only - http://bmjopen.bmj.com/site/about/guidelines.xhtml

| | | | | |
|---|---|---|---|---|
| Main results | 16 | (*a*) Give unadjusted estimates and, if applicable, confounder-adjusted estimates and their precision (eg, 95% confidence interval). Make clear which confounders were adjusted for and why they were included | | Results lines 166-176 and tables 4-6 and figures 1-3 |
| | | (*b*) Report category boundaries when continuous variables were categorized | N/A | N/A |
| | | (*c*) If relevant, consider translating estimates of relative risk into absolute risk for a meaningful time period | N/A | N/A |
| Other analyses | 17 | Report other analyses done—eg analyses of subgroups and interactions, and sensitivity analyses | N/A | N/A |
| **Discussion** | | | | |
| Key results | 18 | Summarise key results with reference to study objectives | 19 | Discussion lines 195-201 |
| Limitations | 19 | Discuss limitations of the study, taking into account sources of potential bias or imprecision. Discuss both direction and magnitude of any potential bias | 21 | Discussion lines 253-271 |
| Interpretation | 20 | Give a cautious overall interpretation of results considering objectives, limitations, multiplicity of analyses, results from similar studies, and other relevant evidence | 19-20 | Discussion lines 202-252 |
| Generalisability | 21 | Discuss the generalisability (external validity) of the study results | 21 | Discussion lines 273-282, Conclusions lines 283-291 |
| **Other information** | | | | |
| Funding | 22 | Give the source of funding and the role of the funders for the present study and, if applicable, for the original study on which the present article is based | 4 | Text on funding |

*Give information separately for cases and controls in case-control studies and, if applicable, for exposed and unexposed groups in cohort and cross-sectional studies.

**Note:** An Explanation and Elaboration article discusses each checklist item and gives methodological background and published examples of transparent reporting. The STROBE checklist is best used in conjunction with this article (freely available on the Web sites of PLoS Medicine at http://www.plosmedicine.org/, Annals of Internal Medicine at http://www.annals.org/, and Epidemiology at http://www.epidem.com/). Information on the STROBE Initiative is available at www.strobe-statement.org.

For peer review only

BMJ Open

# Thresholds for Clinically Important Deterioration versus Improvement in COPD Health Status: Results from a Randomized Controlled Trial in Pulmonary Rehabilitation and an Observational Study during Routine Clinical Practice

| | |
|---|---|
| Journal: | *BMJ Open* |
| Manuscript ID | bmjopen-2018-025776.R2 |
| Article Type: | Research |
| Date Submitted by the Author: | 12-May-2019 |
| Complete List of Authors: | Alma, Harma; University of Groningen, University Medical Center Groningen, Department of General Practice and Elderly Care Medicine; University of Groningen, University Medical Center Groningen, Groningen Research Institute for Asthma and COPD (GRIAC)<br>de Jong, Corina; University of Groningen, University Medical Center Groningen, Department of General Practice and Elderly Care Medicine; University of Groningen, University Medical Center Groningen, Groningen Research Institute for Asthma and COPD (GRIAC)<br>Jelusic, Danijel; Klinik Bad Reichenhall, Center for Rehabilitation, Pulmonology and Orthopedics<br>Wittmann, Michael; Klinik Bad Reichenhall, Center for Rehabilitation, Pulmonology and Orthopedics<br>Schuler, Michael; Julius-Maximilians-Universitat Wurzburg, Medical Psychology and Psychotherapy, Medical Sociology and Rehabilitation Sciences<br>Sanderman, Robbert; University of Groningen, University Medical Center Groningen, Department of Health Psychology; University of Twente, Department of Psychology, Health and Technology<br>Schultz, Konrad; Klinik Bad Reichenhall, Center for Rehabilitation, Pulmonology and Orthopedics<br>Kocks, Janwillem; University of Groningen, University Medical Center Groningen, Department of General Practice and Elderly Care Medicine; University of Groningen, University Medical Center Groningen, Groningen Research Institute for Asthma and COPD (GRIAC)<br>van der Molen, Thys; University of Groningen, University Medical Center Groningen, Department of General Practice and Elderly Care Medicine; University of Groningen, University Medical Center Groningen, Groningen Research Institute for Asthma and COPD (GRIAC) |
| <b>Primary Subject Heading</b>: | Respiratory medicine |
| Secondary Subject Heading: | Epidemiology, Research methods, Respiratory medicine |
| Keywords: | Chronic Obstructive Pulmonary Disease (COPD), Health-Related Quality of Life (HRQoL), Health Status Responsiveness, Pulmonary Rehabilitation (PR), Routine Clinical Practice (RCP), Minimal Clinically Important Difference (MCID) |

SCHOLARONE™
Manuscripts

# Thresholds for Clinically Important Deterioration versus Improvement in COPD Health Status: Results from a Randomized Controlled Trial in Pulmonary Rehabilitation and an Observational Study during Routine Clinical Practice

Harma Alma[ab], MD MSc; Corina de Jong, PhD[ab]; Danijel Jelusic[c], MD; Michael Wittmann[c], MD; Michael Schuler[d], PhD; Prof. dr. Robbert Sanderman[ef], PhD; Konrad Schultz, MD[c]; Janwillem Kocks[ab], MD, PhD; Prof. dr. Thys van der Molen[ab], MD, PhD

[a] University of Groningen, University Medical Center Groningen, Department of General Practice and Elderly Care Medicine, Groningen, The Netherlands

[b] University of Groningen, University Medical Center Groningen, Groningen Research Institute for Asthma and COPD (GRIAC), Groningen, The Netherlands

[c] Klinik Bad Reichenhall, Center for Rehabilitation, Pulmonology and Orthopedics, Bad Reichenhall, Germany

[d] University of Wuerzburg, Department of Medical Psychology and Psychotherapy, Medical Sociology and Rehabilitation Sciences, Wuerzburg, Germany

[e] University of Groningen, University Medical Center Groningen, Department of Health Psychology, Groningen, The Netherlands

[f] University of Twente, Department of Psychology, Health and Technology, Enschede, The Netherlands

| | |
|---|---|
| Harma Alma | h.j.alma@umcg.nl |
| Corina de Jong | c.de.jong02@umcg.nl |
| Danijel Jelusic | djelusic87@gmail.com |
| Michael Wittmann | michael.wittmann@klinik-bad-reichenhall.de |
| Michael Schuler | m.schuler@uni-wuerzburg.de |
| Robbert Sanderman | r.sanderman@umcg.nl |
| Konrad Schultz | konrad.schultz@klinik-bad-reichenhall.de |
| Janwillem Kocks | j.w.h.kocks@umcg.nl |
| Thys van der Molen | t.van.der.molen@umcg.nl |

Corresponding author:

Mrs. H.J. Alma, MD MSc
University of Groningen, University Medical Center Groningen
Department of General Practice and Elderly Care Medicine
HPC FA21, Postbox 196
NL-9700 AD Groningen
T: +31 6 27137024
E: h.j.alma@umcg.nl

Total word count manuscript: 3945 words (Abstract 298 words)

For peer review only



For peer review only - http://bmjopen.bmj.com/site/about/guidelines.xhtml

## Abstract

**Objectives:**

Chronic Obstructive Pulmonary Disease (COPD) is a progressive disease. Preventing deterioration of health status is therefore an important therapy goal. (Minimal) Clinically Important Differences ((M)CIDs) are used to interpret changes observed. It remains unclear whether (M)CIDs are similar for both deterioration and improvement in health status. This study investigated and compared these clinical thresholds for three widely-used questionnaires.

**Methods:**

*Design and setting:* Data were retrospectively analysed from an in-house 3-week Pulmonary Rehabilitation (PR) randomized controlled trial in the German Klinik Bad Reichenhall (study one); and observational research in Dutch primary and secondary Routine Clinical Practice (RCP) (study two).

*Participants:* COPD patients aged ≥18 years (study one) and aged ≥40 years (study two) without respiratory co-morbidities were included for analysis.

*Primary outcomes:* The COPD Assessment Test (CAT), Clinical COPD Questionnaire (CCQ) and St. George's Respiratory Questionnaire (SGRQ) were completed at baseline, three, six, and 12 months. A Global Rating of Change scale (GRC) was added at follow-up. Anchor- and distribution-based methods were used to determine clinically relevant thresholds.

**Results:**

In total, 451 patients were included from PR and 207 from RCP. MCIDs for deterioration ranged 1.30 to 4.21 (CAT), 0.19 to 0.66 (CCQ), 2.75 to 7.53 (SGRQ). MCIDs for improvement ranged -3.78 to -1.53 (CAT), -0.50 to -0.19 (CCQ), and -9.20 to -2.76 (SGRQ). Thresholds for moderate improvement versus deterioration ranged -5.02 to -3.29 vs. 3.89 to 8.14 (CAT), -0.90 to -0.72 vs. 0.42 to 1.23 (CCQ), -15.85 to -13.63 vs. 7.46 to 9.30 (SGRQ).

**Conclusions:**

MCID ranges for improvement and deterioration on the CAT, CCQ and SGRQ were somewhat similar. However, estimates for moderate and large change varied and were inconsistent. Thresholds differed between study settings.



**Trial registration:**

RIMTCORE trial (#DRKS00004609; Ethik-Kommission der Bayerischen Landesärztekammer #12107) and MCID Study (University Medical Center Groningen (UMCG) Research Register (#201500447)).




For peer review only - http://bmjopen.bmj.com/site/about/guidelines.xhtml

## Article Summary

**Strengths:**

- Our study is the first dedicated investigation of (Minimal) Clinically Important Differences ((M)CIDs) for deterioration on Chronic Obstructive Pulmonary Disease (COPD) health status tools in comparison to thresholds for improvement.
- Our study used a combination of anchor- and distribution-based methods to determine clinically relevant thresholds for both deterioration and improvement.
- Our study investigated clinically relevant thresholds in two different study settings - Pulmonary Rehabilitation (PR) and Routine Clinical Practice (RCP) - by using data from various follow-up periods to minimize the possible impact of the recall period.

**Limitations:**

- Our study included a limited number of patients with deterioration after PR intervention and during RCP, and a limited number of patients indicating moderate and large changes in health status.
- Our study resulted in broad ranges and wide confidence intervals for the (M)CIDs of COPD health status tools, requiring possibly larger sample sizes for more accuracy.

For peer review only

## Declarations

## Funding

The Routine Inspiratory Muscle Training within COPD Rehabilitation (RIMTCORE) trial (#DRKS00004609), including patients in Pulmonary Rehabilitation (PR), was funded by the *Deutsche Rentenversicherung*. The Dutch observational study on Chronic Obstructive Pulmonary Disease (COPD) health status in Routine Clinical Practice (RCP) as well as the current combined retrospective analysis of both prospective studies received financial support from the Junior Scientific Masterclass (JSM) as part of the University of Groningen.

## Competing interests

H.J. Alma, C. de Jong, D. Jelusic, M. Wittmann, M. Schuler and R. Sanderman have nothing to disclose. J.W.H. Kocks reports personal fees from Novartis; research grants and personal fees from Boehringer Ingelheim; research grants and personal fees from GlaxoSmithKline (GSK); research grants from Stichting Zorgdraad; personal fees from International Primary Care Respiratory Group (IPCRG); personal fees from Springer Media; and travel arrangements from Chiesi BV, GSK, and IPCRG, all outside the submitted work. K. Schultz received lecture fees from Boehringer, AstraZeneca, Berlin Chemie, Novartis, Chiesi, Mundipharma, Takeda, GSK and MSD, all outside the submitted work. T. van der Molen reports personal reimbursements from GSK, TEVA, Astra Zeneca, Boehringer Ingelheim and study grants from Astra Zeneca and GSK. After this study was terminated, he became employee of GSK. None of these stated conflicts of interest are linked to the current manuscript. T. van der Molen developed the Clinical COPD Questionnaire (CCQ) and holds the copyright.

## Authors' contributions

KS, MW, DJ and MS planned the RIMTCORE study design and were responsible for data collection. HA, CdJ, RS and TvdM designed the Dutch observational study on COPD health status in Routine Clinical Practice as well as the current retrospective analysis of both prospective studies. HA and CdJ performed the statistical analysis. HA wrote the first draft, while CdJ, JK, RS and TvdM actively participated in the review process. RS and TvdM supervised and participated in different steps of the study, as well as in writing. All authors participated in and approved of the final version of the manuscript

## Consent of publication

All authors participated in various steps in the study, edited the manuscript and gave their approval for submission.



For peer review only - http://bmjopen.bmj.com/site/about/guidelines.xhtml

For peer review only

## Ethics approval and consent to participate

This study is a secondary retrospective analysis of a subsample from the Routine Inspiratory Muscle Training within COPD Rehabilitation (RIMTCORE) real-life randomized controlled trial (#DRKS00004609) in the Klinik Bad Reichenhall, Center for Rehabilitation, Pulmonology and Orthopaedics in Germany; and a primary analysis of all patients participating in the Dutch observational trial (MCID study) on COPD health status in routine clinical practice (UMCG trial #201500447). All patients in both studies signed informed consent upon participation. The RIMTCORE trial has been approved by the Ethik-Kommission der Bayerischen Landesärztekammer and registered in the German Clinical Trial Register. The MCID study has been registered at the University Medical Center Groningen (UMCG) Research Register and evaluated by its Medical Ethical Committee.

## Data Sharing Statement

The data that support the findings of this study are not publicly available. Participating patients in the RIMTCORE trial have only agreed upon availability of their data to the Klinik Bad Reichenhall, their scientific partners in the data analysis and the Committee of the Bavarian State Chamber of Labor in Munich. Participating patients in the MCID study only agreed upon availability of their data to the University Medical Center Groningen (UMCG) and their scientific partners in the data analysis.

## Acknowledgements

We are grateful to the Junior Scientific Masterclass of the University of Groningen, who financially supported the research position of the first author. We would also like to acknowledge all participating patients in both the RIMTCORE trial and the MCID study.

For peer review only

**Key Words**

Chronic Obstructive Pulmonary Disease (COPD)

Health-Related Quality of Life (HRQoL)

Health Status Responsiveness

Pulmonary Rehabilitation (PR)

Routine Clinical Practice (RCP)

Minimal Clinically Important Difference (MCID)

For peer review only



For peer review only - http://bmjopen.bmj.com/site/about/guidelines.xhtml

# Article manuscript

## Introduction

The use of health status questionnaires is recommended by the Global Initiative for Chronic Obstructive Lung Disease (GOLD) for the assessment, evaluation and management of patients with Chronic Obstructive Pulmonary Disease (COPD) [1]. The COPD Assessment Test (CAT) [2], the Clinical COPD Questionnaire (CCQ) [3], and the St. George's Respiratory Questionnaire (SGRQ) [4] are frequently used patient-reported health status tools important for clinical practice and scientific research [5], especially since the burden of COPD is high worldwide [6-7].

Various studies have examined clinically relevant thresholds for change on the CAT, CCQ and SGRQ in order to be able to evaluate and interpret treatment effects [8-18]. The Minimal Clinically Important Difference (MCID) is a parameter that quantifies this threshold. It has been defined as *"the smallest difference in score, which patients perceive as beneficial and which would mandate a change in the patient's management"* [19]. MCIDs are particularly interesting for health status questionnaires, where a change in its score is not intuitively meaningful. Change exceeding the level of the MCID can be considered clinically relevant, thus justifying therapy and help developing guidelines. It is pivotal that clinically relevant thresholds for change on a health status tool are rigorously studied and analysed carefully.

Most clinical studies that determine the MCID of Patient-Reported Outcomes (PROs) are executed in the context of an intervention such as pharmacotherapy or Pulmonary Rehabilitation (PR). This usually results in an improvement in the patients' Health-Related Quality of Life (HRQoL). MCIDs for improvement have thus been investigated upon; however there is a lack of evidence for the MCIDs for deterioration [20]. It remains unclear and debated upon to what extent clinically relevant thresholds for improvement should be similar to those for deterioration [21-24]. Certain studies outside the field of COPD have analysed the MCIDs of PROs and found evidence that values for improvement differed from deterioration [25-29]. On the other hand, there is also evidence that thresholds might be similar [30]. Interpreting worsening of HRQoL is of major importance, since one needs to differentiate between real worsening of patients' status and random variations. Furthermore, the effects of therapy may also halt further deterioration especially for a progressive chronic disease like COPD. So, no relevant worsening or a reduction in clinically relevant deterioration over time might also be considered a success of therapy and in clinical trials [31].



For peer review only
For peer review only - http://bmjopen.bmj.com/site/about/guidelines.xhtml

In COPD health status, the estimated MCID for the CAT score is 2.00 to 3.00 units [11-15, 20]; for the CCQ score 0.40 to 0.50 units [8-13, 20]; and for the SGRQ score 4.00 to 8.00 units [12, 16-18, 20]. This is valid for improvement only, as there were too few patients with deterioration to investigate. There are currently no studies that specifically investigate clinically relevant thresholds for deterioration on these PROs. It is however worrying that up to date, multiple studies include the MCIDs of these COPD health status instruments for improvement to interpret deterioration in clinical trials [32-34] This study therefore aimed to determine and compare clinically relevant thresholds for deterioration and improvement on the COPD health status questionnaires CAT, CCQ and SGRQ in both a PR and Routine Clinical Practice (RCP) setting.

For peer review only



For peer review only - http://bmjopen.bmj.com/site/about/guidelines.xhtml

# Patients and methods

## Study subjects

This study was a retrospective analysis of data obtained from two prospective clinical trials. Study one was a secondary analysis of a subsample from the Routine Inspiratory Muscle Training within COPD Rehabilitation (RIMTCORE) real-life randomized controlled trial in the Klinik Bad Reichenhall, Center for Rehabilitation, Pulmonology and Orthopedics in Germany [12, 35]. Patients were recruited upon arrival in the clinic between February 2013 and July 2014. Participants were included if they had COPD category GOLD II-IV, were aged ≥18 years and gave informed consent [12, 35]. Exclusion criteria were the presence of other respiratory co-morbidities (e.g. bronchiectasis, asthma, history of bronchial carcinoma, sarcoidosis, tuberculosis); or alpha-1-antitrypsin deficiency.

Study two (MCID study) was an observational trial of COPD patients GOLD I-IV aged ≥40 years without other respiratory co-morbidities or alpha-1-antitrypsin deficiency. Patients were recruited from Dutch primary and secondary Routine Clinical Practice (RCP) between September 2015 and September 2016. Patients were approached via multiple general practices, hospitals and the Dutch patient lung federation. The study was evaluated by the Medical Ethical Committee of the University Medical Center Groningen (UMCG), the Netherlands. All patients provided written informed consent.

## Patient and public involvement

In both studies, patients and the public have not actively been involved during the design of the study nor the assessment of the burden. Summary results are disseminated to participating patients after completion.

## Study design and data collection

Patients in study one participated in an intensive 3-week full-day inpatient PR program tailored to the patient's individual needs. Details have been presented previously [12, 35]. Patient descriptives and post-bronchodilator spirometry were collected at baseline and discharge in the clinic. Patients in study two received routine care from their physician according to national treatment guidelines. Evaluation of health status over a 12-months period was the primary measurement outcome. Patient descriptives and spirometry data were obtained at baseline. Spirometry results were obtained via the including physician after approval of the participant.

Primary outcomes selected from both prospective studies for this retrospective analysis were the CAT (no recall period), CCQ (weekly version) and SGRQ (monthly version). In study one, these questionnaires were collected at



For peer review only - http://bmjopen.bmj.com/site/about/guidelines.xhtml

baseline, at PR discharge and during follow-up at three, six, nine and 12 months. Baseline and discharge measurements were taken in the clinic, where patients were blinded to their baseline scores. Follow-up questionnaires were sent by mail. In study two, all questionnaires were sent by mail and scored at home at baseline, three, six and 12 months. For this retrospective analysis baseline and follow-up scores at three, six and 12 months were included, to allow for sufficient time for deterioration in HRQoL, to include various time periods of measurement, and to allow for comparison between both study settings.

The CAT is an eight-item one-dimensional scale with item scores ranging 0-5 (0: no impairment, 5: maximum impairment) and a total score summing up to a maximum of 40 [2]. The CCQ consists of ten items scoring 0-6 (0: no impairment, 6: maximum impairment) [3]. The items cover the domains symptoms (four items), functional status (four items) and mental status (two items). Total and domain scores on the CCQ derive from adding up relevant item scores and dividing this by the number of items. The SGRQ has 50 items classified into the domains symptoms (eight items), activities (16 items) and impact (26 items) [4]. Domain and total SGRQ scores can range from 0-100 (0: no impairment, 100: maximum impairment). A 15-point Likert scale anchor question (Global Rating of Change GRC) was scored retrospectively by the patient at each follow-up visit in both datasets. The GRC required patients to assess their COPD health status compared to baseline. The answers were marked on a scale from -7 to +7, ranging from *very much worse* to *very much better* and zero equalling *no change* [36-37].

**Study methods**

All change scores for the total scores of the CAT, CCQ and SGRQ were calculated as the difference between baseline and the respective follow-up visit (three, six and 12 months). Negative change on all questionnaires represented improvement, positive change deterioration. First, in the anchor-based approach, changes on the health status instruments were classified using the corresponding score on the GRC. Scores of 0 and ±1 on the GRC indicated *no change*; scores of ±2 and ±3 represented a *minimal improvement/deterioration*; scores of ±4 and ±5 were summarized as a *moderate improvement/deterioration*; and scores of ±6 and ±7 indicated a *large improvement/deterioration* [36-37]. MCID estimates for both improvement and deterioration on the CAT, CCQ and SGRQ were calculated as the mean change scores including 95% Confidence Interval (95%CI) of those patients indicating a minimal improvement/deterioration (±2 and ±3) on the GRC for each follow-up visit, verifying normality of distribution. Mean estimates including 95%CI were determined in a similar way for patients indicating no change (GRC 0 and ±1), moderate change (GRC ±4 and ±5) and large change (GRC ±6 and ±7). Second, the distribution-based method half Standard Deviation (0.5 SD) of the change score was calculated for improved and deteriorating health status patients at respective follow-up visits [38].



For peer review only - http://bmjopen.bmj.com/site/about/guidelines.xhtml

**Data analysis**

Data analysis was performed using SPSS 24.0 (IBM, Chicago, USA). Descriptives were evaluated at baseline for either frequencies with percentages (%), mean with Standard Deviation (SD) or median with range. This was depending on the variable characteristics and/or normality of distribution. Health status data on the CCQ, CAT and SGRQ were evaluated at baseline (T0), three months (T3), six months (T6) and after 12 months (T12). Normality of distribution was verified using skewness and kurtosis. Values between -1 and +1 were considered indicative for normality. Data were checked for floor- and ceiling effects defined as over 15% of patients scoring in the lowest and highest 10% of the maximum scale range [39]. Mean and standard deviations (or median and range) were calculated at each measurement moment for all patients, as well as specifically for patients with improved and deteriorated health status change scores. Baseline scores were compared between improving and deteriorating patients, and tested using independent t-tests after verifying normality of distribution. Baseline scores were compared between both datasets (PR vs. RCP) using independent t-tests, Man-Whitney U tests or Chi-Square tests depending on the variable characteristic and/or normality of distribution. Health status change scores were all calculated in comparison to baseline. Follow-up scores were compared with baseline to test for significance of change using paired t-tests verifying normality of distribution.

In order to determine the clinically relevant thresholds for change, first correlations between the GRC and the CCQ, CAT and SGRQ were assessed using Pearson or Spearman correlation coefficients depending on normality of distribution. Correlations needed to be ≥0.30 (preferably ≥0.50) to be eligible as anchor [22]. Correlations were not only assessed between GRC and questionnaire change scores, but also between GRC, baseline and follow-up questionnaire score to assess for a possible *response shift*. Next, participants were categorized according to their GRC score at each follow-up. Mean changes (95%CI) for each respective category were determined to define thresholds for clinically relevant change. Significance of change for each GRC class at the respective follow-up visit was compared to baseline and assessed with paired t-tests verifying normality of the data. Last, the 0.5SD of the change score was determined for patients with improved and deteriorating health status change scores separately at each follow-up. Thresholds were compared between both study settings (PR vs. RCP).

An absolute overall weighted mean MCID estimate for both improvement and deterioration was calculated at the end by multiplying the number of observations (n) at each follow-up visit times the MCID estimate for that period. The sum was divided by the total number of observations. Anchor-based and distribution-based approaches had similar weights. Estimates for improvement and deterioration were compared visually in a plot.



For peer review only - http://bmjopen.bmj.com/site/about/guidelines.xhtml

# Results

## Patient characteristics

Study one included 451 patients with completed baseline data (Table 1) [12, 35]. During follow-up 355 patients (78.7%) had completed data at T3; 319 patients (70.7%) at T6; and 309 patients (68.5%) at T12. During the 12-months follow-up eight patients passed away; 41 dropped out at own request; and a varying number of non-response was present. Study two included 207 patients with full baseline data (Table 1), of whom 201 (97.1%) completed T3, 186 (89.9%) T6 and 177 (85.6%) T12. Four patients died; 12 patients discontinued at own request; and a various number of non-response was present.

There were no significant baseline differences between completers and non-completers of the 12-months follow-up in both studies, except that significantly more females (28.4%) compared with men (10.0%) did not complete the follow-up during RCP. Significant differences in age, Forced Expiratory Volume in one second percentage predicted (FEV1%pred) and health status were observed between both studies (Table 1).

*Table 1: Baseline patient characteristics*

| | **Study 1: PR** | **Study 2: RCP** | **Significance testing** |
|---|---|---|---|
| N (number of patients) | 451 | 207 | - |
| Age (years)[a] | 57.87 ± 6.56 | 66.69 ± 7.91 | *P < 0.001** |
| Gender (male)[b] | 293 (65.0) | 121 (58.5) | *P = 0.507* |
| FEV1%pred[a] | 50.40 ± 15.11 | 57.06 ± 21.96 | *P = 0.001** |
| GOLD I[b] | - | 35 (17.4) | *P = 0.199* |
| GOLD II | 227 (50.3) | 80 (39.8) | |
| GOLD III | 176 (39.0) | 61 (30.3) | |
| GOLD IV | 48 (10.6) | 25 (12.4) | |
| Smoking pack years[a] | 40 (30-50) | 37.5 (22.50-51.25) | *P = 0.081* |
| CAT Total[a] | 20.23 ± 7.33 | 18.32 ± 7.22 | *P = 0.002** |
| CCQ Total[a] | 2.86 ± 1.17 | 2.12 ± 1.02 | *P < 0.001** |
| CCQ Symptoms[a] | 2.87 ± 1.24 | 2.48 ± 1.03 | *P < 0.001** |
| CCQ Functional Status[a] | 2.86 ± 1.34 | 2.28 ± 1.40 | *P < 0.001** |
| CCQ Mental Status[a] | 2.86 ± 1.74 | 1 (0-1.50) | *P < 0.001** |
| SGRQ Total[a] | 50.69 ± 17.33 | 42.88 ± 19.16 | *P < 0.001** |
| SGRQ Symptoms[a] | 63.66 ± 21.77 | 48.04 ± 24.16 | *P < 0.001** |
| SGRQ Activities[a] | 63.58 ± 19.82 | 61.48 ± 21.10 | *P = 0.259* |
| SGRQ Impact[a] | 39.21 ± 18.81 | 30.52 ± 19.73 | *P < 0.001** |
| mMRC[a] | 2 (2-4) | 1 (1-2) | *P < 0.001** |

[a] Data were expressed as mean ± standard deviation or median (IQR).
[b] Data were expressed as frequencies (% of total).

* Significance testing at level $p < 0.05$ using unpaired T-tests, Man Whitney-U tests or Chi Square tests.

*Abbreviations:* CAT, COPD Assessment Test; CCQ, Clinical COPD Questionnaire; FEV1%pred, Forced Expiratory Volume in one Second % predicted; GOLD, Global Initiative for Chronic Obstructive Lung Disease; mMRC, modified Medical Research Council Dyspnea Scale; N, Number of Patients; PR, Pulmonary Rehabilitation; RCP, Routine Clinical Practice; SGRQ, St. George's Respiratory Questionnaire



For peer review only
For peer review only - http://bmjopen.bmj.com/site/about/guidelines.xhtml

***Health status scores for improvement and deterioration***

In study one and two, CAT, CCQ and SGRQ total were normally distributed at baseline and follow-up. Completed pairs of change scores (follow-up vs. baseline) were included (pair-wise deletion). Floor- and ceiling effects were negligible. Mean health status baseline scores were significantly different for PR and RCP (Table 1). Overall, 58-59% of patients had *improved* health status scores (negative change) at T12 after PR; compared with 44-46% during RCP (Table 2). After PR mean changes observed on the CAT questionnaire at T12 were -5.45±4.66 for improvers and 5.47±4.22 for patients who deteriorated; on the CCQ questionnaire -0.87±0.72 for improvement and 0.83±0.62 for deterioration; and on the SGRQ questionnaire -13.83±10.43 for improvers and 10.19±8.94 for (Table 2). These estimates were in RCP for the CAT -4.53±3.15 for improvement and 3.88±2.59 for deterioration; for the CCQ -0.54±0.54 for improvement and 0.51±0.39 for deterioration; and for the SGRQ -7.74±9.51 for improvement on and 8.46±7.06 for deterioration (Table 2).

There were no baseline differences in terms of age, gender and GOLD classification between improved health status patients and those who deteriorated at T12 in both studies. Patients with a worse (read higher) CAT, CCQ or SGRQ baseline score prior to PR had significantly more improved health status after one year. Patients, who improved during RCP, had a significantly higher baseline FEV1%pred.



For peer review only - http://bmjopen.bmj.com/site/about/guidelines.xhtml

*Table 2: Health status baseline and change scores for all, improved and deteriorated patients during PR and Routine Clinical Practice (RCP)*

| | Change after 3 months (T3) | N | Change after 6 months (T6) | N | Change after 12 months (T12) | N |
|---|---|---|---|---|---|---|
| **CAT** | | | | | | |
| *All patients PR* | -1.44* (-2.16 to -0.71) | 354 | -0.91* (-1.66 to -0.16) | 319 | -0.89* (-1.68 to -0.11) | 309 |
| *Improvement PR* | -5.45±4.57 | 227 (64.1) | -5.49±4.33 | 184 (57.7) | -5.45±4.66 | 180 (58.3) |
| *Deterioration PR* | 5.75±4.20 | 127 (35.9) | 5.33±4.10 | 135 (42.3) | 5.47±4.22 | 129 (41.7) |
| *All patients RCP* | 0.30 (-0.42 to +1.02) | 201 | 0.18 (-0.53 to +0.90) | 186 | 0.14 (-0.59 to +0.87) | 177 |
| *Improvement RCP* | -4.04±3.33 | 102 (50.7) | -4.64±3.05 | 81 (43.5) | -4.53±3.15 | 79 (44.6) |
| *Deterioration RCP* | 4.23±3.66 | 83 (41.3) | 3.76±2.88 | 91 (48.9) | 3.88±2.59 | 86 (48.6) |
| *No change RCP* | - | 16 (8.0) | - | 14 (7.5) | - | 12 (6.8) |
| **CCQ Total** | | | | | | |
| *All patients PR* | -0.26* (-0.37 to -0.15) | 355 | -0.11 (-0.23 to +0.01) | 319 | -0.16* (-0.28 to -0.04) | 309 |
| *Improvement PR* | -0.88±0.71 | 225 (63.4) | -0.84±0.68 | 181 (56.7) | -0.87±0.72 | 180 (58.3) |
| *Deterioration PR* | 0.82±0.68 | 130 (36.6) | 0.84±0.67 | 138 (43.3) | 0.83±0.62 | 129 (41.7) |
| *All patients RCP* | 0.00 (-0.09 to +0.08) | 200 | 0.00 (-0.10 to +0.10) | 185 | -0.02 (-0.12 to +0.09) | 174 |
| *Improvement RCP* | -0.45±0.37 | 96 (48.0) | -0.52±0.51 | 87 (47.0) | -0.54±0.54 | 77 (44.3) |
| *Deterioration RCP* | 0.50±0.38 | 89 (44.5) | 0.56±0.46 | 80 (43.2) | 0.51±0.39 | 88 (50.6) |
| *No change RCP* | - | 15 (7.5) | - | 18 (9.7) | - | 9 (5.2) |
| **SGRQ Total** | | | | | | |
| *All patients PR* | -5.35* (-6.92 to -3.78) | 350 | -4.85* (-6.47 to -3.23) | 312 | -3.94* (-5.67 to -2.21) | 306 |
| *Improvement PR* | -13.11±9.65 | 237 (67.7) | -13.51±9.88 | 193 (61.9) | -13.83±10.43 | 180 (58.8) |
| *Deterioration PR* | 10.93±10.18 | 113 (32.3) | 8.19±8.92 | 119 (38.1) | 10.19±8.94 | 126 (41.2) |
| *All patients RCP* | -0.52 (-1.77 to +0.73) | 198 | -1.34 (-2.76 to +0.07) | 184 | -0.87 (-2.60 to +0.86) | 174 |
| *Improvement RCP* | -6.61±5.58 | 97 (49.0) | -7.91±5.52 | 75 (40.8) | -7.74±9.51 | 81 (46.6) |
| *Deterioration RCP* | 7.36±5.49 | 101 (51.0) | 7.78±6.18 | 108 (58.7) | 8.46±7.06 | 92 (52.9) |
| *No change RCP* | - | 0 | - | 1 (0.5) | - | 1 (0.6) |

Change was calculated compared with baseline. Negative change represents improvement for CAT, CCQ and SGRQ. Change scores for all patients reported as mean (95%CI). Change scores for improvement and deterioration are presented as mean ± SD.

*Paired t-tests were significant at level $p<0.05$ testing follow-up versus baseline measurements.

*Abbreviations:* 95%CI, 95% Confidence Interval; CAT, COPD Assessment Test; CCQ, Clinical COPD Questionnaire; N, Number of patients; PR, Pulmonary Rehabilitation; RCP, Routine Clinical Practice; SD, Standard Deviation; SGRQ, St. George's Respiratory Questionnaire; T3, Three months follow-up; T6, Six months follow-up; T12, 12 months follow-up.

For peer review only



For peer review only - http://bmjopen.bmj.com/site/about/guidelines.xhtml

***Clinically Important Improvement versus Deterioration***

Significant correlations between the health status change scores and the GRC ranged respectively for study one -0.33 to -0.41 (CAT), -0.42 to -0.47 (CCQ), and -0.48 to -0.54 (SGRQ) (Table 3). These ranges were for study two respectively -0.29 to -0.37, -0.38 to -0.48, and -0.35 to -0.44. GRC scores had stronger correlations with the respective follow-up health status score compared with baseline and change scores for both studies.

*Table 3: Correlations between health status (change) scores and the Global Rating of Change (GRC)*

| | **GRC T3-T0** | | **GRC T6-T0** | | **GRC T12-T0** | |
|---|---|---|---|---|---|---|
| | *PR (N=355)* | *RCP (N=201)* | *PR (N=319)* | *RCP (N=186)* | *PR (N=309)* | *RCP (N=177)* |
| CAT Change Score | -0.33* | -0.29* | -0.40* | -0.30* | -0.41* | -0.37* |
| CAT T0 | -0.31* | -0.11 | -0.25* | -0.22* | -0.34* | -0.22* |
| CAT T3 | **-0.56*** | -0.31* | **-0.50*** | -0.31* | **-0.50*** | -0.33* |
| CAT T6 | - | - | **-0.55*** | -0.40* | **-0.59*** | -0.34* |
| CAT T12 | - | - | - | - | **-0.64*** | -0.48* |
| | | | | | | |
| CCQ Change Score | -0.42* | -0.38* | -0.44* | -0.40* | -0.47* | -0.48* |
| CCQ T0 | -0.26* | -0.14* | -0.19* | -0.22* | -0.29* | -0.23* |
| CCQ T3 | **-0.61*** | -0.35* | **-0.52*** | -0.26* | **-0.54*** | -0.33* |
| CCQ T6 | - | - | **-0.56*** | -0.43* | **-0.59*** | -0.39* |
| CCQ T12 | - | - | - | - | **-0.66*** | **-0.51*** |
| | | | | | | |
| SGRQ Change Score | -0.48* | -0.35* | **-0.51*** | -0.33* | **-0.54*** | -0.44* |
| SGRQ T0 | -0.28* | -0.13 | -0.24* | -0.20* | -0.32* | -0.22* |
| SGRQ T3 | **-0.62*** | -0.29* | **-0.56*** | -0.25* | **-0.58*** | -0.28* |
| SGRQ T6 | - | - | **-0.61*** | -0.35* | **-0.62*** | -0.35* |
| SGRQ T12 | - | - | - | - | **-0.69*** | **-0.51*** |

Data reported as Pearson or Spearman correlation coefficients between the health status (change) scores and the GRC anchor question. Correlations ≥0.50 are highlighted bold.

* Correlations are significant at level $p < 0.05$.

*Abbreviations:* CAT, COPD Assessment Test; CCQ, Clinical COPD Questionnaire; GRC, Global Rating of Change; N, Number of Patients; PR, Pulmonary Rehabilitation; RCP, Routine Clinical Practice; SGRQ, St. George's Respiratory Questionnaire; T0, Baseline measurement; T3, Three months follow-up; T6, Six months follow-up; T12, 12 months follow-up.

Tables 4-6 and figures 1-3 present the clinically relevant thresholds for minimal, moderate and large changes on the CAT, CCQ and SGRQ during PR and Routine Clinical Practice (RCP). On the CAT anchor- and distribution-based estimates ranged -2.80 to -2.17 (weighted mean -2.51) for minimal improvement and 2.05 to 4.21 for minimal deterioration (weighted mean 2.76) during PR (Table 4, Figure 1). These ranges were respectively -3.78 to -1.53 (weighted mean -2.49) and 1.30 to 1.97 (weighted mean 1.65) during RCP. On the CCQ minimal clinically important improvements were determined at -0.50 to -0.34 (weighted mean -0.40) for PR and -0.44 to -0.19 (weighted mean -0.33) for RCP (Table 5, Figure 2). These thresholds for deterioration were 0.31 to 0.66 (weighted mean 0.43) during PR and 0.19 to 0.46 (weighted mean 0.30) during RCP. On the SGRQ estimates ranged -9.20 to -4.83 (weighted mean -6.74) for minimal improvement and 4.46 to 7.52 for minimal deterioration (weighted



For peer review only - http://bmjopen.bmj.com/site/about/guidelines.xhtml

mean 5.31) during PR (Table 6, Figure 3). These ranges were respectively -4.76 to -2.76 (weighted mean -4.06) and 2.75 to 7.53 (weighted mean 4.78) during RCP.

*Table 4: Estimates for clinically relevant thresholds for improvement and deterioration on the CAT*

| **CAT** | **T3-T0** | | **T6-T0** | | **T12-T0** | | **Weighted threshold** | |
|---|---|---|---|---|---|---|---|---|
| *Change* | *Improvement* | *Deterioration* | *Improvement* | *Deterioration* | *Improvement* | *Deterioration* | *Improvement* | *Deterioration* |
| | | | | | | | | |
| **Minimal change** | | | | | | | | |
| N Anchor-based PR | 107 | 36 | 96 | 42 | 88 | 43 | 291 | 121 |
| Anchor-based PR | -2.74 | 2.71 | -2.73 | 3.21 | -2.80 | 4.21 | -2.75 | 3.42 |
| N Anchor-based RCP | 12 | 27 | 14 | 36 | 18 | 46 | 32 | 82 |
| Anchor-based RCP | - | - | -2.86 | 1.97 | -3.78 | 1.63 | -3.38 | 1.78 |
| | | | | | | | | |
| N distribution-based PR | 227 | 127 | 184 | 135 | 180 | 129 | 591 | 391 |
| Distribution-based PR | -2.29 | 2.10 | -2.17 | 2.05 | -2.33 | 2.11 | -2.26 | 2.09 |
| N distribution-based RCP | 102 | 83 | 81 | 91 | 79 | 86 | 262 | 260 |
| Distribution-based RCP | -1.67 | 1.83 | -1.53 | 1.44 | -1.58 | 1.30 | -1.60 | 1.52 |
| | | | | | | | | |
| **Moderate change** | | | | | | | | |
| N Anchor-based PR | 51 | 9 | 45 | 7 | 37 | 10 | 133 | 17 |
| Anchor-based PR | -5.02 | - | -3.29 | 8.14 | -4.27 | 6.30 | -4.23 | 7.06 |
| N Anchor-based RCP | 5 | 8 | 12 | 9 | 5 | 9 | - | 9 |
| Anchor-based RCP | - | - | - | - | - | 3.89 | - | 3.89 |
| | | | | | | | | |
| **Large change** | | | | | | | | |
| N Anchor-based PR | 16 | 3 | 12 | 2 | 14 | 3 | 42 | - |
| Anchor-based PR | -4.19 | - | -7.00 | - | -6.07 | - | -5.62 | - |
| N Anchor-based RCP | 4 | 3 | 0 | 2 | 9 | 4 | 13 | 4 |
| Anchor-based RCP | -6.00 | - | - | - | -4.22 | 5.75 | -4.77 | 5.75 |
| | | | | | | | | |
| **No change** | | | | | | | | |
| N Anchor-based PR | 133 | | 115 | | 114 | | 362 | |
| Anchor-based PR | 0.03 | | -0.01 | | -0.33 | | -0.10 | |
| N Anchor-based RCP | 141 | | 113 | | 83 | | 337 | |
| Anchor-based RCP | -0.16 | | -0.54 | | -0.47 | | -0.36 | |

Data reported as clinically relevant threshold or N. Negative change represents improvement for all health status instruments. Paired t-tests were applied with significance level at $p < 0.05$. Non- significant results were excluded, except for the "*No change*" group.

*Abbreviations:* CAT, COPD Assessment Test; GRC, Global Rating of Change; N, Number of Patients; PR, Pulmonary Rehabilitation; RCP, Routine Clinical Practice; T0, Baseline measurement; T3, Three months follow-up; T6, Six months follow-up; T12, 12 months follow-up.

For peer review only



For peer review only - http://bmjopen.bmj.com/site/about/guidelines.xhtml

*Table 5: Estimates for clinically relevant thresholds for improvement and deterioration on the CCQ*

| **CCQ** | **T3-T0** | | **T6-T0** | | **T12-T0** | | **Weighted threshold** | |
|---|---|---|---|---|---|---|---|---|
| *Change* | *Improvement* | *Deterioration* | *Improvement* | *Deterioration* | *Improvement* | *Deterioration* | *Improvement* | *Deterioration* |
| | | | | | | | | |
| **Minimal change** | | | | | | | | |
| N Anchor-based PR | 107 | 36 | 96 | 42 | 88 | 43 | 291 | 121 |
| Anchor-based PR | -0.44 | 0.42 | -0.42 | 0.48 | -0.50 | 0.66 | -0.45 | 0.53 |
| N Anchor-based RCP | 12 | 27 | 14 | 36 | 18 | 46 | 32 | 82 |
| Anchor-based RCP | - | - | -0.44 | 0.46 | -0.38 | 0.33 | -0.41 | 0.39 |
| | | | | | | | | |
| N distribution-based PR | 225 | 130 | 181 | 138 | 180 | 129 | 586 | 397 |
| Distribution-based PR | -0.36 | 0.34 | -0.34 | 0.34 | -0.36 | 0.31 | -0.35 | 0.33 |
| N distribution-based RCP | 96 | 89 | 87 | 80 | 77 | 88 | 260 | 257 |
| Distribution-based RCP | -0.19 | 0.19 | -0.26 | 0.23 | -0.27 | 0.20 | -0.24 | 0.21 |
| | | | | | | | | |
| **Moderate change** | | | | | | | | |
| N Anchor-based PR | 51 | 9 | 45 | 7 | 37 | 10 | 133 | 7 |
| Anchor-based PR | -0.86 | - | -0.72 | 1.23 | -0.90 | - | -0.82 | 1.23 |
| N Anchor-based RCP | 5 | 8 | 12 | 9 | 5 | 9 | - | 17 |
| Anchor-based RCP | - | 0.85 | - | - | - | 0.42 | - | 0.62 |
| | | | | | | | | |
| **Large change** | | | | | | | | |
| N Anchor-based PR | 16 | 3 | 12 | 2 | 14 | 3 | 42 | - |
| Anchor-based PR | -0.96 | - | -1.03 | - | -1.18 | - | -1.05 | - |
| N Anchor-based RCP | 4 | 3 | 0 | 2 | 9 | 4 | 9 | 4 |
| Anchor-based RCP | - | - | - | - | -1.12 | 0.98 | -1.12 | 0.98 |
| | | | | | | | | |
| **No change** | | | | | | | | |
| N Anchor-based PR | 133 | | 115 | | 114 | | 362 | |
| Anchor-based PR | -0.07 | | 0.17 | | 0.10 | | 0.06 | |
| N Anchor-based RCP | 141 | | 113 | | 83 | | 337 | |
| Anchor-based RCP | -0.03 | | -0.10 | | -0.04 | | -0.06 | |

Data reported as clinically relevant threshold or N. Negative change represents improvement for all health status instruments. Paired t-tests were applied with significance level at $p < 0.05$. Non- significant results were excluded, except for the "*No change*" group.

*Abbreviations:* CCQ, Clinical COPD Questionnaire; GRC, Global Rating of Change; N, Number of Patients; PR, Pulmonary Rehabilitation; RCP, Routine Clinical Practice; T0, Baseline measurement; T3, Three months follow-up; T6, Six months follow-up; T12, 12 months follow-up.

For peer review only



For peer review only - http://bmjopen.bmj.com/site/about/guidelines.xhtml

*Table 6: Estimates for clinically relevant thresholds for improvement and deterioration on the SGRQ*

| SGRQ | T3-T0 | | T6-T0 | | T12-T0 | | Weighted threshold | |
|---|---|---|---|---|---|---|---|---|
| *Change* | *Improvement* | *Deterioration* | *Improvement* | *Deterioration* | *Improvement* | *Deterioration* | *Improvement* | *Deterioration* |
| | | | | | | | | |
| **Minimal change** | | | | | | | | |
| N Anchor-based PR | 107 | 36 | 96 | 42 | 88 | 43 | 291 | 121 |
| Anchor-based PR | -7.58 | 5.01 | -9.20 | 5.14 | -8.82 | 7.52 | -8.49 | 5.95 |
| N Anchor-based RCP | 12 | 27 | 14 | 36 | 18 | 46 | 14 | 82 |
| Anchor-based RCP | - | - | -4.70 | 7.53 | - | 5.60 | -4.70 | 6.45 |
| | | | | | | | | |
| N distribution-based PR | 237 | 113 | 193 | 119 | 180 | 126 | 610 | 358 |
| Distribution-based PR | -4.83 | 5.09 | -4.94 | 4.46 | -5.22 | 4.47 | -4.98 | 4.66 |
| N distribution-based RCP | 97 | 101 | 75 | 108 | 81 | 92 | 253 | 301 |
| Distribution-based RCP | -2.79 | 2.75 | -2.76 | 3.09 | -4.76 | 3.53 | -3.41 | 3.11 |
| | | | | | | | | |
| **Moderate change** | | | | | | | | |
| N Anchor-based PR | 51 | 9 | 45 | 7 | 37 | 10 | 124 | 10 |
| Anchor-based PR | -15.85 | - | -13.63 | - | -15.40 | 9.30 | -16.06 | 9.30 |
| N Anchor-based RCP | 5 | 8 | 12 | 9 | 5 | 9 | - | 9 |
| Anchor-based RCP | - | - | - | - | - | 7.46 | - | 7.46 |
| | | | | | | | | |
| **Large change** | | | | | | | | |
| N Anchor-based PR | 16 | 3 | 12 | 2 | 14 | 3 | 42 | - |
| Anchor-based PR | -18.33 | - | -21.99 | | -20.58 | - | -20.13 | - |
| N Anchor-based RCP | 4 | 3 | 0 | 2 | 9 | 4 | 9 | - |
| Anchor-based RCP | - | - | - | - | -18.70 | - | -18.70 | - |
| | | | | | | | | |
| **No change** | | | | | | | | |
| N Anchor-based PR | 133 | | 115 | | 114 | | 362 | |
| Anchor-based PR | -1.50 | | -0.99 | | -0.06 | | -0.88 | |
| N Anchor-based RCP | 141 | | 113 | | 83 | | 337 | |
| Anchor-based RCP | 0.51 | | 0.19 | | 0.10 | | 0.30 | |

Data reported as clinically relevant threshold or N. Negative change represents improvement for all health status instruments. Paired t-tests were applied with significance level at p <0.05. Non- significant results were excluded, except for the "*No change*" group.

*Abbreviations:* GRC, Global Rating of Change; N, Number of Patients; PR, Pulmonary Rehabilitation; RCP, Routine Clinical Practice; SGRQ, St. George's Respiratory Questionnaire; T0, Baseline measurement; T3, Three months follow-up; T6, Six months follow-up; T12, 12 months follow-up.

*Figure 1: Forrest plot of clinically relevant thresholds for improvement and deterioration on the CAT.*

*Figure 2: Forrest plot of clinically relevant thresholds for improvement and deterioration on the CCQ.*

*Figure 3: Forrest plot of clinically relevant thresholds for improvement and deterioration on the SGRQ.*

*Legend Figures 1-3: Data are presented as mean estimates (squares) including 95% confidence interval (horizontal lines). Estimates from the half standard deviation analysis are represented as single squares. Weighted mean estimates are presented as larger diamonds. Data are separated as minor, moderate and large improvement thresholds (left half), versus minor and moderate deterioration thresholds (right half).*

For peer review only

# Discussion

*Summary of main findings*

Using both anchor- and distribution-based methods, the *weighted MCIDs* for improvement and deterioration on the CAT were respectively -2.51 vs. 2.76 during PR; and -2.49 vs. 1.65 during Routine Clinical Practice (RCP). These thresholds for improvement and deterioration on the CCQ were respectively -0.40 vs. 0.43 during PR; and -0.33 vs. 0.30 during RCP. MCIDs for the SGRQ were respectively -6.74 vs. 5.31 during PR; and -4.06 vs. 4.78 during RCP for improvement and deterioration. Estimates for minimal clinically important improvement and deterioration were overall somewhat similar, however absolute MCIDs differed between PR and RCP. Thresholds for *moderate* and *large* improvement and deterioration differed from each other, as well as between study settings.

*Interpretation of findings*

Little evidence exists whether MCIDs for improvement are similar for deterioration [21, 23, 40]. Jaeschke et al. were the first to determine the MCID of a health status tool using a 15-point GRC combining both improved and deteriorated COPD patients into one group of minimally changed participants [19]. Juniper et al. elaborated on this by separating minimally improved patients from deterioration in asthma, but only a limited number of patients indicated deterioration and no conclusions upon the MCID of deterioration were drawn [37]. Outside the field of COPD, Crosby et al. and de Vet et al. stated that some studies demonstrated that a smaller MCID for improvement was required compared with deterioration [21, 40]. The current study does not confirm this; although MCIDs seemed smaller for RCP patients compared with PR. Patients experienced more change (hence larger absolute MCIDs) during intervention, possibly as a result of treatment. In RCP, smaller changes may be noted and regarded as relevant for the patient. Overall, the absolute values for the MCIDs for improvement and deterioration did not seem to differ much here, with the exception of the SGRQ during PR.

The ranges found in this study for the MCID of the CAT *(improvement -3.78 to -1.53; deterioration 1.30 to 4.21)* matched with estimates found in other studies [11-15, 20]. Two studies used a patient-assessed GRC to estimate the MCID of the CAT [14-15]. However, no results were reported for worsened patients or the numbers of patients were too few. Other anchor-based methods suggested that a change of one point on the CAT might represent the MCID for deterioration [14]. The weighted thresholds for minimal clinically relevant improvement *(-2.51 in PR and -2.49 in RCP)* seemed somewhat comparable with the ones for deterioration *(2.76 in PR and 1.65 in RCP)* in the current study, except for deterioration during routine clinical practice. As CAT allows only integer scores [2], a change of three points seems a valid threshold for improvement and deterioration, although the MCID for



For peer review only - http://bmjopen.bmj.com/site/about/guidelines.xhtml

deterioration in RCP could be closer to two points. Thresholds for moderate improvement *(-4.23 in PR)* and deterioration *(7.06 in PR and 3.89 in RCP)* turned out less similar. The number of patients moderately deteriorating was low and differences were observed between both study settings. Moderate change might be experienced with a change on the CAT score of 4-7 points. Two previous studies suggested that a cut-off point of four points was identified for acute HRQoL deterioration in clinical practice [41-42]. This would match our estimates for moderate change. The number of patients with a large change was too low with wide confidence intervals to enable valid conclusions.

Regarding the CCQ, the MCID ranges found for both improvement *(-0.50 to -0.19)* and deterioration *(0.19 to 0.66)* overlapped each other in absolute sense, indicating that estimates for improvement and deterioration may be similar. However, differences were noted between PR *(±0.40)* and RCP *(±0.30)* for both minimal improvement and deterioration. These estimates for the MCID matched with earlier evidence [8-13]. One other study used a GRC to determine the MCID of the CCQ [8]. Unfortunately, no data were available on worsening patients. Thresholds for moderate change on the CCQ were broad (±0.62 to ±1.23). Few patients experienced large changes, but estimates for both types of MCID from both study settings were approximately one point.

Minimal thresholds for improvement *(-9.20 to -2.76)* and deterioration *(2.75 to 7.53)* on the SGRQ overlapped each other, although more variation was present here. A change of approximately four to seven points for both improvement and deterioration seemed to be the minimal clinically important threshold in the current study. The MCID for improvement during PR *(-6.74)* was larger than for deterioration *(5.31)*; however, confidence intervals for deterioration were wide. Estimates for the thresholds during RCP (four to five points) were smaller compared with PR (five to seven points). Moreover, the distribution-based estimates turned out smaller than the anchor-based estimates, lowering the absolute weighted MCIDs. Thresholds for moderate improvement and deterioration in the current study were not very similar ranging absolutely from 7.46 to 16.06 points. Estimates for clinically relevant large HRQoL improvement on the SGRQ ranged -20 to -18 points for PR and RC, but too few patients were included to draw valid conclusions.

The SGRQ MCID matched to some extent with previous results [12, 16-18, 20]. Jones et al. published a threshold of four points, which is generally accepted and applied in clinical practice [16, 18]. Interestingly, most results in our current study suggest a larger MCID, although estimates from RCP included this four point's estimate. The estimate by Jones et al. was based upon a study using patient preference-based techniques in COPD by applying a five-point patients' judgement of treatment efficacy (Salmeterol). This MCID of four points was valid for the



For peer review only - http://bmjopen.bmj.com/site/about/guidelines.xhtml

group of patients that experienced effective treatment. In addition, a clinicians' five-point GRC was scored, resulting in a MCID of four points. Clinicians' and patients' ratings are however not necessarily similar [43].

*Strengths and limitations of current study*

This retrospective analysis of two prospective studies was the first to investigate clinically relevant thresholds for minimal, moderate and large changes in COPD health status comparing both improvement and deterioration using a triangulation of both anchor- and distribution-based methods. There were sufficient correlations between the GRC and respective health status questionnaires as required [22]; although they were still only weak to moderate. It should be noted that correlations were stronger with the follow-up score compared with the baseline and/or change score, possibly due to a response shift. Another strength is that multiple follow-up visits were included to limit possible influence of the period of measurements on the MCID and recall bias [21, 24]. Moreover, this study investigated clinically relevant thresholds for both PR and a routine clinical practice, improving its clinical application and external validity.

Although this is the first study to investigate thresholds for clinically relevant deterioration, still a limited number of patients indicated deterioration in HRQoL after PR and during routine clinical practice. This is a major limitation lowering the statistical power of the analysis, especially since sample size calculations were not based upon the separate GRC categories. A second limitation is that the found thresholds demonstrate broad ranges with wide confidence intervals, limiting its accuracy and requiring a larger sample size than our current studies have. Third, it should be taken into account that anchor- and distribution-based approaches each have their own relevance, either based upon clinical retrospective assessments or statistical parameters. It is recommended to combine both methods in measuring an instrument's MCID [22], however estimates are somewhat different between these methods.

*Implications for future research and clinical practice*

COPD patients tend to have worsening HRQoL over time; hence MCIDs for deterioration have an important implication for clinical practice [44-45]. Clinicians and researchers should be able to judge whether groups of patients were really worsening over time or that change observed was subject to random fluctuation. Preventing clinically relevant deterioration in HRQoL by means of therapy is thus an important goal too. Ideally, more research is needed to validate our thresholds for clinically relevant deterioration on the CAT, CCQ and SGRQ for instance in studies other kinds of interventions than PR. One cannot directly transform the thresholds for



For peer review only - http://bmjopen.bmj.com/site/about/guidelines.xhtml

improvement into those for deterioration. Evidence outside the field of COPD has found differences. However, in the current study, the estimates turned out rather similar with differing MCIDs between studies. Setting could thus potentially impact the MCID, implying that the results in the current study not necessarily need to be valid in other settings too.

*Conclusions*

Determining deterioration in HRQoL is of importance, since one needs to differentiate between real worsening of patients' status and random variations. In this study, estimates for clinically relevant thresholds for improvement and deterioration were somewhat similar, but differed between Pulmonary Rehabilitation and Routine Clinical Practice (RCP). We would recommend using cut-points of CAT≥3 (intervention), CAT≥2 (RCP), CCQ ≥0.40 (intervention), CCQ≥0.30 (RCP), SGRQ≥6 (intervention) and SGRQ ≥5 (RCP) for both *minimal* improvement and deterioration. Thresholds for respectively *moderate* and *large* changes should be further explored, but could approximately be in the range of respectively 4-5 and 5-6 for CAT; 0.80 and 1.00 for CCQ; 10-15 points and 15-20 points for SGRQ.

For peer review only

## List of Abbreviations

| | |
|---|---|
| 0.5SD | Half Standard Deviation |
| 95%CI | 95% Confidence Interval |
| CAT | COPD Assessment Test |
| CCQ | Clinical COPD Questionnaire |
| COPD | Chronic Obstructive Pulmonary Disease |
| FEV1%Pred | Forced Expiratory Volume in one second % predicted |
| GOLD | Global initiative for Obstructive Lung Diseases |
| GRC | Global Rating of Change scale |
| HRQoL | Health-Related Quality of Life |
| MCID | Minimal Clinically Important Difference |
| N | Number of Patients |
| PR | Pulmonary Rehabilitation |
| PROs | Patient-Reported Outcomes |
| RCP | Routine Clinical Practice |
| RIMTCORE | Routine Inspiratory Muscle Training within COPD Rehabilitation |
| SD | Standard Deviation |
| SGRQ | St. George Respiratory Questionnaire |
| T0 | Baseline measurement |
| T3 | Time point 3 months follow-up |
| T6 | Time point 6 months follow-up |
| T12 | Time point 12 months follow-up |
| UMCG | University Medical Center Groningen |

## Appendices

Figures 1-3

For peer review only

## References

[1] Global Initiative for Chronic Obstructive Lung Disease (GOLD). Global Strategy for the Diagnosis, Management and Prevention of COPD (2017 Report). Available at http://www.goldcopd.org/gold-2017-global-strategy-diagnosis-management-prevention-copd/. Accessed 17th of July 2017.

[2] Jones, PW, Harding, G, Berry, P et al. Development and first validation of the COPD Assessment Test. Eur Respir J 2009; 34: 648-654.

[3] Van der Molen, T, Willemse, BWM, Schokker, S et al. Development, validity and responsiveness of the Clinical COPD Questionnaire. Health and Quality of Life Outcomes 2003; 1: 13.

[4] Jones, PW, Quirck, FH and Baveystock CM. The St George's Respiratory Questionnaire. Respiratory Medicine 1991; 85 (Supplement B): 25-31.

[5] Cave, J, Atkinson, L, Tsiligianni, IG et al. Assessment of COPD wellness tools for use in primary care: an IPCRG initiative. Int J Chron Obstruct Pulmon Dis 2012; 7: 447-456.

[6] Lozano, R, Naghavi, M, Foreman, K et al. Global and regional mortality from 235 causes of death for 20 age groups in 1990 and 2010: a systematic analysis for the Global Burden of Disease Study 2010. Lancet 2012; 380: 2095-2128.

[7] Adeloye, D, Chua, S, Lee, C et al. Global and regional estimates of COPD prevalence: Systematic review and meta-analysis. J Glob Health 2015; 5 (2): 020415.

[8] Kocks, JW, Tuinenga, MG, Uil, SM et al. Health status measurement in COPD: the minimal clinically important difference of the clinical COPD questionnaire. Respir Res 2006; 7: 62.

[9] Kon, SS, Dilaver, D, Mittal, M et al. The Clinical COPD Questionnaire: response to pulmonary rehabilitation and minimal clinically important difference. Thorax 2014; 69 (9): 793-798.

[10] Canavan, JL, Dilaver, D, Clark, AL et al. Clinical COPD Questionnaire in patients with chronic respiratory disease. Respirology 2014; 19 (7): 1006-1012.

[11] Tsiligianni, IG, van der Molen, T, Moraitaki, D et al. Assessing health status in COPD. A head-to-head comparison between the COPD assessment test (CAT) and the clinical COPD questionnaire (CCQ). BMC Pulmonary Medicine 2012; 12: 20.



For peer review only - http://bmjopen.bmj.com/site/about/guidelines.xhtml

[12] Alma, H, de Jong, C, Jelusic, D et al. Health status instruments for patients with COPD in pulmonary rehabilitation: defining a minimal clinically important difference. Npj PCRM 2016; 26: 16041.

[13] Smid, DE, Franssen, FM, Houben-Wilke, S et al. Responsiveness and MCID Estimates for CAT, CCQ and HADS in Patients With COPD Undergoing Pulmonary Rehabilitation: A Prospective Analysis. J Am Med Dir Assoc 2017; 18 (1): 53-58.

[14] Kon, SS, Canavan, JL, Nolan, CM et al. Minimum clinically important difference for the COPD Assessment Test: a prospective analysis. Lancet Respir Med 2014; 2: 195-203.

[15] Dodd, JW, Hogg, L, Nolan, J et al. The COPD assessment test (CAT): response to pulmonary rehabilitation. A multicentre, prospective study. Thorax 2011; 66: 425-429.

[16] Jones, PW. St. George's Respiratory Questionnaire: MCID. COPD 2005; 2: 75-79.

[17] Welling, JB, Hartman, JE, Ten Hacken, NH et al. The minimal important difference for the St George's Respiratory Questionnaire in patients with severe COPD. Eur Respir J 2016; 46: 1598.

[18] Jones, PW and Bosh, TK. Quality of life changes in COPD patients treated with salmeterol. Am J Respir Crit Care Med 1997; 155: 1283-1289.

[19] Jaeschke, R, Singer, J and Guyatt, GH. Measurement of health status. Ascertaining the minimal clinically important difference. Control Clin Trials 1989; 10: 407-415.

[20] Alma, H, de Jong, C, Tsiligianni, I et al. Clinically relevant differences in COPD health status: systematic review and triangulation. Eur Respir J 2018; 6: 52 (3).

[21] Crosby, RD, Kolotkin, RL and Williams, GR. Defining clinically meaningful change in health-related quality of life. Journal of Clinical Epidemiology 2003; 56: 395-400.

[22] Revicki, D, Hays, RD, Cella, D and Sloan, J. Recommended methods for determining responsiveness and minimally important differences for patient-reported outcomes. J Clin Epidemiol 2008; 61: 102-109.

[23] Beaton, DE, Boers, M and Wells, GA. Many faces of the minimal clinically important difference (MCID): a literature review and directions for future research. Curr Opin Rheumatol 2002; 14: 109-114.

[24] Hays, RD and Woolley, M. The Concept of Clinically Meaningful Difference in Health-Related Quality of-Life Research How Meaningful is it? Pharmacoeconomics 2000; 18 (5): 419-423.



For peer review only - http://bmjopen.bmj.com/site/about/guidelines.xhtml

[25] Raman, S, Ding, K, Chow, E et al. Minimal clinically important differences in the EORTC QLQ-BM22 and EORTC QLQ-C15-PAL modules in patients with bone metastases undergoing palliative radiotherapy. Qual Life Res 2016; 25 (10): 2535-2541.

[26] Maringwa, J, Quinten, C, King, M et al. Minimal clinically meaningful differences for the EORTC QLQ-C30 and EORTC QLQ-BN20 scales in brain cancer patients. Ann Oncol 2011; 22 (9): 2107-2112.

[27] Hui, D, Shamieh, O, Paiva, CE et al. Minimal Clinically Important Difference in the Physical, Emotional, and Total Symptom Distress Scores of the Edmonton Symptom Assessment System. J Pain Symptom Manage 2016; 51 (2): 262-269.

[28] Tuomi, L, Johansson, M, Andréll, P and Finizia, C. Interpretation of the Swedish Self Evaluation of Communication Experiences after Laryngeal cancer: Cutoff levels and minimum clinically important differences. Head Neck 2016; 38 (5): 689-695.

[29] Rouquette, A, Blanchin, M, Sébille, V et al. The minimal clinically important difference determined using item response theory models: an attempt to solve the issue of the association with baseline score. J Clin Epidemiol 2014; 67 (4): 433-440.

[30] Hui, D, Shamieh, O, Paiva, CE et al. Minimal clinically important differences in the Edmonton Symptom Assessment Scale in cancer patients: A prospective, multicentre study. Cancer 2015; 121 (17): 3027-3035.

[31] Singh, D, D'Urzo, AD, Chuecos, F et al. Reduction in clinically important deterioration in chronic obstructive pulmonary disease with aclidinium/formoterol. Respiratory Research 2017; 18: 106.

[32] Stockley, RA, Edgar, RG, Starkey, S and Turner AM. Health status decline in α-1 antitrypsin deficiency: a feasible outcome for disease modifying therapies? Respir Res 2018; 19 (1): 137.

[33] Anzueto, AR, Kostikas, K, Mezzi, K et al. Indacaterol/glycopyrronium versus salmeterol/fluticasone in the prevention of clinically important deterioration in COPD: results from the FLAME study. Respir Res 2018; 19 (1): 121.

[34] Naya, IP, Tombs, L, Muellerova, H et al. Long-term outcomes following first short-term clinically important deterioration in COPD. Respir Res 2018; 19 (1): 222.

[35] Schultz, K, Jelusic, D, Wittmann, M et al. Inspiratory Muscle Training does not improve clinical outcomes in 3-week COPD rehabilitation: Results from a Randomised Controlled Trial. Eur Respir J 2018; 51 (1): 1702000.



For peer review only - http://bmjopen.bmj.com/site/about/guidelines.xhtml

[36] Kamper, SJ, Maher, CG and Mackay, G. Global rating of change scales: a review of strengths and weaknesses and considerations for design. J Man Manip Ther 2009; 17: 163-170.

[37] Juniper, EF, Guyatt, GH, Willan, A et al. Determining a Minimal Important Change in a Disease-Specific Quality of Life Questionnaire. J Clin Epidemiol 1994; 47 (I): 81-87.

[38] Norman, GR, Sloan, JA and Wyrwich, KW. Interpretation of changes in health-related quality of life: the remarkable universality of half a standard deviation. Med Care 2003; 41: 582-592.

[39] Terwee, CB, Bot SD, de Boer, MR et al. Quality criteria were proposed for measurement properties of health status questionnaires. J Clin Epidemiol 2007; 60: 34-42.

[40] de Vet, HC, Terwee, CB, Ostelo, RW et al. Minimal changes in health status questionnaires: distinction between minimally detectable change and minimally important change. Health and Quality of Life Outcome 2006; 4: 54.

[41] Pothirat, C, Chaiwong, W, Limsukon, A et al. Detection of acute deterioration in health status visit among COPD patients by monitoring COPD assessment test score. International Journal of COPD 2015; 10: 277-282.

[42] García-Sidro, P, Naval, E, Martinez Rivera, C et al. The CAT (COPD Assessment Test) questionnaire as a predictor of the evolution of severe COPD exacerbations. Respir Med 2015; 109 (12): 1546-1552.

[43] Janse, AJ, Gemke, RJ, Uiterwaal, CS et al. Quality of life: patients and doctors don't always agree: a meta-analysis. J Clin Epidemiol 2004; 57 (7): 653-661.

[44] Yoo, JY, Kim, YS, Kim, SS et al. Factors affecting the trajectory of health-related quality of life in COPD patients. Int J Tuberc Lung Dis 2016; 20 (6): 738-746.

[45] Sundh, J, Montgomery, S, Hasselgren, M et al. Change in health status in COPD: a seven-year follow-up cohort study. npj Primary Care Respiratory Medicine 2016; 26: 16073.

For peer review only

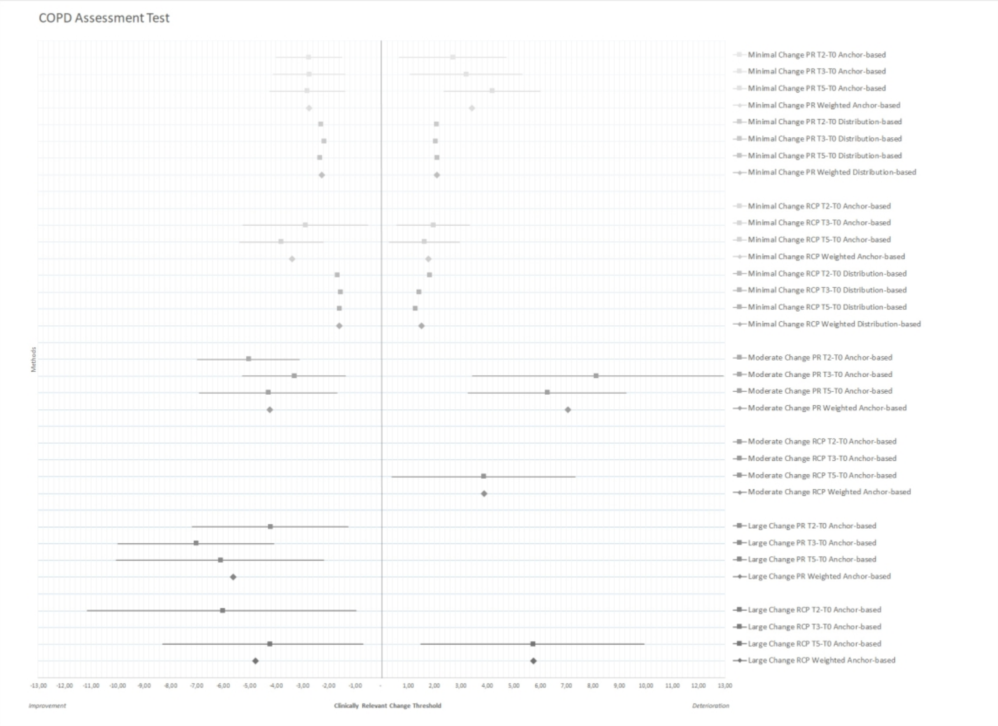


Caption: Figure 1: Forrest plot of clinically relevant thresholds for improvement and deterioration on the CAT.

Legend: Data are presented as mean estimates (squares) including 95% confidence interval (horizontal lines). Estimates from the half standard deviation analysis are represented as single squares. Weighted mean estimates are presented as larger diamonds. Data are separated as minor, moderate and large improvement thresholds (left half), versus minor and moderate deterioration thresholds (right half).

159x115mm (300 x 300 DPI)

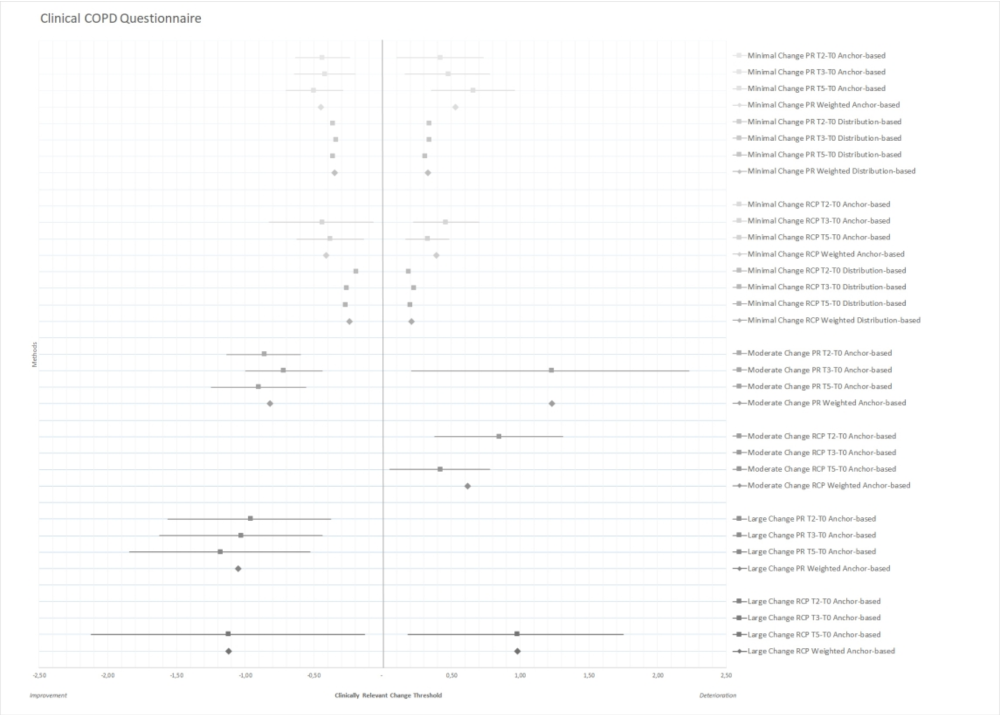


Caption: Figure 2: Forrest plot of clinically relevant thresholds for improvement and deterioration on the CCQ.

Legend: Data are presented as mean estimates (squares) including 95% confidence interval (horizontal lines). Estimates from the half standard deviation analysis are represented as single squares. Weighted mean estimates are presented as larger diamonds. Data are separated as minor, moderate and large improvement thresholds (left half), versus minor and moderate deterioration thresholds (right half).

159x114mm (300 x 300 DPI)

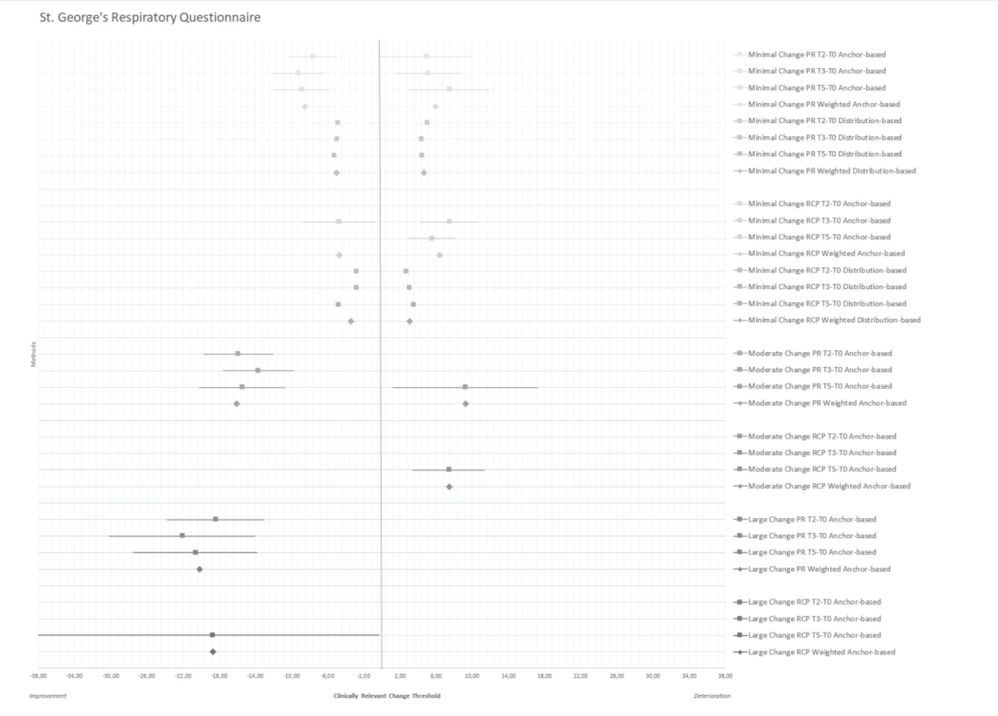


Caption: Figure 3: Forrest plot of clinically relevant thresholds for improvement and deterioration on the SGRQ.

Legend: Data are presented as mean estimates (squares) including 95% confidence interval (horizontal lines). Estimates from the half standard deviation analysis are represented as single squares. Weighted mean estimates are presented as larger diamonds. Data are separated as minor, moderate and large improvement thresholds (left half), versus minor and moderate deterioration thresholds (right half).

160x114mm (300 x 300 DPI)

STROBE Statement—checklist of items that should be included in reports of observational studies

| | Item No. | Recommendation | Page No. | Relevant text from manuscript |
|---|---|---|---|---|
| **Title and abstract** | 1 | (*a*) Indicate the study's design with a commonly used term in the title or the abstract | 0 | Title page |
| | | (*b*) Provide in the abstract an informative and balanced summary of what was done and what was found | 1 | Abstract page |
| **Introduction** | | | | |
| Background/rationale | 2 | Explain the scientific background and rationale for the investigation being reported | 6-7 | Introduction pages lines 1-36 |
| Objectives | 3 | State specific objectives, including any prespecified hypotheses | 7 | Introduction pages lines 29-36 |
| **Methods** | | | | |
| Study design | 4 | Present key elements of study design early in the paper | 8 | Methods lines 39-52 |
| Setting | 5 | Describe the setting, locations, and relevant dates, including periods of recruitment, exposure, follow-up, and data collection | 8 | Methods lines 39-52 |
| Participants | 6 | (*a*) *Cohort study*—Give the eligibility criteria, and the sources and methods of selection of participants. Describe methods of follow-up<br>*Case-control study*—Give the eligibility criteria, and the sources and methods of case ascertainment and control selection. Give the rationale for the choice of cases and controls<br>*Cross-sectional study*—Give the eligibility criteria, and the sources and methods of selection of participants | 8 | Methods lines 39-52 |
| | | (*b*) *Cohort study*—For matched studies, give matching criteria and number of exposed and unexposed<br>*Case-control study*—For matched studies, give matching criteria and the number of controls per case | N/A | N/A |
| Variables | 7 | Clearly define all outcomes, exposures, predictors, potential confounders, and effect modifiers. Give diagnostic criteria, if applicable | 8-9 | Methods lines 56-80 |
| Data sources/ measurement | 8* | For each variable of interest, give sources of data and details of methods of assessment (measurement). Describe comparability of assessment methods if there is more than one group | 8-9 | Methods lines 56-80 |
| Bias | 9 | Describe any efforts to address potential sources of bias | 3 | Text on competing interests |
| Study size | 10 | Explain how the study size was arrived at | 1 | Sample size calculations are presented in the original study |


For peer review only - http://bmjopen.bmj.com/site/about/guidelines.xhtml

| | | | | |
|---|---|---|---|---|
| | | | | protocols (see trial reference numbers in the abstract) |
| Quantitative variables | 11 | Explain how quantitative variables were handled in the analyses. If applicable, describe which groupings were chosen and why | 9 | Methods lines 81-94 |
| Statistical methods | 12 | (*a*) Describe all statistical methods, including those used to control for confounding | 10 | Methods lines 95-123 |
| | | (*b*) Describe any methods used to examine subgroups and interactions | 10 | Methods lines 95-123 |
| | | (*c*) Explain how missing data were addressed | 12 | Results lines 140-141 |
| | | (*d*) *Cohort study*—If applicable, explain how loss to follow-up was addressed<br>*Case-control study*—If applicable, explain how matching of cases and controls was addressed<br>*Cross-sectional study*—If applicable, describe analytical methods taking account of sampling strategy | N/A | N/A |
| | | (*e*) Describe any sensitivity analyses | N/A | N/A |
| **Results** | | | | |
| Participants | 13* | (a) Report numbers of individuals at each stage of study—eg numbers potentially eligible, examined for eligibility, confirmed eligible, included in the study, completing follow-up, and analysed | 11 | Results lines 126-131 |
| | | (b) Give reasons for non-participation at each stage | 11 | Results lines 126-131 |
| | | (c) Consider use of a flow diagram | NA | N/A |
| Descriptive data | 14* | (a) Give characteristics of study participants (eg demographic, clinical, social) and information on exposures and potential confounders | 11 | Results Table 1 and lines 132-135 |
| | | (b) Indicate number of participants with missing data for each variable of interest | - | In the results section the number of participants is noted under N. This indirectly gives notice of the missing data when deducted from the number of patients at each follow-up visit. |
| | | (c) *Cohort study*—Summarise follow-up time (eg, average and total amount) | 12-13 | Results lines 139-153 and table 2 |
| Outcome data | 15* | *Cohort study*—Report numbers of outcome events or summary measures over time | 12-13 | Results lines 139-153 and table 2 |
| | | *Case-control study*—Report numbers in each exposure category, or summary measures of exposure | 12-13 | Results lines 139-153 and table 2 |
| | | *Cross-sectional study*—Report numbers of outcome events or summary measures | 12-13 | Results lines 139-153 and table 2 |


For peer review only - http://bmjopen.bmj.com/site/about/guidelines.xhtml

| | | | | |
|---|---|---|---|---|
| Main results | 16 | (*a*) Give unadjusted estimates and, if applicable, confounder-adjusted estimates and their precision (eg, 95% confidence interval). Make clear which confounders were adjusted for and why they were included | 14-17 | Results lines 166-176 and tables 4-6 and figures 1-3 |
| | | (*b*) Report category boundaries when continuous variables were categorized | N/A | N/A |
| | | (*c*) If relevant, consider translating estimates of relative risk into absolute risk for a meaningful time period | N/A | N/A |
| Other analyses | 17 | Report other analyses done—eg analyses of subgroups and interactions, and sensitivity analyses | N/A | N/A |
| **Discussion** | | | | |
| Key results | 18 | Summarise key results with reference to study objectives | 18 | Discussion lines 194-201 |
| Limitations | 19 | Discuss limitations of the study, taking into account sources of potential bias or imprecision. Discuss both direction and magnitude of any potential bias | 20 | Discussion lines 253-271 |
| Interpretation | 20 | Give a cautious overall interpretation of results considering objectives, limitations, multiplicity of analyses, results from similar studies, and other relevant evidence | 18-19 | Discussion lines 202-252 |
| Generalisability | 21 | Discuss the generalisability (external validity) of the study results | 20-21 | Discussion lines 273-282, Conclusions lines 283-291 |
| **Other information** | | | | |
| Funding | 22 | Give the source of funding and the role of the funders for the present study and, if applicable, for the original study on which the present article is based | 3 | Text on funding |

*Give information separately for cases and controls in case-control studies and, if applicable, for exposed and unexposed groups in cohort and cross-sectional studies.

**Note:** An Explanation and Elaboration article discusses each checklist item and gives methodological background and published examples of transparent reporting. The STROBE checklist is best used in conjunction with this article (freely available on the Web sites of PLoS Medicine at http://www.plosmedicine.org/, Annals of Internal Medicine at http://www.annals.org/, and Epidemiology at http://www.epidem.com/). Information on the STROBE Initiative is available at www.strobe-statement.org.

For peer review only
